# Combining AlphaFold with Focused Virtual Library Design in the Development of Novel CCR2 and CCR5 Antagonists

**DOI:** 10.1021/acs.jcim.5c01596

**Published:** 2025-11-12

**Authors:** Khaled Essa, Kian Noorman van der Dussen, Yao Yao, Bente Bleijs, Natalia V. Ortiz Zacarias, Laura H. Heitman, Gerard J.P. van Westen, Willem Jespers, Daan van der Es, Martin Šícho

**Affiliations:** † Division of Medicinal Chemistry, Leiden Academic Centre for Drug Research (LACDR), 4496 Leiden University, 2333 CC Leiden, The Netherlands; ‡ Oncode Institute, 2333 CC Leiden, The Netherlands; § Department of Medicinal Chemistry, Photopharmacology and Imaging, Groningen, Research Institute of Pharmacy (GRIP), Faculty of Science and Engineering, Antonius Deusinglaan 1, 9713 AV Groningen, The Netherlands; ∥ CZ-OPENSCREEN: National Infrastructure for Chemical Biology, Department of Informatics and Chemistry, Faculty of Chemical Technology, University of Chemistry and Technology Prague, Technická 5, 166 28, Prague, Czech Republic

## Abstract

CC chemokine receptor (CCR) 2 and 5 are G protein-coupled receptors that play a crucial role in immunohomeostasis. Accordingly, overactivation of their signaling pathways is involved in various immunopathologies and cancer. Extensive research focusing on discovering CCR2 and CCR5 orthosteric antagonists, ultimately resulted in some clinical success, but the area of intracellular allosteric modulators is still underexplored and the move from orthosteric to allosteric modulation could be an interesting paradigm shift. To this end, we document the development of novel CCR2 and CCR5 intracellular allosteric antagonists through a virtual screen on a small combinatorial library derived from existing CCR2, CCR5, and CCR4 ligands. Using a molecular docking approach, the created library was screened in its entirety utilizing a refined AlphaFold model of CCR5 based on the crystal structure of its close homologue, CCR2. The screening resulted in the identification of several virtual hits, out of which one was developed further by in-house synthesis. In total, 18 analogues were prepared and experimentally evaluated for their binding affinity for CCR2 and functional inhibition on CCR5. This expeditious and simple workflow beginning from docking to compound evaluation identified 3 hits for CCR2 (*K*_i_ = 1.3–6 μM) and 1 hit (IC_50_ = 10.8 μM) for CCR5. The obtained structure–activity relationships were also further rationalized using structural information available for both CCR5 and CCR2 providing valuable insights for future development of intracellular allosteric ligands.

## Graphical abstract

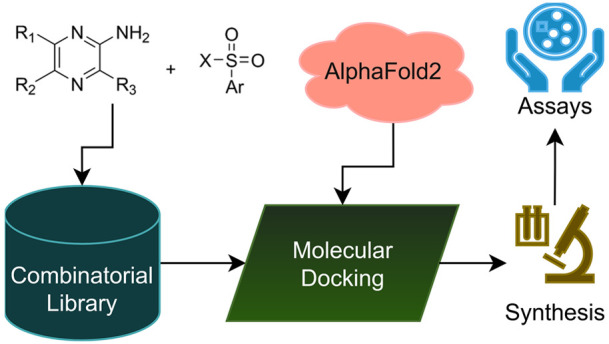

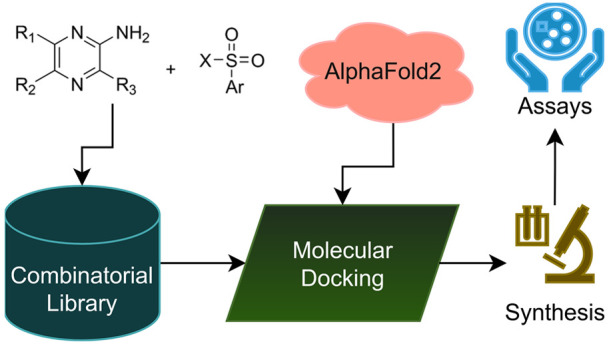

## Introduction

Immune responses are crucial actions carried out by the immune system to maintain physiological homeostasis and defend the host against invading pathogens. Chemokine receptors are among crucial players in these immune responses as they contribute to the regulation of migration (chemotaxis) and activation of leukocytes.^1^ Chemokine receptors belong to the family of class A seven-transmembrane G Protein-Coupled Receptors (GPCRs).^2^ Among these receptors, the C–C Chemokine Receptor 5 (CCR5) and the closely homologous (>76% overall homology^56^) Chemokine Receptor 2 (CCR2) are among key players in modulating immune responses. These chemokine receptors are located on the cell membranes of various immune cells, including T cells, macrophages, and dendritic cells.^3–7^ This localization allows CCR2 and CCR5 to respond to signaling cues of chemokines that guide immune cell recruitment to inflamed tissues. A defining characteristic of chemokine receptors is their versatility in interacting with a spectrum of chemokines. CCR5 binds to CCL3 (MIP-1α), CCL4 (MIP-1β), and CCL5 (RANTES),^8,9^ while CCR2 binds CCL2, CCL7, CCL8, and CCL13.^10^ Such intricate involvement of CCR2 and CCR5 in homeostatic immune surveillance and dynamic responses to tissue injury or infection positions them as critical factors in various immunological disorders.

Although novel antagonists for CCR2 and CCR5 have yielded a wealth of structural insights and the potential utility of their antagonism was demonstrated, challenges persist.^11^ Despite these shortcomings, much of the research so far has still focused on targeting the extracellular orthosteric binding site of CCR2 and CCR5, but previous findings highlight the need for alternative strategies, and a novel frontier lies in exploring the intracellular allosteric binding site.^12^ Unlike orthosteric ligands, allosteric ligands do not compete with endogenous chemokines which is a distinct advantage of allostery. This characteristic is particularly beneficial in disorders where elevated concentrations of chemokines increase the competition with orthosteric antagonists.^12^ To date, only a handful of single or dual allosteric intracellular CCR2 and CCR5 antagonists have been developed.^13,14^ Accordingly, this research area demands further exploration and especially the utilization of computational methods using structural information has been limited.

Structure-based computational methods have been used previously to describe the binding of both orthosteric and allosteric modulators of CCR5 and CCR2. Both recent^15–18^ and past^19–24^ studies focused on the extracellular orthosteric binding site. Although structure-based modeling was also shown to be instrumental in studies focusing on the allosteric intracellular binding site of both CCR5 ^25^ and its closest homologue CCR2,^26^ there were no attempts to exploit structural information about the intracellular allosteric binding site of CCR2 or CCR5 in a virtual screening and *de novo* drug design setting, yet.

The use of large combinatorial on-demand libraries for virtual screening has been on the rise.^27^ However, exhaustively screening such large libraries would require considerable computational resources,^28^ which might require an investment in the range of hundreds of thousands of dollars per exercise.^29^ Therefore, it is common that only subsets of these libraries are screened in practice (i.e., Enamine Real Subsets^30^), but it should be noted that several optimization strategies have been developed in the past few years that significantly reduce the problem. These methods for example include hierarchical approaches that can employ synthon-based docking^29^ or innovative graph-based data structures.^31^ The use of machine learning is also popular with most approaches implementing an active learning component.^32–34^ It is also notable that advances were also made in GPU-accelerated docking,^35–37^ which makes exhaustive docking screens of large libraries more feasible as well. Finally, it is also not uncommon to utilize in-house combinatorial libraries, which may prove more cost- and time-effective.^38,39^ Therefore, depending on the needs of a particular project, one must find a balance between complexity and potential gains resulting from a large-scale virtual screening campaign.^40^

The usefulness of AlphaFold and similar deep learning protein prediction models in virtual screening has been widely analyzed and contested.^41^ AlphaFold predictions should not be taken at face value, especially when it comes to more subtle side-chain orientations relevant in molecular docking experiments.^41^ However, these predictions can still be useful starting points for refinement with physics-based molecular mechanics methods, in which case more reliable positioning of the side chains can be obtained. It has been shown that even more accurate and structurally sensitive simulations such as FEP can be applied using AlphaFold predictions.^42^ Therefore, AlphaFold-derived structures can be highly useful if appropriate measures are taken.

In the current study, we performed an expeditious and simple virtual screening campaign on a focused in-house combinatorial library of compounds. The approach utilized molecular docking into the intracellular allosteric pocket of CCR5 as predicted by AlphaFold and refined with molecular dynamics simulation. The combinatorial library of putative ligands was inspired by a previously published compound with the pyrazinyl-sulfonamide scaffold, which was determined as moderately active on wild type CCR5 with pIC_50_ of 6.1 ± 0.91 (compound 2 in ref,^43^ labeled as **CP2** throughout this manuscript). In the virtual screening step, a potentially active derivative (later evaluated as LUF8071 **(9**)) was identified and used further as a starting point for a new series of pyrazinyl-sulfonamide compounds. Alongside LUF8071 **(9**), 17 of its derivatives were synthesized. A combination of a CCR2 radioligand binding assay and a CCR5 β-arrestin recruitment assay identified several of the synthesized compounds as potentially selective with good binding to CCR2 and one ligand with moderate activity toward CCR5. In addition to the results presented herein, we also provide the code to perform both library generation and molecular docking in the associated data package.

## Materials and Methods

### Computational Methods

We obtained the raw AlphaFold model of CCR5 from the EBI AlphaFold database, entry A0A089G6S6.^44^ The residues within the pocket were defined based on the CCR2 reference structure and a single adjustment was made: In the original CCR5 model, the Lys59^1.48^ (according to Ballesteros-Weinstein numbering) residue was rotated toward the expected location of the ligand based on the assumptions from the CCR2 crystal structure with CCR2-RA-[R].^45^ This potential clash with the ligand was amended by generating rotamers of Lys59^1.48^ in the CCR5 model with the PyMol software and selecting the one closest to the CCR2 reference.^45^ The updated structure was then used for docking and subsequent refinement with MD simulations as outlined below. The structure of CCR2 and its associated ligand used in all experiments and throughout the manuscript corresponds to PDB entry 5T1A.^45^ However, the original PDB entry was modified for the docking experiments in this study. The lysozyme, ligands and water molecules were removed. Mutated residues as compared to wild type CCR2 were reversed and their rotamers were selected using the mutagenesis features of the PyMol program and manual refinement was performed. The mutagenesis feature was also used in the same way for residues with incomplete atom information. It should be noted that these adjustments were not necessary for any of the residues near the intracellular binding pocket, which was well represented in the deposited PDB file. The modified structure is included in the data package deposited on Zenodo.^46^

Molecular docking was done with AutoDock Vina version 1.2.3.^47^ The 3D coordinates of the amino acid chain of the CCR5 reference model were obtained from molecular dynamics simulations with **CP2** as docked into the refined binding pocket of the AlphaFold structure described above. In this case a snapshot was taken after stabilization of the complex at 87.90 ns at one of the replicas (seed 1867). In all docking experiments (including virtual screening) the exhaustiveness parameter was set to 8 and random seed to 42 for the Vina software, all other parameters were left at their default values for the version used (v1.2.3).^46^ Unless stated otherwise, only the first docking pose with the lowest score was considered each time for further analysis. The combinatorial library for virtual screening was generated with original code implemented by the authors using the RDKit cheminformatics toolkit.^48^ The building blocks used to perform virtual reactions were retrieved from the ZINC15 database using its web-based SMARTS query search tool. The structures of the products within the subsequently generated combinatorial library were then processed with the Dimorphite-DL package with pH set to the 6.0–7.2 range (max_variants = 128, pka_precision = 1.0) to generate likely protonation states for each compound. To maximize reproducibility, only one single initial conformer was generated with the RDKit toolkit using a random seed before it was submitted to AutoDock Vina for pose generation. The code and all input (including the extracted building blocks) and output files needed to reproduce all virtual screening steps from library generation to molecular docking are deposited on Zenodo with documentation and license information included.^46^

Molecular dynamics simulations were conducted with the Desmond program provided in Schrodinger Suite version 2021–2. The starting points of all simulations with novel ligands were the poses obtained by docking each ligand into the CCR5 reference model as described previously. Each molecular dynamics simulation was run in triplicates of length 100 ns each. In all simulations, an explicit solvation model was used, and the protein was embedded in a membrane. All configuration, input, output and project files are provided in the associated data package archived on Zenodo.^46^

### Chemistry Methods

All solvents and reagents were purchased from commercial sources and were used without further purification. ^1^H and ^13^C spectra were recorded on a Bruker AV 400 MHz liquid, Bruker AV 400 MHz wide bore, Bruker AV 500 MHz, or Bruker AV 600 MHz at room temperature (rt) using CDCl_3_, MeOD, or DMSO as a solvent. Chemical shifts are reported in ppm relative to internal standard tetramethylsilane (TMS) or solvent resonance. Purity of the compounds was determined by HPLC with a C18 column (50 × 4.6 mm, 3 μm), flow rate = 1.3 mL/min, using a gradient of 10–90% MeCN/H_2_O (0.1% FA) and measuring UV absorbance at 254 nm. Purity of all final compounds used in biological assays was at least 95% as confirmed by HPLC analysis. Reactions were monitored by TLC using Merck TLC Silica gel 60 F254 aluminum sheets. Compounds were visualized by UV irradiation or by staining with a KMnO_4_ solution in H_2_O. For the flash chromatography, Davisil silica gel (40–63 μm) was used. The automatic flash chromatography was performed on an Isolera One Automatic Flash Chromatography System by Biotage with prepacked flash cartridges (Biotage Sfar D). Automated reverse phase chromatography was performed using HPLC-grade acetonitrile and demi water, both containing 0.1% TFA as the eluent system. Mass spectra were measured using a Shimadzu Prominence LC-MS-2020 system and a Gemini C18 Phenomenex column (50 × 3 mm, 3 μm). Complete information regarding synthetic procedures and obtained spectra to be found in the .

### General Procedure 1 (GP1)

To a 10 mL flask, 6-chloropyrazin-2-amine (1 equiv) was added followed by corresponding amine (2 equiv). The reaction mixture was stirred at 100 °C for 18h. Upon reaction completion, the mixture was diluted with water and extracted with EtOAc. The organic layer was dried over MgSO_4_ then concentrated under reduced pressure. The crude mixture was purified by flash column chromatography using a gradient elution of 70–100% EtOAc/petroleum ether.

### General Procedure 2 (GP2)

A 10 mL flask was charged with the corresponding pyrazine-amine scaffold (1 equiv) and DMAP (0.1–0.2 equiv) dissolved in pyridine (0.05M). The corresponding sulfonyl chloride (1.2–2 equiv) dissolved in pyridine (0.05M) was added dropwise. The reaction was stirred at rt for 18h. Upon reaction completion, pyridine was evaporated under reduced pressure and the residue was taken up in EtOAc and washed three times with 1 M HCl. The organic layer was dried over MgSO_4_ then concentrated under reduced pressure. The crude mixture was purified by flash column chromatography using a gradient elution of 3–6% MeOH/DCM.

### General Procedure 3 (GP3)

A 10 mL flask was charged with 6-(piperidin-1-yl)­pyrazin-2-amine (1 equiv) dissolved in pyridine (2 equiv) and DCM (0.05M) and cooled to 0 °C. The corresponding sulfonyl chloride (2 equiv) dissolved in DCM (0.05M) was added dropwise to the amine mixture under inert conditions. The reaction mixture was allowed to heat to rt and stirred for 18h. Upon reaction completion, the reaction mixture was diluted in ethyl acetate and washed twice with 1 M HCl. The aqueous layer was back extracted thrice with ethyl acetate. The combined organic layers were dried over MgSO_4_, filtered and concentrated under reduced pressure. The crude mixture was purified by flash column chromatography using a gradient elution of 2–6% MeOH/DCM.

### Biological Evaluation Methods

Both U2OS-CCR2_bla and U2OS-CCR5_bla cells were cultured in McCoy’s 5A medium supplemented with 10% (v/v) fetal calf serum, 2 mM glutamine, 0.1 mM nonessential amino acids, 25 mM HEPES, 1 mM sodium pyruvate, 200 IU/mL penicillin, 200 μg/mL streptomycin, 100 μg/mL G418, 50 μg/mL hygromycin, and 125 μg/mL zeocin in at humidified atmosphere at 37 °C and 5% CO2. Cells were grown until 80% confluence and cultured twice weekly on 10 or 15 cm ⌀ plates by trypsinization. Dialyzed fetal calf serum was used when culturing cells for functional assays or as a last step before membrane preparation.

Membranes from U2OS-CCR2_bla cells were prepared as previously described for CCR2.^49^ Briefly, U2OS-CCR2_bla cells were scraped from confluent 15 cm ⌀ plates using phosphate-buffered saline (PBS) and subsequently centrifuged at 3000 rpm for 5 min. Pellets were then resuspended in ice-cold Membrane preparation buffer (50 mM Tris-HCl, 5 mM MgCl_2_, pH 7.4) before homogenization with an Ultra Turrax homogenizer (IKA-Werke GmbH & Co. KG, Staufen, Germany). Membranes and cytosolic contents were separated using an Optima LE-80 K ultracentrifuge (Beckman Coulter, Inc., Fullerton, CA) at 31 000 rpm for 20 min at 4 °C. After a second cycle of homogenization and centrifugation, the final pellet was resuspended and homogenized in ice-cold Membrane preparation buffer, aliquoted, and stored at −80 °C. Finally, membrane protein concentrations were determined using a BCA protein determination assay, as described by the manufacturer (Pierce BCA protein assay kit).^50^

For [^3^H]-CCR2-RA-[*R*] displacement assays, U2OS-CCR2_bla membrane homogenates (15–20 μg of total protein) were incubated with ∼ 6 nM [^3^H]-CCR2-RA-[*R*] and at least 6 increasing concentrations of competing ligand in a final volume of 100 μL of assay buffer (50 mM Tris-HCl, 5 mM MgCl_2_, 0.1% CHAPS, pH 7.4). Ligands were diluted to the desired concentration with an HP D300 digital dispenser (Tecan, Giessen, The Netherlands). Total radioligand binding did not exceed 10% of the amount added to prevent ligand depletion, and nonspecific binding was determined using 10 μM CCR2-RA-[*R*]. After 2 h at 25 °C incubation was terminated by rapid filtration through a 96-well GF/C filter plate (Revvity, Groningen, The Netherlands) on a FilterMate harvester (Revvity, Groningen, The Netherlands), using ice-cold wash buffer (50 mM Tris-HCl buffer supplemented with 5 mM MgCl_2_ and 0.05% CHAPS, pH 7.4). Filters were washed 20 times with ice-cold wash buffer and subsequently dried at 55 °C for 30 min. After the addition of 25 μL of Microscint scintillation cocktail (Revvity, Groningen, The Netherlands), the filter-bound radioactivity was measured by scintillation spectrometry using the P-E 2450 Microbeta^2^ counter (Revvity, Groningen, The Netherlands).

β-Arrestin recruitment was measured using the Tango CCR5-bla U2OS cell-based assay (Invitrogen) according to the manufacturer’s protocol.^51^ Briefly, U2OS-CCR5_bla cells were grown until approximately 80% confluence and detached by trypsinization. Cells were recovered by centrifugation at 1000 rpm for 5 min, resuspended in assay medium (FreeStyle Expression Medium, Invitrogen) to a density of 40 000 cells per well, and seeded into black-wall, clear-bottom, 96-well assay plates (Corning). For agonist assays, cells were exposed to increasing concentrations of CCL3 (MIP-1α) for CCR5, respectively, for 16 h at 37 °C and 5% CO_2_. For antagonist assays, compounds were first diluted in an assay medium containing a final DMSO concentration of 0.5% or lower. Cells were then preincubated with either 10 μM (for single-point inhibition experiments) or increasing concentrations of antagonist (0.1 μM to 0.1 mM) for 30 min at room temperature, before a 16-h coincubation with an EC_80_ concentration of CCL3 (5.15 nM) at 37 °C and 5% CO_2_. After 16 h, cells were loaded in the dark with 32 μL of 6x Substrate Mixture (For 1 mL Substrate Mixture: 6 μL of 1 mM LiveBLAzer-FRET B/G (CCF4-AM), 60 μL Solution B, 904 μL Solution C, and 30 μL Solution D, as described in the manufacturer’s protocol,^51^ followed by 2 h incubation at room temperature. Finally, fluorescence emission at 460 and 535 nm was measured in an EnVision multilabel plate reader (Revvity, Groningen, The Netherlands) after excitation at 400 nm. The ratio of emission at 460 and 535 nm was calculated for each well.

All biological experiments were analyzed using the nonlinear regression curve fitting program with Prism 9 (GraphPad, San Diego, CA). EC_50_, EC_80_, *E*_max_, and IC_50_ values from functional assays were obtained by nonlinear regression analysis. For radioligand binding assays, K_i_ values were determined using the Cheng–Prussoff equation using a K_D_ of 6.3 nM for the radioligand.^25^ All values obtained are the mean ± SEM of at least three separate experiments performed in duplicate, unless stated otherwise.

## Results and Discussion

While the crystal structure of the CCR2 complex with cocrystallized intracellular allosteric ligand (CCR2-RA-[*R*]) was determined previously^45^ (Figure 1A), for CCR5 currently no 3D structure is available, therefore an AlphaFold model^44^ was used to conduct the structure-based tasks in this study. In comparison to CCR2, CCR5 is still understudied, which makes leveraging the AlphaFold model an interesting prospect that may lead to obtaining more selective ligands toward CCR5. In the case of CCR5, **CP2** (first evaluated by Andrews et al.^43^) served as a reference (Figure 1B). The crystal structure of CCR2 was used as a template for refinement of the CCR5 AlphaFold model since the binding pockets only differ in the 6.36 × 36 residue (according to Ballesteros-Weinstein numbering). In particular, Val244 in CCR2 is replaced with Leu236 in CCR5 (cf. Figure 1A and Figure 1B). Other residues within immediate proximity of the ligand are identical in both CCR2 and CCR5 binding sites. Therefore, if a selective ligand can be found, its selectivity will likely depend on interactions with residue 6.36 × 36.

**Figure 1:**
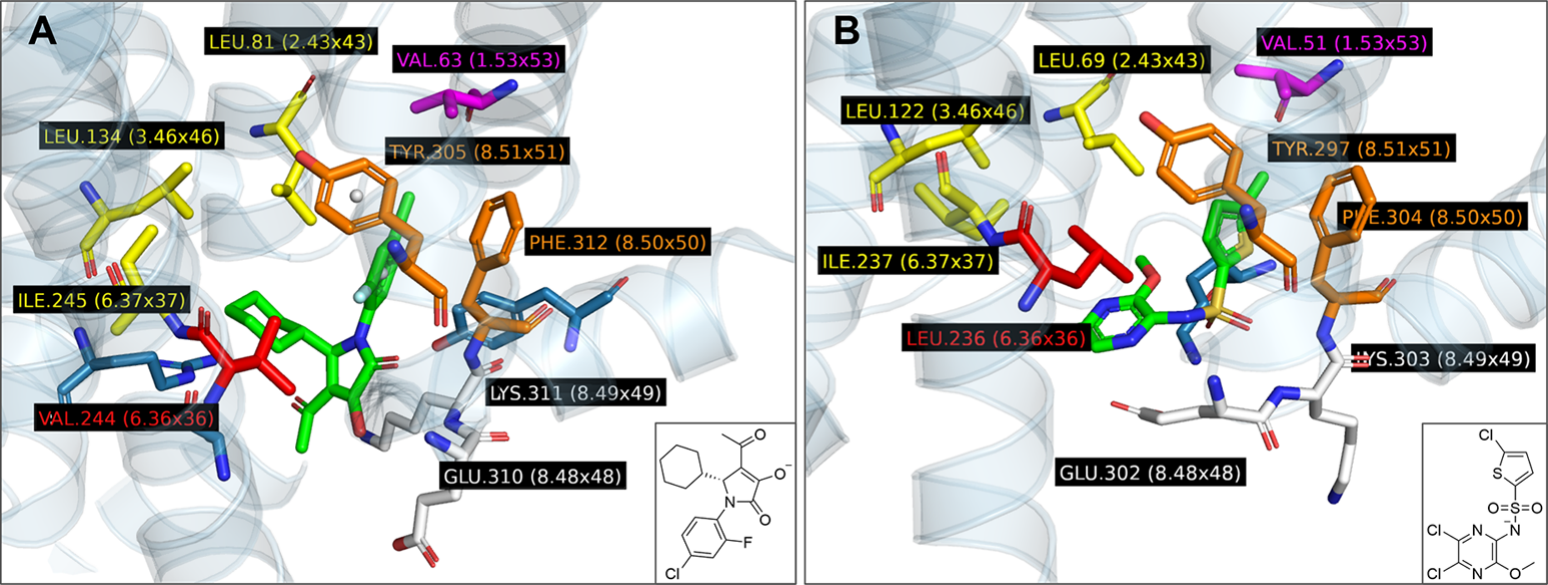
Comparison between binding sites of CCR2 (A) and CCR5 (B). CCR2 is displayed with its cocrystallized CCR2-RA-[*R*] ligand^45^ while CCR5 is shown as snapshot from a stabilized MD simulation trajectory with **CP2** ^43^ (seed 1867 at time 87.90 ns^46^). Relevant residues of both binding pockets are highlighted as follows: Yellow: Lipophilic, Orange: Aromatic, White: hydrogen bond forming, Magenta: halogen bond forming, Blue: Miscellaneous within 5A of each ligand, Red: The key Val244-Leu236 difference between CCR2 and CCR5. Adapted from PLIP^52^ output. The figure was adapted from the raw output of the PLIP^52^ software with default parameters.

In their original study, Andrews et al.^43^ characterized intracellular allosteric binders of CCR4 and CCR5. They identified **CP2** with the pyrazinyl-sulfonamide scaffold (Figure 2) as moderately active on wild type CCR5 (pIC_50_ of 6.1 ± 0.91). However, even at this point, the ChEMBL database still offers only limited data on the activity of pyrazinyl-sulfonamide-containing derivatives on both CCR5 and CCR2 (Figure 2). Therefore, according to the published research, the pyrazinyl-sulfonamide-containing derivatives hold promise, but further exploring their chemical space is warranted (especially due to the small molecular weight of most of the published ligands). In our experiments, we designated **CP2** as the reference compound in docking studies and molecular dynamics simulations, which were used to obtain a refined CCR5 AlphaFold model adjusted for potentially more optimal binding pose of this scaffold.

**Figure 2:**
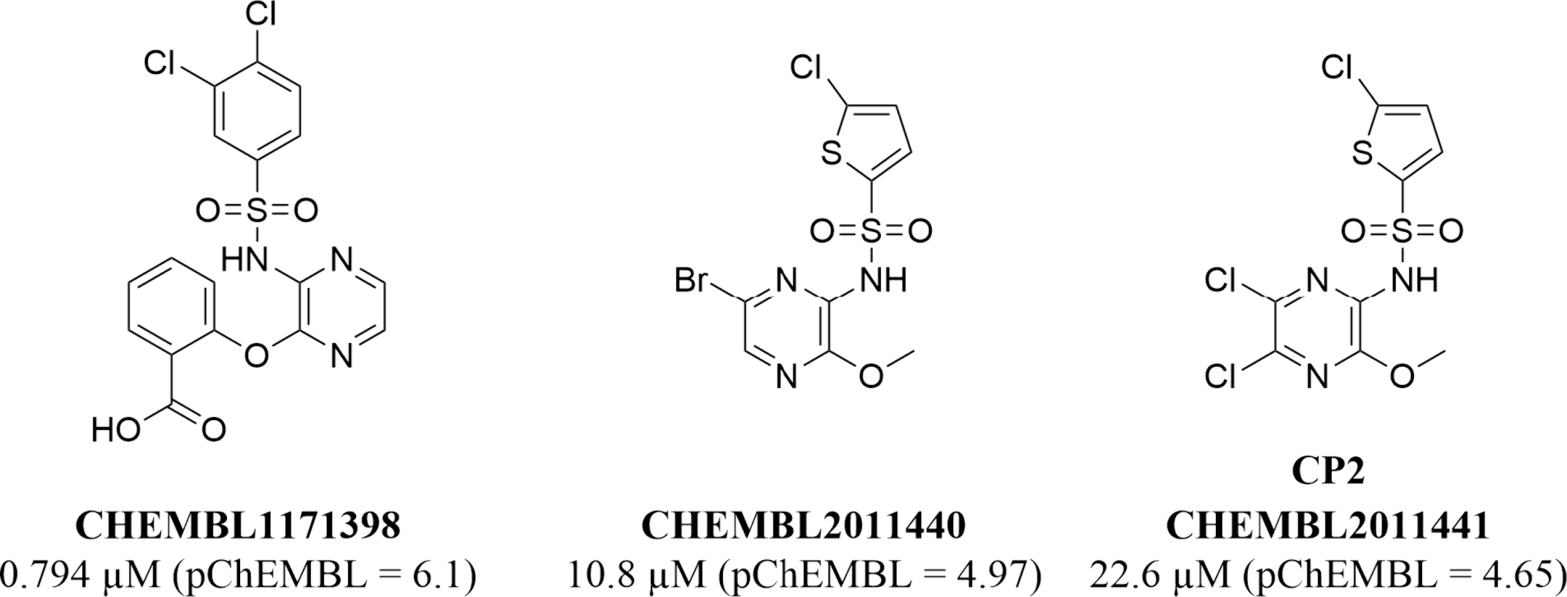
Overview of currently available measurements for compounds with the pyrazinyl-sulfonamide scaffold on CCR2 and CCR5 in the ChEMBL database. Data is sorted left to right in the order of decreasing pChEMBL value. The data was obtained by querying the ChEMBL database with a substructure search equivalent to the following SMARTS string: “[c;R]­S­(=O)­(=O)­Nc1cnccn1”. For compounds with more than one deposited measurement, only the one associated with the highest pChEMBL value is shown. **CP2** is represented as CHEMBL2011441, but the data from the original study by Andrews et al.^43^ is not included since it was not deposited in ChEMBL.

Furthermore, the docking and MD simulations studies with **CP2** in the CCR5 binding pocket revealed that more optimal occupation of the lipophilic subpocket lined with residues Leu69^2.43^, Leu122^3.46^, Ile237^6.37^, and Leu236^6.36^ could be achieved and that the pyrazinyl-sulfonamide scaffold could potentially be expanded in that direction (Figure 1B). This hypothesis was also supported by the position of the CCR2-RA-[*R*] ligand in the CCR2 crystal structure, which shows a similar pose, but with a more optimal occupancy of the lipophilic subpocket (highlighted in yellow in Figure 1A).

The pyrazine sulfonamide part of **CP2** showed close similarities to the binding mode of CCR2-RA-[*R*] in CCR2 as well, which is exemplified by interactions with the aromatic and hydrogen bonding residues (Figure 1B). From the analysis of MD trajectories, we concluded high prevalence of hydrogen bonding with at least two of the conserved residues Glu302^8.48^, Lys303^8.49^, and Phe304^8.50^ in CCR5 (), which are equivalent to Glu310^8.48^, Lys311^8.49^, and Phe312^8.50^ in CCR2. In addition, we observed pi-pi interactions between the aromatic ring system of **CP2** and the Tyr297^7.53^ and Phe304^8.50^ aromatic residues in some of the replicates. Putative halogen bond with Val51^1.53^ was also occasionally present and is likely to contribute to binding. Equivalent interactions to these are also likely to occur in CCR2 based on the pose with a cocrystallized ligand^45^ (Figure 1A) and a previous study.^26^

Most of the MD simulations stabilized at a similar conformation of the protein and orientation of the ligand. Therefore, we decided to extract the protein conformation for the virtual screening step from the last simulation with random seed 1867, which showed more prevalence of interactions with the lipophilic residues of the CCR5 binding site (). The extracted frame was obtained from time 87.90 ns of the simulation after stabilization of the protein–ligand complex (Figure 1B).

To ensure synthetic accessibility of the screening library, we elected to use a combinatorial approach to generate new structures. In this approach a simple amine N-sulfonylation reaction can be used to create the resulting pyrazinyl-sulfonamide scaffold (Figure 3). Therefore, by combining commercial building blocks of differently substituted pyrazinyl-amides with various aromatic sulfonyl-chlorides a relatively large combinatorial library was formed. The building blocks used in this approach were selected from the reactive species annotated in the ZINC15 database^53^ (see Materials and Methods for details). In total, we obtained 200 building blocks to combine in our virtual N-sulfonylation reaction. After exhaustive enumeration of all possible couplings with amine N-sulfonylation we obtained a library of 8,400 possible unique products. This library was further expanded by the generation of all possible tautomeric and protonation states followed by conformer generation, which resulted in the final number of 43,842 chemical species.

**Figure 3:**
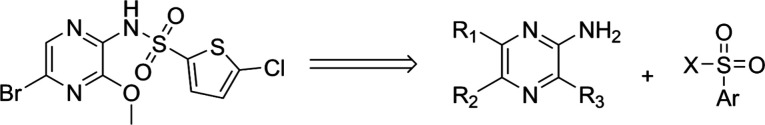
Retrosynthesis analysis of **CP2** into virtual building blocks that were used to form the combinatorial library. In this case, a simple amine N-sulfonylation reaction can be used to combine the identified building blocks into a set of compounds with the pyrazinyl-sulfonamide scaffold with varying substituents R_1_, R_2_, R_3_, Ar, and X (one of −F, −Cl, or −Br).

In the final step of the computational workflow, a virtual screen was conducted on the generated library. Since the size of the library was small, no prefiltering was needed and all 43,842 species were docked into the refined CCR5 AlphaFold model (Figure 1B). The total docking time was less than 43 h utilizing 64 processes at once on a dedicated server with 2x Intel­(R) Xeon­(R) Gold 5220R CPU @ 2.20 GHz and 1TB of system memory (approximately 3 s per compound).

For compound prioritization, we used the calculated docking scores for the best pose of each molecule as the primary criterion. After sorting all docked species on the docking score value, manual filtering and pose assessment of the top molecules was conducted. Important criterium in this manual step was the availability of the building blocks used to synthesize the ligands and structural diversity, but we also looked at important interactions being maintained in the ligand poses (i.e., hydrogen bonds with Glu302^8.48^, Lys303^8.49^ and Phe304^8.50^). This analysis revealed that many top candidates contained similar structural patterns (). We observed that many of the top scoring molecules contained a potential hydrogen bond acceptor adjacent to or fused with the pyrazine ring. Moreover, they contained either methyl or fluorine substituents attached to the benzene ring of the sulfonyl part of the compounds ().

Considering the docking score, and the availability and price of the required building blocks, we followed up compound 26930 (already mentioned as LUF8071 (**9**)). The obtained high-scoring pose showed that morpholine ring could be beneficial since it contains both lipophilic groups as well as an oxygen atom, which may form a novel hydrogen bond interaction with Arg126^3.50^ and Arg140^3.57^ (Figure 4A). However, this hydrogen bonding was not confirmed in subsequent MD simulations (), which showed the morpholine ring more in the vicinity of the lipophilic subpocket (highlighted in yellow in Figure 4B). Therefore, we decided to also include the piperidine variant (LUF8072 (**8**)) and other similar derivatives in the resulting synthesized series (Scheme 1).

**Figure 4:**
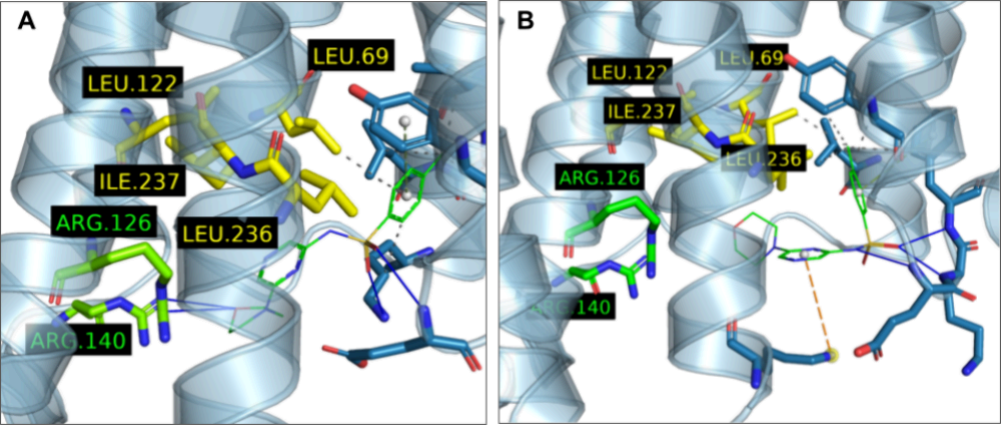
Examples of different poses of LUF8071 (**9**), species 26930 in virtual screening, in (A) the intracellular CCR5 binding pocket obtained from MD experiments after stabilization at frame 40 of simulation with seed 2648 and (B) by docking into the refined AlphaFold structure used during the virtual screening step. Molecular interactions are detected and depicted according to the default PLIP^52^ scheme. The lipophilic subpocket residues are shown in yellow while Arg126^3.50^ and Arg140^3.57^ are shown in green.

**Scheme 1:**
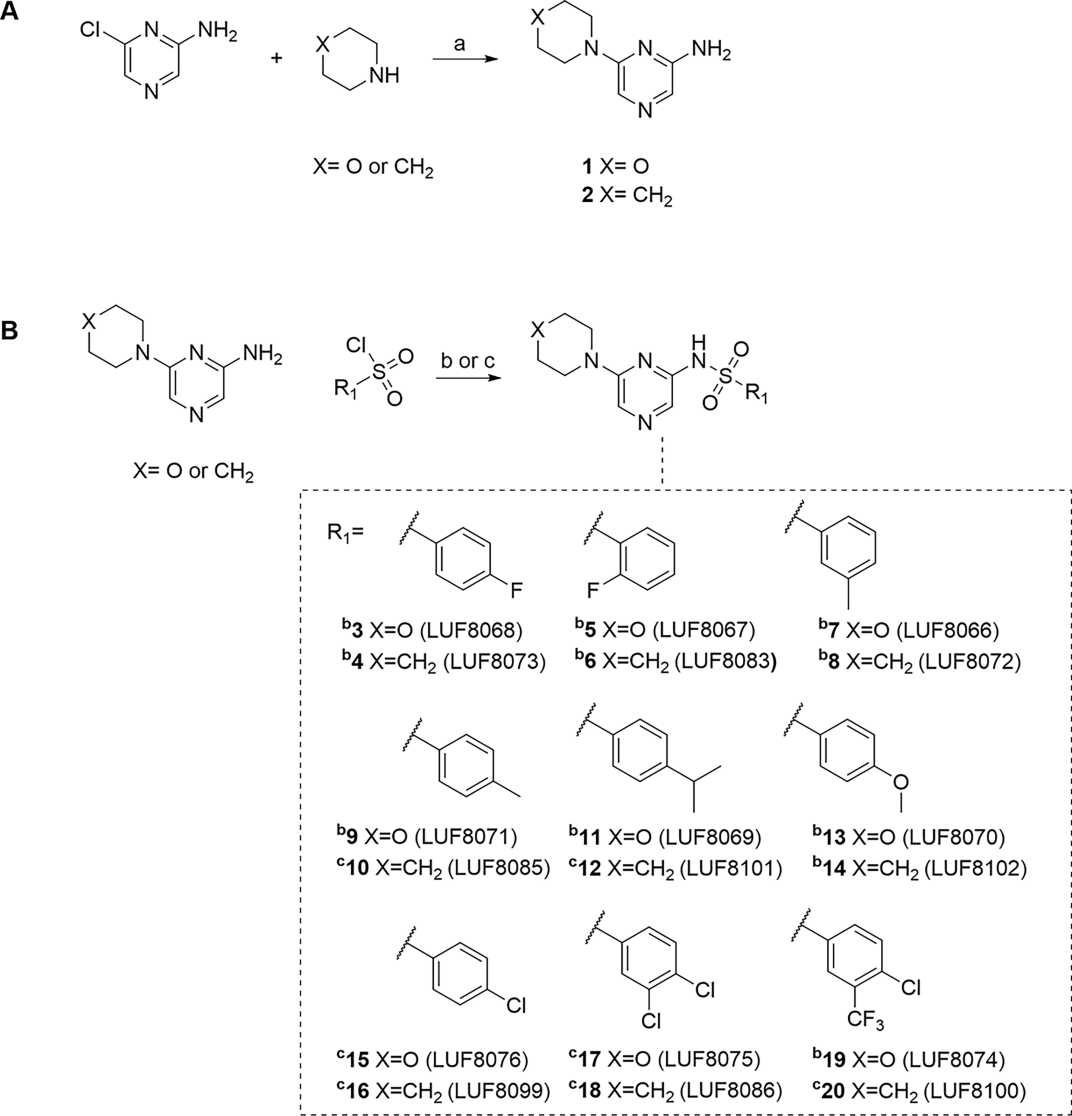
Synthesis of CCR Ligands^a^

a Reagents and conditions: (a) DMAP, pyr., rt, 18 h, 14–60% yield; (b) DMAP, pyr, DCM, pyr, 0 °C-rt, 18 h, 3–41% yield.

Our synthetic strategy aimed toward initially creating the pyrazine scaffold bearing the left-hand-side (LHS) morpholine or piperidine followed by modularly synthesizing various sulfonamides on the right-hand-side (RHS) of the molecule. The pyrazine scaffold synthesis included reacting chloropyrazine amine with morpholine or piperidine at 100 °C under neat conditions to obtain **1** and **2** in moderate to good yields (Scheme 1A).^49^

Various sulfonyl chloride building blocks were selected to form the desired sulfonamides. The synthesized pyrazine scaffolds were employed in a nucleophilic substitution reaction with the corresponding sulfonyl chloride building blocks under basic conditions to obtain **3–20** in low to moderate yields (Scheme 1B).^54^ The reaction conditions were slightly modified based on the reactivity and solubility of the used sulfonyl chloride.

Compounds **3–20** were first evaluated for their effect on the human CCR5 receptor (hCCR5). This was assessed using a β-arrestin-2 recruitment functional assay. U2OS cells expressing hCCR5 were first treated with the known endogenous agonist CCL3 at its EC_80_, followed by treatment with compound **3**-**20** at a single concentration of 10 μM. A cut off point of approximately 50% decrease in CCL3-induced stimulation was deemed sufficient as a hit from the screen. It is noteworthy to mention that certain compounds displayed a slight stimulation of CCR5 higher than the EC_80_ of CCL3 rather than inhibition. However, ligand **20** inhibited β-arrestin-2 recruitment by approximately 54% (Table 1).

**Table 1:**
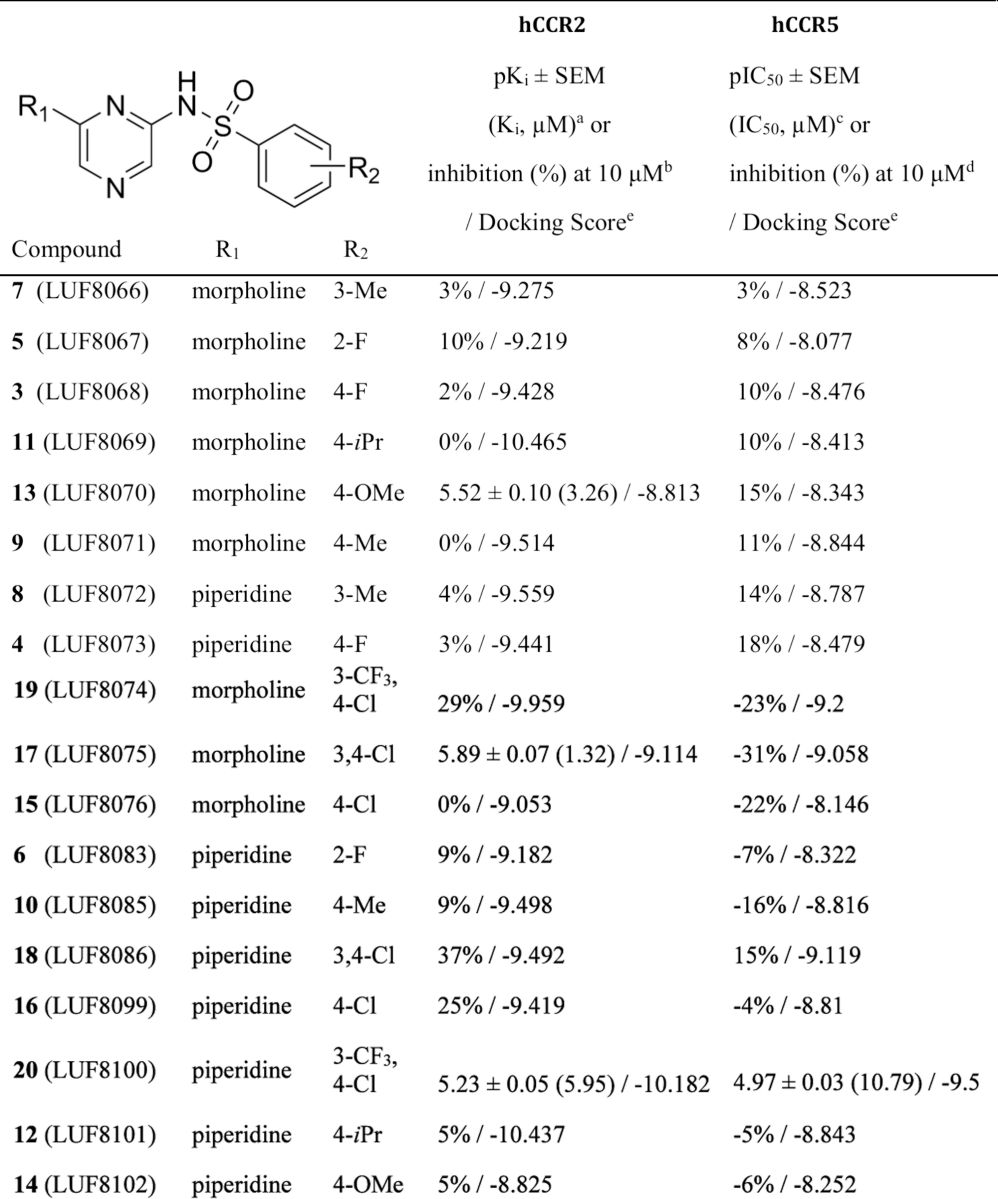
Activities of the Investigated Derivatives of the Virtual Hit LUF8071 (**9**)

a Data are presented as mean p*K*_i_ ± standard error of the mean (SEM) and mean *K*_i_ (nM) of at least three individual experiments performed in duplicate.

b Values represent mean percentage displacement of [^3^H]­CCR2-RA-[*R*] by 10 μM of compound obtained in two individual experiments. Data from individual experiments are shown in brackets.

c Data are presented as mean pIC_50_ ± standard error of the mean (SEM) and mean IC_50_ (nM) of at least three individual experiments performed in duplicate.

d Percent inhibition of β-arrestin recruitment in U2OS cells stably expressing CCR5 by 10 μM compound, in the presence of CCL3 (pEC80 = 7.9). pIC50 values were determined for compounds displaying more than 50% inhibition. % Inhibition values are presented as means values of at least two independent experiments, performed in duplicate.

e Docking score of the top pose generated by AutoDock Vina.

Due to its highest potency on CCR5, LUF8100 (**20**) was further evaluated in the same assay using different concentrations to establish a dose–response curve (Figure 5A). Dose–response analysis revealed that LUF8100 (**20)** possesses an IC_50_ of 10.79 μM. Since the rest of the compounds did not display sufficient inhibition of the CCR5 functional response, no clear structure activity relationship could be deduced for these ligands. However, the combination of meta CF_3_ and para chloro moieties has been observed as a desirable substituent in previously discovered CCR2 allosteric ligands,^14,55^ which is in line with LUF8100 (**20)** combining these features as the most potent compound.

**Figure 5:**
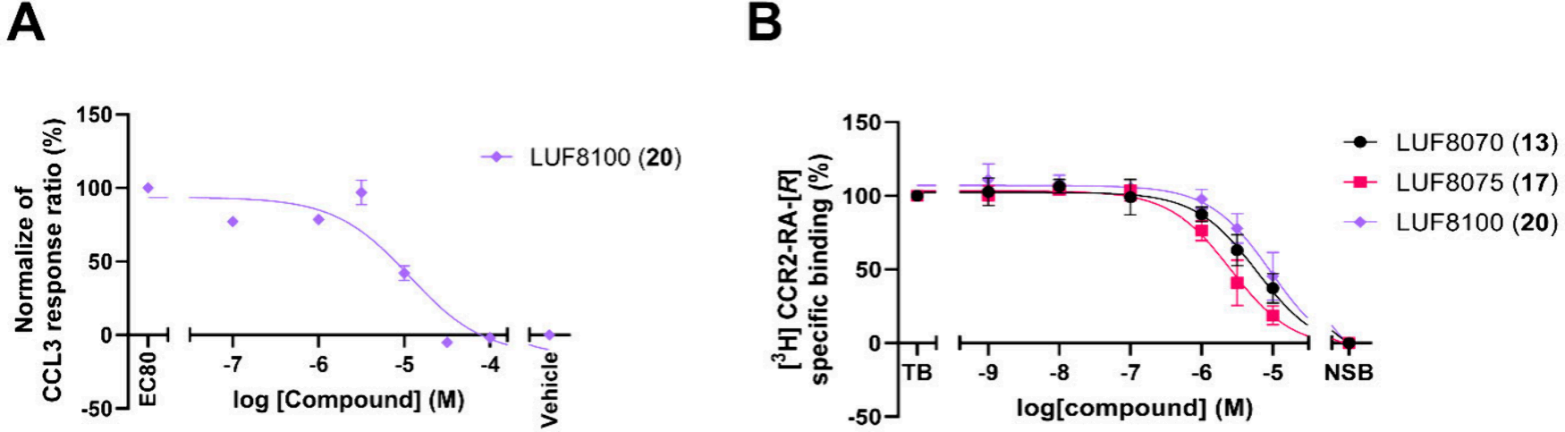
(A) Dose–response curve of LUF8100 (**20**) in a β-arrestin recruitment assay on U2OS-hCCR5. (B) Displacement curve of [^3^H] CCR2-RA-[*R*] by LUF8070 (**13**), LUF8075 (**17**), and LUF8100 (**20**) on U2OS-hCCR2.

CCR2 exhibits high homology to CCR5 (>76% overall homology,^56^ and only one residue difference, Val244 in CCR2 vs Leu236 in CCR5, in the putative intracellular binding pocket). Therefore, we were interested in investigating if the ligands discovered during the virtual screen could exhibit dual or selective effects on CCR5 and CCR2. Thus, the binding affinity of all compounds was also evaluated using a radioligand displacement assay (Table 1). [^3^H]-CCR2-RA-[*R*] was used as the radiolabeled ligand of choice as previously described.^25^ The synthesized compounds were initially tested at a single point concentration of 10 μM. Ligands displaying more than 50% displacement of [^3^H]-CCR2-RA-[*R*] were deemed sufficient for further evaluation. Compounds LUF8070 (**13)** and LUF8075 (**17**), and LUF8100 (**20**) fulfilled the mentioned displacement threshold (Table 1). The rest of the compounds displayed either low or no displacement of [^3^H]-CCR2-RA-[*R*]. Further testing of LUF8070 (**13**) and LUF8075 (**17**), and LUF8100 (**20**) at various concentrations provided a dose–response curve with a K_i_ of 3.26, 1.32, and 5.95 μM, respectively (Table 1, Figure 5B).

The obtained experimental results for both CCR2 and CCR5 show that even small structural modifications on the RHS and LHS of the molecule can contribute to potency significantly and we decided to explore these trends further with molecular docking into the stabilized conformation of CCR5 obtained from MD experiments with LUF8071 (**9**) (Figure 4B).

In terms of dockings scores, we observed that for the case of CCR5 the most active compound, LUF8100 **(20)**, also achieved the best docking score of −9.5 (Table 1). However, this was not true for CCR2 where many clearly inactive compounds (i.e., LUF8071 (**9**), LUF8072 (**8**) or LUF8069 (**11**)) obtained better docking scores than the confirmed binders (LUF8070 (**13**), LUF8075 (**17**), and LUF8100 (**20)**). Therefore, our results also reflect the fact that docking scores and experimentally confirmed activity are generally not correlated in virtual screening campaigns.

It is clear from the experimental results that the replacement of morpholine for piperidine and the introduction of −CF_3_ and −Cl groups to the phenyl ring in positions 3 and 4 in LUF8100 (**20)** led to about 30% improvement in activity on CCR5 when compared to LUF8071 (**9)** (Table 1). The introduction of the more lipophilic piperidine ring likely meant improved potential for the occupation of the lipophilic subpocket (Figure 6A) lined with Leu69^2.43^, Leu122^3.46^, Ile237^6.37^ and Leu236^6.36^. However, a hydrophobic interaction with Thr65^2.39^ can also be observed in the docked pose (Figure 6A). On the other side of the pocket, the bulky −CF_3_ and -Cl substituents likely fill the same subpocket that the -Me group of LUF8071 (**9**) was shown to occupy in the initial computational experiments (c.f. Figure 4 and Figure 6A), but with one of the fluorines in LUF8100 also optimally positioned to form a halogen bond with Val51^1.53^. It should also be noted that we did not observe optimal pi-stacking configuration in LUF8100, unlike for example in the case of LUF8071 (Figure 6B) in CCR2. However, we attribute this to the fact that the CCR2 binding site model comes from an experimentally determined crystal structure with optimal configuration and not from an MD trajectory.

**Figure 6:**
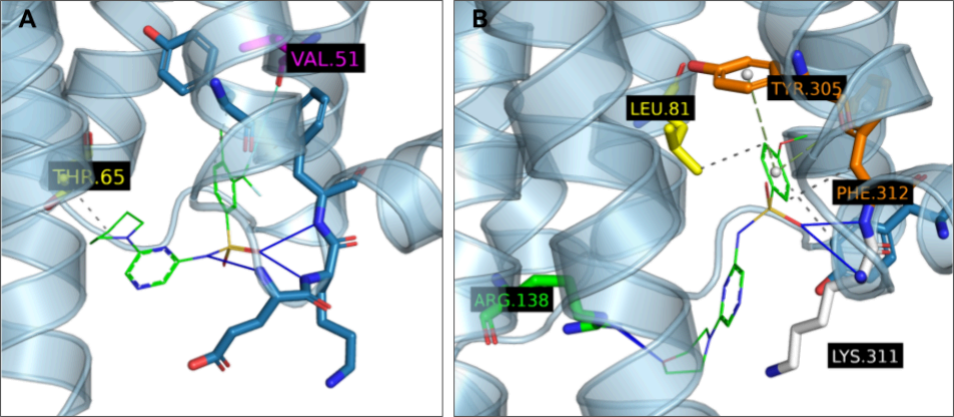
(A) Optimal docked pose of LUF8100 (**20**) in the refined CCR5 structure obtained from a snapshot of the most stable MD trajectory with LUF8071 (**9**) and (B) LUF8070 (13) docked in the CCR2 PDB structure obtained via cocrystallization with CCR2-RA-[R].^45^ Molecular interactions are detected and depicted according to the default PLIP^52^ scheme. The lipophilic subpocket likely interacting with the piperidine ring is shown in yellow while the assumed halogen-binding Val51^1.53^ is shown in magenta.

Interestingly, the RHS and LHS substitutions seem to have a synergistic effect since separate introductions of these modifications in LUF8074 (**19**) and LUF8085 (**10**) did not lead to any improved antagonist activity on CCR5 (Table 1). Perhaps affixing the benzenesulfonyl part of the ligand in place with improved halogen bonding with Val51^1.53^ and the overall increase in size both make it more likely for the piperidine ring to interact with the lipophilic side chains of residues Leu69^2.43^, Leu122^3.46^, Ile237^6.37^, Leu236^6.36^ and Thr65^2.39^ further improving the overall affinity. It is also likely that the increased bulk resulting from these substitutions contributes to binding by increasing the van der Waals effect across the cavity, which could mean further increase in binding affinity. This is consistent with previous findings for other compound series,^14,25,55^ which showed that the presence of −CF_3_ and halogen groups on the phenyl ring and strategically placed bulkier lipophilic groups^14^ can improve bioactivity. In addition, it can be observed that even small changes to the groups on the phenyl core can have profound effect on activity as exemplified in the stark contrast in activity between LUF8100 (**20**) and LUF8099 (**16**), which might suggest particular sensitivity to the mutual positions of the piperidine ring and the phenyl ring substituents in the suggested compounds.

The synergistic effect between substitutions on the RHS and LHS of the synthesized molecules is also supported by other docking experiments conducted on the series. For example, we can see that in the poses generated for LUF8074 and LUF8099 (Figure 7A and Figure 7B, respectively) some interactions are missing in comparison to those observed for LUF8100 (Figure 6A). In particular, LUF8074 is lacking the lipophilic interaction with Thr65 and LUF8099 the halogen bond with Val51. Therefore, it is possible that both interactions in LUF8100 and the increased bulk help to stabilize the complex and may also play an important role in inducing the appropriate pocket conformation to form the more persistent interactions observed (i.e., hydrogen bonds and pi-stacking).

**Figure 7:**
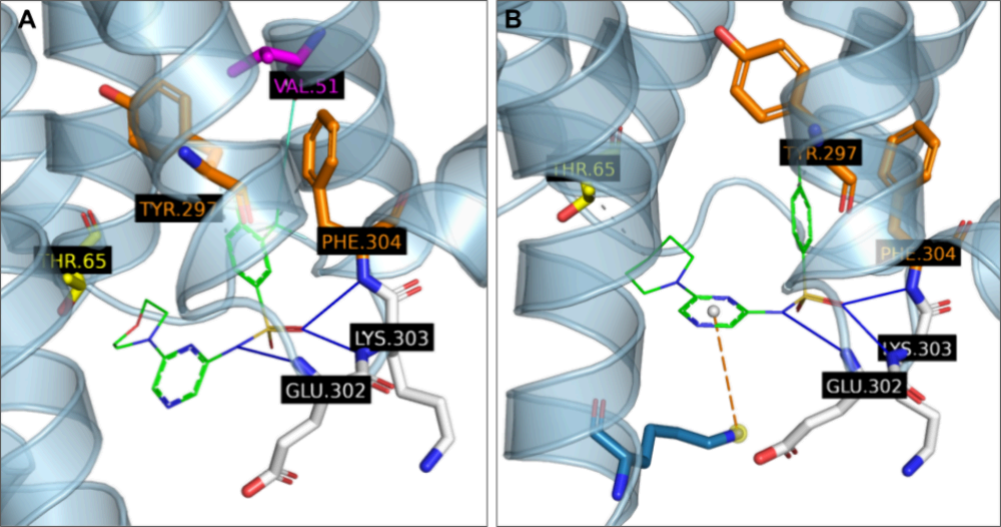
Comparison of poses generated for (A) LUF8074 and (B) LUF8099 inside the model of the CCR5 intracellular cavity (obtained from a snapshot of the most stable MD trajectory with LUF8071). Molecular interactions are detected and depicted according to the default PLIP^52^ scheme.

CCR2 has the most similar intracellular allosteric binding pocket to CCR5 with Leu236^6.36^ replaced by Val244^6.36^ (Figure 1), which would suggest similar trends should be observed in binding between CCR2 and CCR5. Interestingly, in the experimental evaluation, two of the morpholine derivatives (LUF8070 (**13**) and LUF8075 (**17**)) and one piperidine derivative (LUF8100 (**20**)) showed large enough displacement of a known intracellular allosteric ligand (CCR2-RA-[*R*]) in CCR2 (Table 1). In the CCR2 binding assay (Table 1), LUF8100 (**20**) (also the most active compound in the CCR5 β-arrestin recruitment assay) showed slightly lower affinity when compared to LUF8070 (**13**) and LUF8075 (**17**). Surprisingly, these two compounds were deemed inactive in the CCR5 β-arrestin recruitment assay. Given the close similarity of the CCR2 and CCR5 binding pockets one would expect the CCR5 β-arrestin recruitment assay to be more in line with the CCR2 binding assay for LUF8070 (**13**) and LUF8075 (**17**), but other factors must have been at play. For example, it may not have been low affinity to CCR5 that caused the inactivity of these compounds in the β-arrestin recruitment assay, but rather differences in cell permeability (i.e., β-arrestin recruitment assay is performed using whole cells, while the radioligand displacement assay uses cell membranes). Therefore, we cannot rationalize these effects sufficiently from pure binding affinity perspective, but development of a CCR5 specific radioligand would enable us to further investigate these differences more reliably in the future.

Also noteworthy is the fact that LUF8070 (**13**) introduces a simple modification by replacing the 4-Me group of the initial virtual screening hit LUF8071 (**9**) by 4-OMe, which causes a significant increase in CCR2-RA-[*R*] displacement in LUF8070 (**13**) (Table 1). This introduction of 4-OMe may improve binding to CCR2 by providing more rotational flexibility to the methyl group, which may be more easily accommodated by the lipophilic residues Leu67^1.57^, Ile66^1.56^ and Val63^1.53^ on the RHS of the CCR2 pocket. Similarly, LUF8075 (**17**) with 3,4-Cl substituents also showed increased binding, further confirming that introduction of halogen substituents to the phenyl ring is beneficial also in CCR2 with a likely halogen bonding interaction with Val63^1.53^. However, it is important to note that we did not strictly observe these interactions in the generated poses for LUF8070 (**13**), LUF8075 (**17**) or LUF8100 (**20**) in the binding pocket of CCR2 (Figure 6B, Figure 8A and Figure 8B, respectively). This is likely due to the fact that this series of ligands induces a slightly different conformation of the binding pocket than CCR2-RA-[*R*]. The docked poses of LUF8070 (**13**) and LUF8075 (**17**) also show potential for a hydrogen bond interaction with Arg138^3.50^, which is the equivalent of Arg126^3.50^ in CCR5. It is possible that this residue is more accessible in CCR2 than CCR5, which may be the reason why in CCR2 these compounds were more successful. However, more investigation is needed in this direction and the ability to correlate binding affinity data for CCR2 and CCR5 with MD simulations would help to elucidate possible selectivity patterns between those two highly homologous proteins. For both proteins, the generated poses and their depictions using the PLIP software to highlight putative interactions are archived on Zenodo for further analysis.^57^ In terms of novelty, the discovered scaffolds of the most potent compounds LUF8070 (**13**), LUF8075 (**17**), and LUF8100 (**20**) on either CCR2 or CCR5 are structurally distinct from already reported compounds (Figure 2). In fact, the overall most similar synthesized compound to any of the previously published compounds (Figure 2) was LUF8086 (**18**), which had Tanimoto similarity of 0.25 (Morgan fingerprint with 1024 bits and radius 2) when compared to CHEMBL2011440.

**Figure 8:**
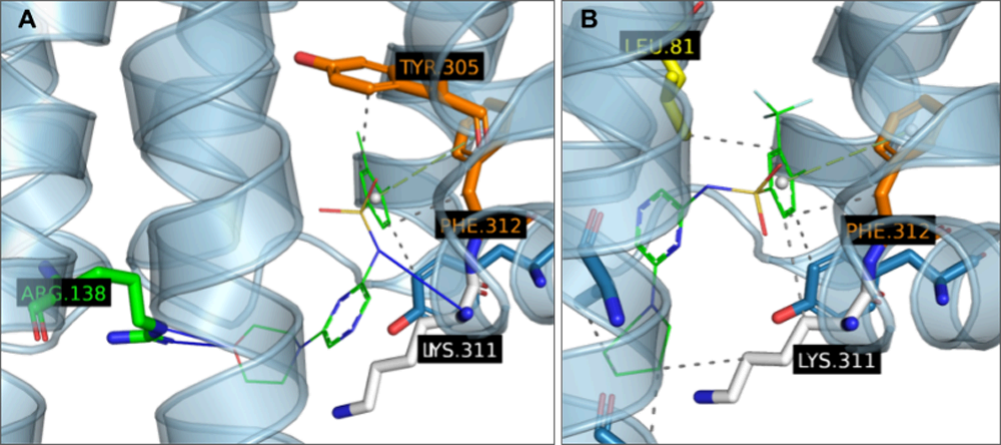
Poses generated by AutoDock Vina for (A) LUF8075 and (B) LUF8100 in the CCR2 intracellular binding pocket as determined from the crystal structure with CCR2-RA-[*R*].^45^ Molecular interactions are detected and depicted according to the default PLIP^52^ scheme.

## Conclusion

In this work a small-scale virtual screening campaign was conducted to discover novel CCR2 and CCR5 intracellular allosteric ligands. Only 1 out of 18 (5%) compounds displayed significant inhibition of CCR5 and 3 out of 18 (15%) of the synthesized ligands showed moderate affinity for CCR2. These numbers are comparable to the hit rate obtained from many large-scale high throughput screening studies where it is often significantly lower than 5%.^40^ In addition, we demonstrated that AlphaFold models can be useful tools for virtual screening and structure-based design. However, preliminary analysis of the protein structures needs to be conducted when using them and adjustments to the residues might be needed. This work also proves that the design of selective ligands for closely related proteins is challenging and that the development of a radioligand for CCR5 could be instrumental in understanding the differences in binding of the developed molecules to CCR2 and CCR5 in the future. Access to such data would be valuable to assess the role of the Val244-Leu236 mutation with more advanced modeling techniques such as free energy perturbation. This would provide valuable insights into the relative binding of the proposed ligands, which would in turn advance the development of selective modulators toward either CCR2 or CCR5.

## Supplementary Material









## Data Availability

The code and all input and output files needed to reproduce all virtual screening steps from library generation to molecular docking are deposited on Zenodo with documentation and license information included. Likewise for molecular dynamics analysis, all configuration, input, output and project files are also provided in this associated data package. The complete compressed data package with a README file documenting its contents can be accessed from the following link: 10.5281/zenodo.13736590.

## Notes

Supporting figures and tables for this manuscript (PDF)
Detailed methodology of organic synthesis (PDF)
Analytical spectra of all prepared analogues (PDF)
Data from Table 1 in machine readable format, including SMILES strings of each structure (CSV)

## Abbreviations

CCR2: CC chemokine receptor 2
CCL2: CC chemokine ligand 2
CCR5: CC chemokine receptor 5
TLC: thin layer chromatography
