# Supplementary material for: Combining AlphaFold with Focused Virtual Library Design in the Development of Novel CCR2 and CCR5 Antagonists
Source: J Chem Inf Model. 2025 Nov 12;65(22):12398–409. doi: 10.1021/acs.jcim.5c01596 (PMC12648652; doi:10.1021/acs.jcim.5c01596)
Supplement: Supplementary file 1 [file ci5c01596_si_001.pdf]

Combining AlphaFold with Focused Virtual Library Design in the Development of Novel CCR2 and CCR5 Antagonists: Supplementary Information

Khaled Essa[1,2], Kian Noorman van der Dussen [1], Yao Yao [1], Bente Bleijs[1], Natalia Ortiz Zacarias [1,2], Laura H. Heitman [1,2], Gerard van Westen [1], Willem Jespers[3], Daan van der Es [1], Martin Šícho[1,4]*.*

[1]Division of Medicinal Chemistry, Leiden Academic Centre for Drug Research (LACDR), Leiden University, 2333 CC Leiden, The Netherlands.

[2]Oncode Institute, 2333 CC Leiden, The Netherlands

[3]Department of Medicinal Chemistry, Photopharmacology and Imaging, Groningen, Research Institute of Pharmacy (GRIP), Faculty of Science and Engineering, Antonius Deusinglaan 1, 9713 AV Groningen, The Netherlands

[4]CZ-OPENSCREEN: National Infrastructure for Chemical Biology, Department of Informatics and Chemistry, Faculty of Chemical Technology, University of Chemistry and Technology Prague, Technická 5, 166 28, Prague, Czech Republic

Corresponding Authors

*Martin Šícho (martin.sicho@vscht.cz), *Daan van der Es (d.van.der.es@lacdr.leidenuniv.nl)

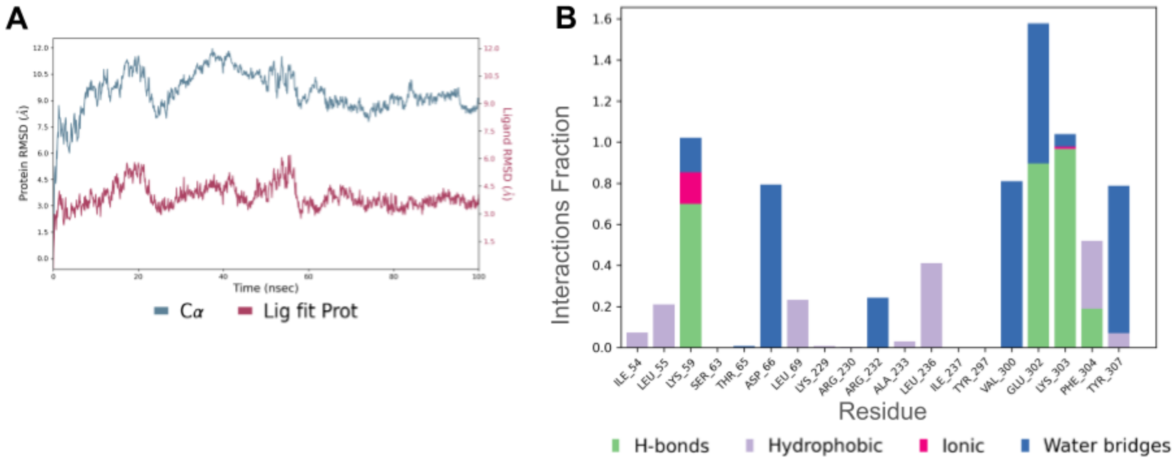

Figure S1: First out of 3 molecular dynamics experiments performed with compound 2 docked into the manually refined AlphaFold model of CCR5: (A) RMSD of both protein and ligand in comparison to the initial pose and (B) the histogram of recorded interactions. All three experiments were performed from the same initial pose obtained with AutoDock Vina.

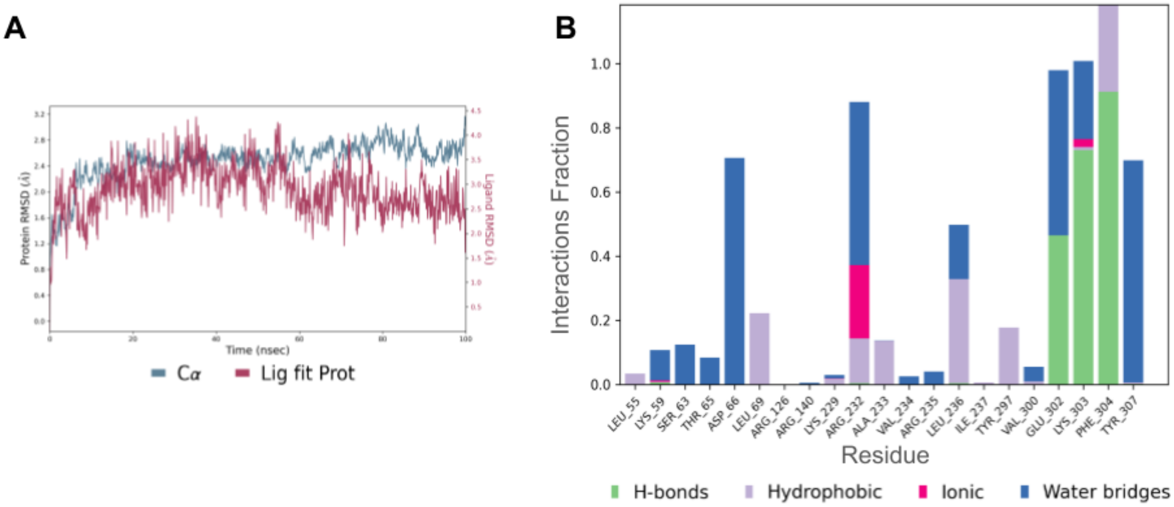

Figure S2: First out of 3 molecular dynamics experiments performed with initial virtual screening hit 26930 (LUF 8071) docked into the manually refined AlphaFold model of CCR5. : (A) RMSD of both protein and ligand in comparison to the initial pose and (B) the histogram of recorded interactions. All three experiments were performed from the same initial pose obtained with AutoDock Vina.

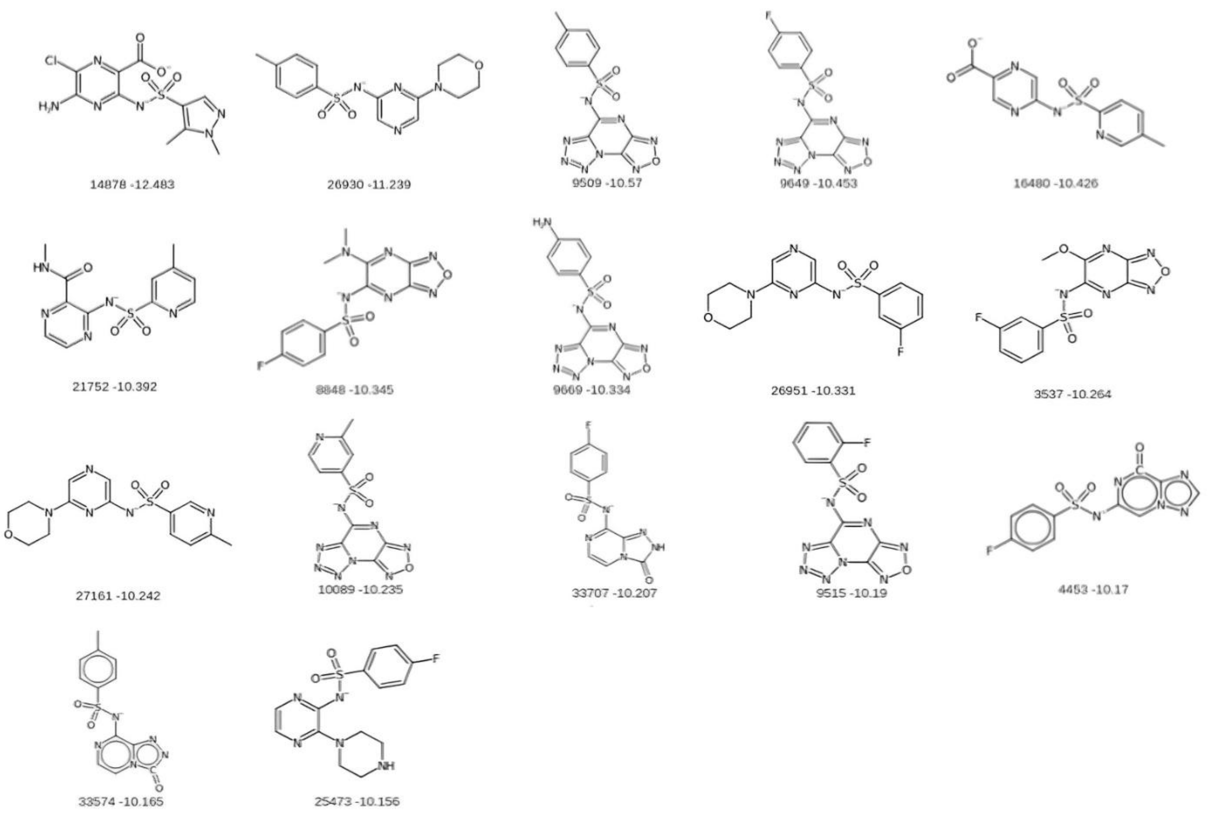

Figure S3: Structures, unique identifiers and docking scores of top scoring molecules in the virtual screening campaign.
