# Supplementary material for: Combining AlphaFold with Focused Virtual Library Design in the Development of Novel CCR2 and CCR5 Antagonists
Source: J Chem Inf Model. 2025 Nov 12;65(22):12398–409. doi: 10.1021/acs.jcim.5c01596 (PMC12648652; doi:10.1021/acs.jcim.5c01596)
Supplement: Supplementary file 2 [file ci5c01596_si_002.pdf]

Combining AlphaFold with Focused Virtual Library Design in the Development of Novel CCR2 and CCR5 Antagonists: Supplementary Information

Khaled Essa[1,2], Kian Noorman van der Dussen [1], Yao Yao [1], Bente Bleijs[1], Natalia Ortiz Zacarias [1,2], Laura H. Heitman [1,2], Gerard van Westen [1], Willem Jespers[3], Daan van der Es [1], Martin Šícho[1,4]*.*

[1]Division of Medicinal Chemistry, Leiden Academic Centre for Drug Research (LACDR), Leiden University, 2333 CC Leiden, The Netherlands.

[2]Oncode Institute, 2333 CC Leiden, The Netherlands

[3]Department of Medicinal Chemistry, Photopharmacology and Imaging, Groningen, Research Institute of Pharmacy (GRIP), Faculty of Science and Engineering, Antonius Deusinglaan 1, 9713 AV Groningen, The Netherlands

[4]CZ-OPENSCREEN: National Infrastructure for Chemical Biology, Department of Informatics and Chemistry, Faculty of Chemical Technology, University of Chemistry and Technology Prague, Technická 5, 166 28, Prague, Czech Republic

Corresponding Authors

*Martin Šícho (martin.sicho@vscht.cz), *Daan van der Es (d.van.der.es@lacdr.leidenuniv.nl)

Chemistry

General procedure 1 (GP1)

In a 10 ml flask, 6-chloropyrazin-2-amine (1 equiv.) was added followed by corresponding amine (2 equiv.). The reaction mixture was stirred at 100 °C for 18h. Upon reaction completion, the mixture was diluted with water and extracted with EtOAc. The organic layer was dried over $MgSO_4$ then concentrated under reduced pressure.The crude mixture was purified by flash column chromatography using a gradient elution of 70-100% EtOAc/petroleum ether.

General procedure 2 (GP2)

A 10 ml flask was charged with the corresponding pyrazine-amine scaffold (1 equiv.) and DMAP (0.1-0.2 equiv.) dissolved in Pyridine (0.05M).The corresponding sulfonyl chloride (1.2-2 equiv.) dissolved in Pyridine (0.05M) was added dropwise. The reaction was stirred at rt for 18h. Upon reaction completion, pyridine was evaporated under reduced pressure and the residue was taken up in EtOAc and washed three times with 1M HCl. The organic layer was dried over $MgSO_4$ then concentrated under reduced pressure. The crude mixture was purified by flash column chromatography using a gradient elution of 3-6% MeOH/DCM.

General procedure 3 (GP3)

A 10 ml flask was charged with 6-(piperidin-1-yl)pyrazin-2-amine (1 equiv.) dissolved in pyridine (2 equiv.) and DCM (0.05M) and cooled to 0°C. The corresponding sulfonyl chloride (2 equiv.) dissolved in DCM (0.05 M) was added dropwise to amine mixture under inert conditions. The reaction mixture was allowed to heat to rt and stirred for 18h. Upon reaction completion, the reaction mixture was diluted in EtOAc and washed twice with 1M HCl. The aqueous layer was back extracted thrice with EtOAc. The combined organic layers were dried over $MgSO_4$, filtered and evaporated under reduced pressure. The crude mixture was purified by flash column chromatography using a gradient elution of 2-6% MeOH/DCM.

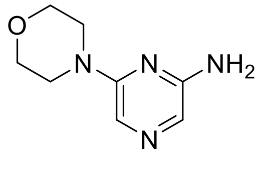

6-morpholinopyrazin-2-amine (1)

6-chloropyrazin-2-amine (2.59 g, 20 mmol) and morpholine (3.48g, 40 mmol) were reacted according to **GP1** to obtain 6-morpholinopyrazin-2-amine (2.750 g, 15.26 mmol, 76 % yield) as a yellow solid. LC-MS (ESI) m/z $[M + H]^+$ calcd for $C_8H_{12}N_4O$ $[M + H]^+$ 181.10, found 181.00. ^1H NMR (400 MHz, CDCl3) δ 7.47 (s, 1H), 7.36 (s, 1H), 4.29 (s, 2H), 3.84 – 3.72 (m, 5H), 3.53 – 3.45 (m, 5H).

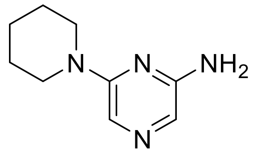

6-(piperidin-1-yl)pyrazin-2-amine (2)

6-chloropyrazin-2-amine (1.943g, 15 mmol) and piperidine (2.96 ml, 30.0 mmol) were reacted according to **GP1** to 6-(piperidin-1-yl)pyrazin-2-amine (1.937 g, 10.87 mmol, 72 % yield) as a yellow solid. LC-MS (ESI) m/z calcd for $C_9H_{14}N_4$ [M + H]$^+$ 179.12, found 179.16. ^{1}H NMR (400 MHz, DMSO) δ7.32 (s, 1H), 7.10 (s, 1H), 5.89 (s, 2H), 3.49 – 3.38 (m, 5H), 1.63 – 1.54 (m, 2H), 1.54 – 1.42 (m, 5H). ^{13}C NMR (101 MHz, DMSO) δ 154.3, 154.0, 119.2, 117.1, 45.4, 25.5, 24.8.

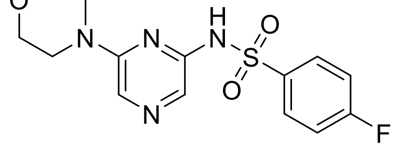

4-fluoro-N-(6-morpholinopyrazin-2-yl)benzenesulfonamide (3)

6-morpholinopyrazin-2-amine (50.0 mg, 0.277 mmol), DMAP (6.78 mg, 0.055 mmol), and 4-fluorobenzenesulfonyl chloride (81 mg, 0.416 mmol) were reacted according to **GP2** to obtain 4-fluoro-N-(6-morpholinopyrazin-2-yl)benzenesulfonamide (56 mg, 0.166 mmol, 60 % yield) as an off white solid. LC-MS (ES) m/z calcd for $C_{14}H_{15}FN_4O_3S$ [M + H]$^+$ 339.08, found 339.05. ^{1}H NMR (400 MHz, DMSO) δ 11.24 (br s, 1H), 8.01 – 7.91 (m, 2H), 7.83 (s, 1H), 7.51 (s, 1H), 7.49 – 7.38 (m, 2H), 3.60 (t, J = 4.6 Hz, 4H), 3.37 – 3.33 (m, 4H). ^{13}C NMR (101 MHz, DMSO) δ 165.6, 163.1, 152.5, 145.5, 136.9, 130.1, 130.0, 124.2, 120.6, 116.5, 116.2, 65.6, 44.1.

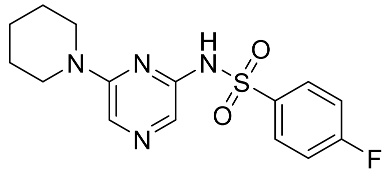

4-fluoro-N-(6-(piperidin-1-yl)pyrazin-2-yl)benzenesulfonamide (4)

6-(piperidin-1-yl)pyrazin-2-amine (50.0 mg, 0.281 mmol), DMAP (6.85 mg, 0.056 mmol), and 4-fluorobenzenesulfonyl chloride (109 mg, 0.561 mmol) were reacted according to **GP2** to obtain 4-fluoro-N-(6-(piperidin-1-yl)pyrazin-2-yl)benzenesulfonamide (47 mg, 0.140 mmol, 50% yield) as a grey solid. LC-MS (ESI) m/z calcd for $C_{15}H_{17}FN_4O_2S$ [M + H]$^+$ 337.10, found 337.10. ^{1}H NMR (400 MHz, DMSO) δ 11.14 (s, 1H), 7.95 (dd, J = 8.7, 5.2 Hz, 2H), 7.81 (s, 1H), 7.44 (t, J = 8.8 Hz, 2H), 7.41 (s, 1H), 3.38 (t, J = 5.4 Hz, 4H), 1.62 – 1.47 (m, 2H), 1.47 – 1.31 (m, 4H). 13C NMR (101 MHz, DMSO) δ ^{13}C NMR (101 MHz, DMSO) δ 165.5, 163.0, 152.4, 145.6, 137.2, 130.0, 129.9, 124.2, 119.3, 116.5, 116.2, 44.8, 24.7, 24.1.

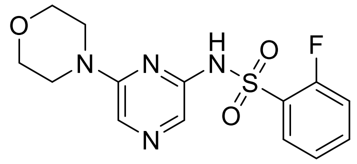

2-fluoro-N-(6-morpholinopyrazin-2-yl)benzenesulfonamide (5)

6-morpholinopyrazin-2-amine (50.0 mg, 0.277 mmol), DMAP (3.39 mg, 0.028 mmol) and 2-fluorobenzenesulfonyl chloride (0.055 ml, 0.416 mmol) were reacted according to **GP2** to obtain 2-fluoro-N-(6-morpholinopyrazin-2-yl)benzenesulfonamide (27 mg, 0.080 mmol, 29 % yield) as a yellow solid. LC-MS (ESI) m/z calcd for $C_{14}H_{15}FN_4O_3S$ $[M + H]^+$ 339.08, found 339.00. ^1H NMR (400 MHz, CDCl3) δ 8.02 (td, J = 7.5, 1.8 Hz, 1H), 7.79 (s, 2H), 7.64 – 7.52 (m, 1H), 7.30 (dd, J = 7.8, 1.1 Hz, 1H), 7.18 (ddd, J = 9.7, 8.4, 1.1 Hz, 1H), 3.75 (t, J = 4.8 Hz, 4H), 3.43 (t, J = 5.1 Hz, 4H). ^{13}C NMR (101 MHz, CDCl3) δ 160.6, 158.0, 153.6, 144.9, 136.2, 131.5, 125.7, 124.8, 121.3, 117.7, 66.7, 44.8.

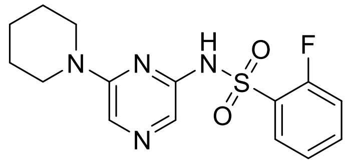

2-fluoro-N- (6-(piperidin-1-yl)pyrazin-2-yl)benzenesulfonamide (6)

6-(piperidin-1-yl)pyrazin-2-amine (50.0 mg, 0.281 mmol), DMAP (6.85 mg, 0.056 mmol), and 2-fluorobenzenesulfonyl chloride (0.056 ml, 0.421 mmol) were reacted according to **GP2** to obtain 2-fluoro-N-(6-(piperidin-1-yl)pyrazin-2-yl)benzenesulfonamide. LC-MS (ESI) m/z calcd for $C_{15}H_{17}FN_4O_2S$ $[M + H]^+$ 337.10, found 337.10. ^1H NMR (400 MHz, DMSO) δ 11.44 (s, 1H), 7.97 (td, J = 7.6, 1.8 Hz, 1H), 7.80 (s, 1H), 7.71 (dddd, J = 8.0, 6.8, 5.0, 1.8 Hz, 1H), 7.47 – 7.35 (m, 3H), 3.29 (t, J = 5.3 Hz, 4H), 1.59 – 1.44 (m, 2H), 1.43 – 1.23 (m, 4H). 13C NMR (101 MHz, DMSO) δ 159.3, 156.8, 152.3, 145.6, 135.7, 135.7, 130.7, 128.5, 128.4, 124.8, 124.7, 124.2, 119.2, 117.3, 117.1, 44.7, 24.7, 24.0.

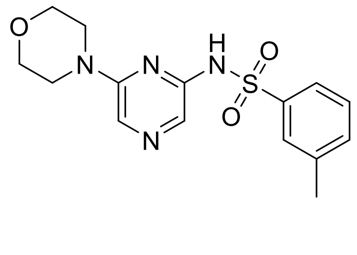

3-methyl-N-(6-morpholinopyrazin-2-yl)benzenesulfonamide (7)

6-morpholinopyrazin-2-amine (30.0 mg, 0.166 mmol), DMAP (2.034 mg, 0.017 mmol) and 3-methylbenzenesulfonyl chloride (0.029 ml, 0.200 mmol) were reacted according to **GP2** to obtain 3-methyl-N-(6-morpholinopyrazin-2-yl)benzenesulfonamide (15 mg, 0.045 mmol, 27 % yield) as a yellow

solid. LC-MS (ESI) m/z calcd for $C_{15}H_{18}N_4O_3S$ $[M + H]^+$ 335.11, found 335.10. ^{1}H NMR (400 MHz, CDCl3) δ 7.98 (s br, 1H), 7.83 – 7.72 (m, 4H), 7.47 – 7.37 (m, 2H), 3.75 (t, J = 5.2 Hz, 4H), 3.51 (t, J = 5.2 Hz, 4H), 2.42 (s, 3H). ^{13}C NMR (101 MHz, CDCl3) δ 154.2, 147.3, 139.8, 138.9, 134.7, 129.2, 127.8, 124.6, 119.7, 114.9, 66.2, 44.5, 21.4.

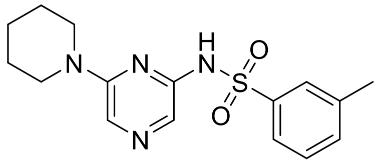

3-methyl-N-(6-(piperidin-1-yl)pyrazin-2-yl)benzenesulfonamide (8)

6-(piperidin-1-yl)pyrazin-2-amine (50.0 mg, 0.281 mmol), DMAP (6.85 mg, 0.056 mmol), and 3-methylbenzenesulfonyl chloride (0.061 ml, 0.421 mmol) were reacted according to **GP2** to obtain 3-methyl-N-(6-(piperidin-1-yl)pyrazin-2-yl)benzenesulfonamide (40 mg, 0.120 mmol, 43 % yield) as a grey solid. LC-MS (ESI) m/z calcd for $C_{16}H_{20}N_4O_2S$ $[M + H]^+$ 333.13, found 333.10. ^{1}H NMR (400 MHz, DMSO) δ 11.05 (s, 1H), 7.79 (s, 1H), 7.72 – 7.71 (m, 1H), 7.69 – 7.65 (m, 1H), 7.49 – 7.43 (m, 2H), 7.40 (s, 1H), 3.37 (t, J = 5.5 Hz, 4H), 2.37 (s, 3H), 1.60 – 1.49 (m, 2H), 1.47 – 1.34 (m, 4H). 13C NMR (101 MHz, DMSO) δ 152.4, 145.7, 140.6, 138.7, 133.4, 129.0, 127.1, 124.0, 123.9, 119.3, 44.7, 24.8, 24.0, 20.8.

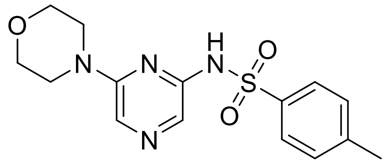

4-methyl-N-(6-morpholinopyrazin-2-yl)benzenesulfonamide (9)

A solution of 6-morpholinopyrazine-2-amine (0.1 mmol, 18,02 mg) (dissolved in 0.15 mL pyridine) was added dropwise to p-toluenesulfonyl chloride (0.2 mmol, 38.13 mg) dissolved in acetone (0.1 mL). The reaction mixture was stirred at rt. After 3h and 19h additional p-toluenesulfonyl chloride was added (0.02 mmol, 3.8 mg and 0.03 mmol, 5.7 mg, respectively). The completion of the reaction was confirmed by TLC (6:4 EtOAc/petroleum ether). Upon reaction completion, pyridine and acetone were evaporated under reduced pressure. EtOAc (25 mL) was added to the reaction mixture and was washed with 1M HCl (2x 25 mL). The aqueous layer was back extracted with EtOAc (3x50 mL). The combined organic layers were dried over $MgSO_4$, filtered and evaporated under reduced pressure. The crude mixture was purified by flash column chromatography using a gradient elution of 50-100% EtOAc/petroleum to obtain 4-methyl-N-(6-morpholinopyrazin-2-yl)benzenesulfonamide (0.0142 g, 0.041 mmol, 41 % yield) as a white powder. LC-MS (ESI) m/z calcd for $C_{15}H_{18}N_4O_3S$ $[M + H]^+$ 335.11, found 334.95. ^{1}H NMR (400 MHz, DMSO) δ 11.10 (s, 1H), 7.80 (s, 1H), 7.78 (d, J = 8.1 Hz, 2H), 7.50 (s, 1H), 7.38 (d, J = 8.2 Hz, 2H), 3.59 (t, J = 5.2, 4.5 Hz, 4H), 3.38 – 3.31 (m, 4H), 2.36 (s, 3H). ^{13}C NMR (101 MHz, DMSO) δ 152.5, 145.7, 143.4,137.6, 129.5, 127.1, 123.9, 120.5, 65.6, 44.2, 21.0.

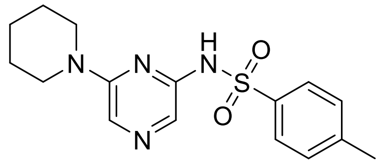

4-methyl-N-(6-(piperidin-1-yl)pyrazin-2-yl)benzenesulfonamide (10)

6-(piperidin-1-yl)pyrazin-2-amine (0.053 g, 0.3 mmol), pyridine (0.048 ml, 0.600 mmol), and 4-methylbenzenesulfonyl chloride (0.114 g, 0.600 mmol) were reacted according to **GP3** to obtain 4-methyl-N-(6-(piperidin-1-yl)pyrazin-2-yl) benzenesulfonamide (0.024 g, 0.070 mmol, 23% yield) as a yellow solid. LC-MS (ESI) m/z calcd for $C_{16}H_{20}N_4O_2S$ $[M + H]^+$ 333.13, found 333.15. ^1H NMR (400 MHz, DMSO) δ 11.00 (s, 1H), 7.77 (d, J = 8.5 Hz, 3H), 7.40 (s, 1H), 7.37 (d, J = 8.0 Hz, 2H), 3.38 (t, J = 5.2 Hz, 4H), 2.35 (s, 3H), 1.55 (p, J = 5.7 Hz, 2H), 1.39 (p, J = 3.7 Hz, 4H). ^{13}C NMR (101 MHz, DMSO) δ 152.4, 145.8, 143.7, 137.8, 130.0, 127.0, 123.9, 119.2, 44.8, 24.8, 24.1, 21.0.

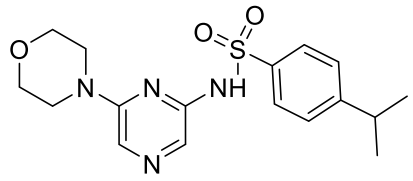

4-isopropyl-N-(6-morpholinopyrazin-2-yl)benzenesulfonamide (11)

6-morpholinopyrazin-2-amine (50.0 mg, 0.277 mmol), DMAP (6.78 mg, 0.055 mmol), and 4-isopropylbenzenesulfonyl chloride (0.099 ml, 0.555 mmol) were reacted according to **GP2** to obtain 4-isopropyl-N-(6-morpholinopyrazin-2-yl)benzenesulfonamide (60 mg, 0.166 mmol, 60 % yield) as an off-white solid. LC-MS (ESI) m/z calcd for $C_{17}H_{22}N_4O_3S$ $[M + H]^+$ 363.14, found 363.10. ^1H NMR (400 MHz, DMSO) δ 11.13 (br s, 1H), 7.83 - 7.79 (m, 3H), 7.52 (s, 1H), 7.49 – 7.45 (m, 2H), 3.58 (dd, J = 5.1, 4.6 Hz, 4H), 3.34 – 3.30 (m, 4H), 2.97 (sep, J = 6.9 Hz, 1H), 1.20 (d, J = 6.9 Hz, 6H). ^{13}C NMR (101 MHz, DMSO) δ 153.8, 152.5, 145.7, 138.1, 127.1, 127.0, 124.0, 120.5, 65.6, 44.1, 33.3, 23.4.

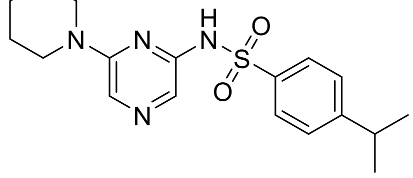

4-isopropyl-N-(6-(piperidin-1-yl)pyrazin-2-yl) benzenesulfonamide (12)

6-(piperidin-1-yl)pyrazin-2-amine (53.5 mg, 0.3 mmol), pyridine (0.048 ml, 0.600 mmol), 4-isopropylbenzenesulfonyl chloride (131 mg, 0.600 mmol) were reacted according to **GP3** 4-isopropyl-N-(6-(piperidin-1-yl)pyrazin-2-yl) benzenesulfonamide (19.6 mg, 0.052 mmol, 17% yield) as a yellow powder. LC-MS (ESI) m/z calcd for $C_{18}H_{24}N_4O_2S$ $[M + H]^+$ 361.16, found 361.15. ^1H NMR (400 MHz, DMSO) δ 11.03 (s, 1H), 7.81 (s, 1H), 7.79 (s, 2H), 7.45 (d, J = 8.4 Hz, 2H), 7.42 (s, 1H), 3.36 (t, J = 4.5 Hz,

4H), 2.95 (hept, J = 6.9 Hz, 1H), 1.54 (p, J = 5.7 Hz, 2H), 1.36 (p, J=5.7 Hz, 4H), 1.19 (d, J = 6.9 Hz, 6H). ^{13}C NMR (101 MHz, DMSO) δ 153.7, 152.4, 145.8, 138.3, 127.1, 127.0, 123.97, 119.3, 44.8, 33.4, 24.8, 24.1, 23.5.

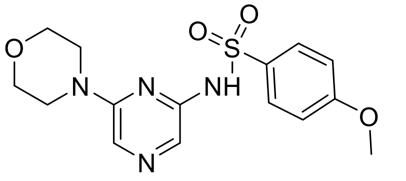

4-methoxy-N-(6-morpholinopyrazin-2-yl)benzenesulfonamide (13)

6-morpholinopyrazin-2-amine (50.0 mg, 0.277 mmol), DMAP (6.78 mg, 0.055 mmol), and 4-methoxybenzenesulfonyl chloride (115 mg, 0.555 mmol) were reacted according to **GP2** to obtain 4-methoxy-N-(6-morpholinopyrazin-2-yl)benzenesulfonamide (22 mg, 0.063 mmol, 22% yield) as a white solid. LC-MS (ESI) m/z calcd for $C_{15}H_{18}N_4O_4S$ [M + H]+ 351.39, found 351.10. ^{1}H NMR (400 MHz, DMSO) δ 11.03 (s, 1H), 7.83 (d, J = 8.8 Hz, 2H), 7.81 (s, 1H), 7.50 (s, 1H), 7.14 – 7.06 (m, 2H), 3.81 (s, 3H), 3.61 (t, J = 4.9 Hz, 4H), 3.36 (t, J = 4.9 Hz, 4H). ^{13}C NMR (101 MHz, DMSO) δ 162.6, 152.6, 145.9, 132.1, 129.3, 123.7, 120.6, 114.3, 66.1, 65.6, 55,7, 44.1.

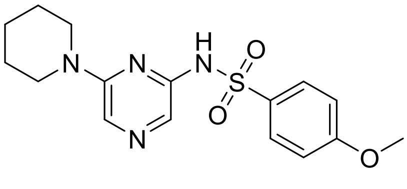

4-methoxy-N-(6-(piperidin-1-yl)pyrazin-2-yl)benzenesulfonamide (14)

6-(piperidin-1-yl)pyrazin-2-amine (53.5 mg, 0.3 mmol), DMAP (3.67 mg, 0.0300 mmol), and 4-methoxybenzenesulfonyl chloride (124 mg, 0.600 mmol) were reacted according to **GP2** 4-methoxy-N-(6-(piperidin-1-yl)pyrazin-2-yl)benzenesulfonamide (6.1 mg, 0.017 mmol, 6% yield) as a yellow solid. LC-MS (ESI) m/z calcd for $C_{16}H_{20}N_4O_3S$ $[M + H]^+$ 349.12, found 349.10. ^{1}H NMR (400 MHz, DMSO) δ 10.94 (s, 1H), 7.83 (d, J = 9.1 Hz, 2H), 7.80 (s, 1H), 7.39 (s, 1H), 7.09 (d, J = 8.9 Hz, 1H), 3.81 (s, 4H), 3.41 (t, J = 5.5 Hz, 3H), 1.57 (p, J = 5.7 Hz, 2H), 1.41 (p, J = 5.6 Hz, 4H). ^{13}C NMR (101 MHz, DMSO) δ 162.5, 152.5, 145.9, 132.2, 129.2, 123.6, 118.9, 114.3, 55.7, 44.8, 24.8, 24.1.

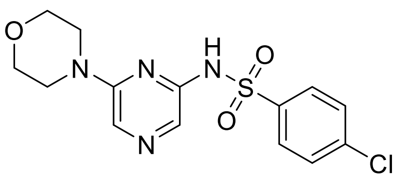

4-chloro-N-(6-morpholinopyrazin-2-yl)benzenesulfonamide (15)

6-morpholinopyrazin-2-amine (0.054 g, 0.3 mmol) and 4-chlorobenzenesulfonyl chloride (0.127 g, 0.600 mmol) were reacted according **GP3** to obtain 4-chloro-N-(6-morpholinopyrazin-2-

yl)benzenesulfonamide (0.0083 g, 0.023 mmol, 8% yield) as a yellow powder. LC-MS (ESI) m/z calcd for $C_{14}H_{15}ClN_4O_3S$ [M + H]+ 355.05, found 355,05. ^{1}H NMR (400 MHz, DMSO) δ 11.30 (s, 1H), 7.90 (d, J = 8.7 Hz, 2H), 7.84 (s, 1H), 7.68 (d, J = 9.2 Hz, 2H), 7.50 (s, 1H), 3.59 (t, J = 4.9 Hz, 4H), 3.33 (t, J = 4.9 Hz, 4H). 13C NMR (101 MHz, DMSO) δ 152.6, 145.5, 139.5, 137.9, 129.4, 129.0, 124.4, 120.6, 65.6, 44.2.

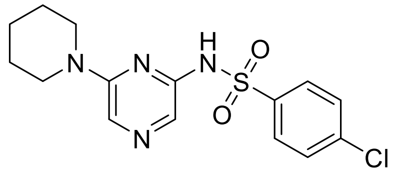

4-chloro-N-(6-(piperidin-1-yl)pyrazin-2-yl)benzenesulfonamide (16)

6-(piperidin-1-yl)pyrazin-2-amine (53.5 mg, 0.3 mmol), pyridine (0.048 ml, 0.600 mmol), and 4-chlorobenzenesulfonyl chloride (127 mg, 0,600 mmol) were reacted according to **GP3** to obtain 4-chloro-N-(6-(piperidin-1-yl)pyrazin-2-yl)benzenesulfonamide (23.2 mg, 0.065 mmol, 22% yield) as a yellow solid. LC-MS (ESI) m/z calcd for $C_{15}H_{17}ClN_4O_2S$ $[M + H]^+$ 353.08, found 353.05. ^{1}H NMR (400 MHz, DMSO) δ 11.21 (s, 1H), 7.89 (d, J = 8.6 Hz, 2H), 7.82 (s, 1H), 7.67 (d, J = 8.6 Hz, 2H), 7.41 (s, 1H), 3.36 (t, J = 5.4 Hz, 4H), 1.54 (p, J = 6.1 Hz, 2H), 1.38 (p, J = 8.0 Hz, 4H). ^{13}C NMR (101 MHz, DMSO) δ 152.3, 145.5, 139.7, 137.7, 129.3, 128.8, 124.3, 119.3, 44.8, 24.8, 24.1.

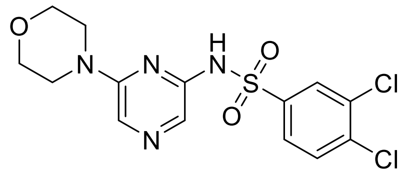

3,4-dichloro-N-(6-morpholinopyrazin-2-yl)benzenesulfonamide (17)

6-morpholinopyrazin-2-amine (0.054 g, 0.3 mmol) and 3,4-dichlorobenzenesulfonyl chloride (0.147 g, 0.600 mmol) reacted according **GP3** to obtain 3,4-dichloro-N-(6-morpholinopyrazin-2-yl)benzenesulfonamide (0.0032 g, 8.21 µmol, 3 % yield) as a light yellow powder. LC-MS (ESI) m/z calcd for $C_{14}H_{14}Cl_2N_4O_3S$ $[M + H]^+$ 389.01, found 389.00. ^{1}H NMR (400 MHz, DMSO) δ 11.44 (s, 1H), 8.12 (d, J = 2.1 Hz, 1H), 7.90 (d, J = 8.4 Hz, 1H), 7.88 (s, 1H), 7.83 (dd, J = 8.5, 2.1 Hz, 1H), 7.51 (s, 1H), 3.62 (t, J = 4.85 Hz, 4H), 3.33 (t, J = 4.9 Hz, 4H). ^{13}C NMR (101 MHz, DMSO) δ 152.7,145.8, 141.3, 135.9, 132.2, 131.8,129.1, 127.0, 124.3, 121.3, 120.9, 65.6, 44.3.

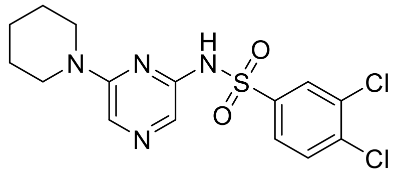

3,4-dichloro-N-(6-(piperidin-1-yl)pyrazin-2-yl) benzenesulfonamide (18)

6-(piperidin-1-yl)pyrazin-2-amine (53.5 mg, 0.3 mmol), pyridine (0.048 ml, 0.600 mmol), and 3,4-dichlorobenzenesulfonyl chloride (147 mg, 0.600 mmol) were reacted according to **GP3** to obtain 3,4-dichloro-N-(6-(piperidin-1-yl)pyrazin-2-yl) benzenesulfonamide (37.5 mg, 0.096 mmol, 32% yield) as a yellow solid. LC-MS (ESI) m/z calcd for $C_{15}H_{16}Cl_2N_4O_2S$ $[M + H]^+$ 387.03, found 387.00. ^1H NMR (400 MHz, DMSO) δ 11.35 (s, 1H), 8.09 (d, J = 2.1 Hz, 1H), 7.89 (d, J = 8.4 Hz, 1H), 7.86 (s, 1H), 7.83 (dd, J = 8.5, 2.1 Hz, 1H), 7.42 (s, 1H), 3.36 (t, J = 5.2 Hz, 4H), 1.56 (p, J = 5.7 Hz, 2H), 1.39 (p, J = 5.7 Hz, 4H). ^{13}C NMR (101 MHz, DMSO) δ 153.1, 145.3, 141.5, 136.3, 131.9, 131.8, 128.9, 126.9, 124.8, 119.67, 44.8, 24.7, 24.0.

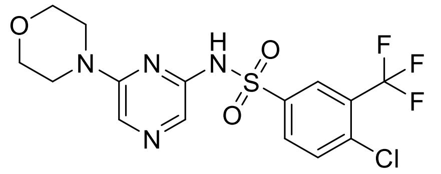

4-chloro-N-(6-morpholinopyrazin-2-yl)-3-(trifluoromethyl)benzenesulfonamide (19)

6-morpholinopyrazin-2-amine (0.054 g, 0.3 mmol), DMAP (3.67 mg, 0.030 mmol), and 4-chloro-3-(trifluoromethyl)benzenesulfonyl chloride (0.184 g, 0.660 mmol) were reacted according to **GP2** to obtain 4-chloro-N-(6-morpholinopyrazin-2-yl)-3-(trifluoromethyl)benzenesulfonamide (0.0297 g, 0.069 mmol, 23% yield) as a white powder. LC-MS (ESI) m/z calcd for $C_{15}H_{14}ClF_3N_4O_3S$ $[M + H]^+$ 423,04, found 423,00. ^1H NMR (400 MHz, DMSO) δ 11.53 (s, 1H), 8.25 (d, J = 2.1 Hz, 1H), 8.17 (dd, J = 8.5, 2.2Hz, 1H), 8.00 (d, J = 8.4 Hz, 1H), 7.89 (s, 1H), 7.52 (s, 1H), 3.60 (t, J = 4.9 Hz, 4H), 3.29 (t, J = 4.6 Hz,4H). ^{13}C NMR (101 MHz, DMSO) δ 153.0, 145.7, 140.9, 135.7, 133.3, 132.6, 127.4, 127.1, 126.5,126.4, 126.3, 126.3, 126.2, 125.0, 123.5, 121.2, 120.7, 65.5, 44.1.

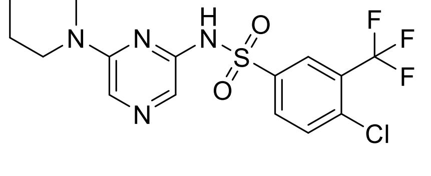

4-chloro-N-(6-(piperidin-1-yl)pyrazin-2-yl)-3-(trifluoromethyl)benzenesulfonamide (20)

6-(piperidin-1-yl)pyrazin-2-amine (53.5 mg, 0.3 mmol), pyridine (0.048 ml, 0.600 mmol), and 4-chloro-3-(trifluoromethyl)benzenesulfonyl chloride (167 mg, 0,600 mmol) were reacted according to **GP3** to obtain 4-chloro-N-(6-(piperidin-1-yl)pyrazin-2-yl)-3-(trifluoromethyl)benzenesulfonamide (29 mg, 0.068 mmol, 23% yield) as a yellow solid. LC-MS (ESI) m/z calcd for $C_{16}H_{16}ClF_3N_4O_2S$ $[M + H]^+$ 421.06, found 421.05. ^1H NMR (400 MHz, DMSO) δ 11.44 (s, 1H), 8.23 (d, J = 2.2 Hz, 1H), 8.15 (dd, J = 8.4, 2.3 Hz, 1H), 7.99 (d, J = 8.5 Hz, 1H), 7.87 (s, 1H), 7.43 (s, 1H), 3.31 (t, J = 5.5 Hz, 4H), 1.53 (p, J = 5.7 Hz, 2H),

1.37 (p, J = 4.3 Hz, 4H). ^{13}C NMR (101 MHz, DMSO) δ 152.4, 145.2, 140.6, 135.5, 133.3, 132.4, 127.7, 127.4, 127.1, 126.8, 126.2, 126.2, 126.1, 126.1, 125.0, 123.5, 120.7, 119.9, 44.8, 24.6, 23.9.
