# Supplementary material for: Combining AlphaFold with Focused Virtual Library Design in the Development of Novel CCR2 and CCR5 Antagonists
Source: J Chem Inf Model. 2025 Nov 12;65(22):12398–409. doi: 10.1021/acs.jcim.5c01596 (PMC12648652; doi:10.1021/acs.jcim.5c01596)
Supplement: Supplementary file 3 [file ci5c01596_si_003.pdf]

Combining AlphaFold with Focused Virtual Library Design in the Development of Novel CCR2 and CCR5 Antagonists: Supplementary Information

Khaled Essa[1,2], Kian Noorman van der Dussen [1], Yao Yao [1], Bente Bleijs[1], Natalia Ortiz Zacarias [1,2], Laura H. Heitman [1,2], Gerard van Westen [1], Willem Jespers[3], Daan van der Es [1], Martin Šícho[1,4]*.*

[1]Division of Medicinal Chemistry, Leiden Academic Centre for Drug Research (LACDR), Leiden University, 2333 CC Leiden, The Netherlands.

[2]Oncode Institute, 2333 CC Leiden, The Netherlands

[3]Department of Medicinal Chemistry, Photopharmacology and Imaging, Groningen, Research Institute of Pharmacy (GRIP), Faculty of Science and Engineering, Antonius Deusinglaan 1, 9713 AV Groningen, The Netherlands

[4]CZ-OPENSCREEN: National Infrastructure for Chemical Biology, Department of Informatics and Chemistry, Faculty of Chemical Technology, University of Chemistry and Technology Prague, Technická 5, 166 28, Prague, Czech Republic

Corresponding Authors

*Martin Šícho (martin.sicho@vscht.cz), *Daan van der Es (d.van.der.es@lacdr.leidenuniv.nl)

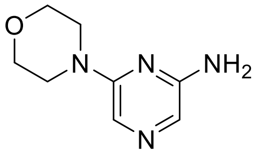

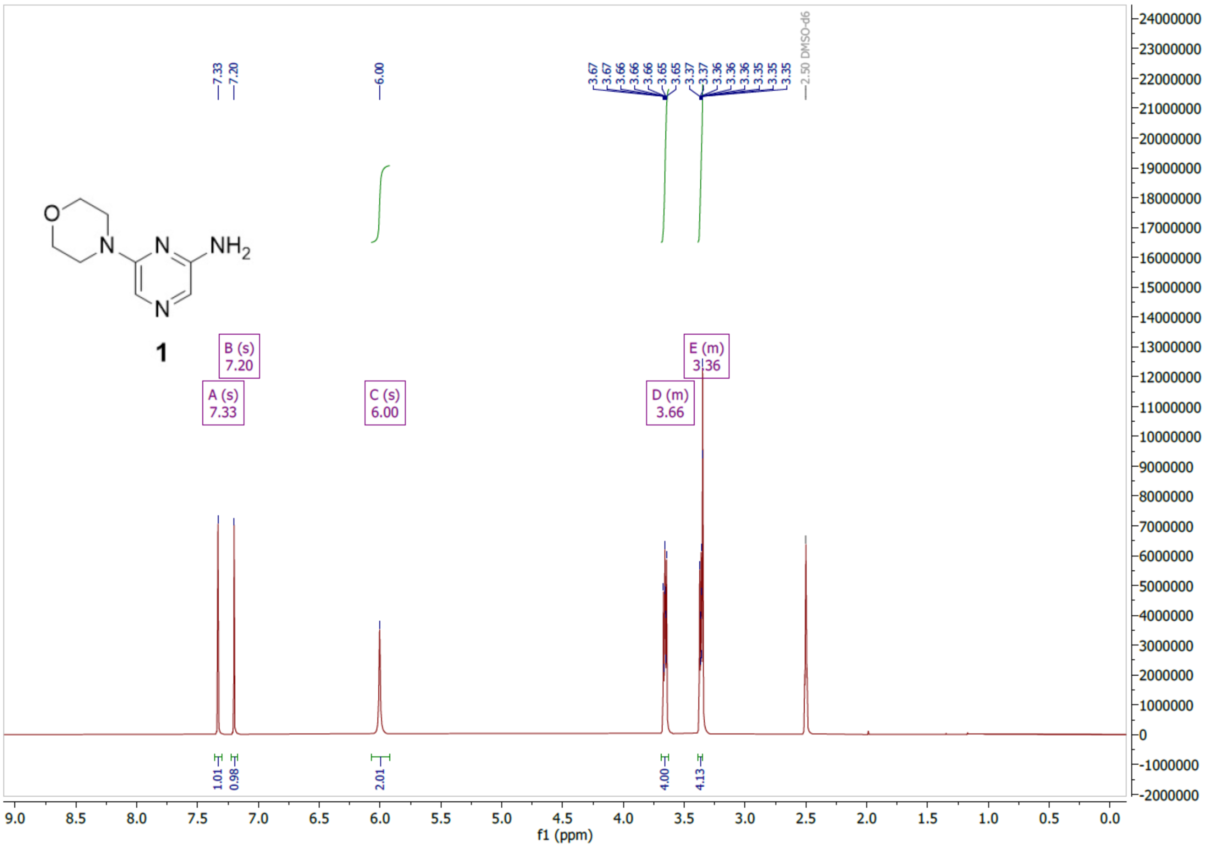

7.33
7.20
6.00
3.67
3.67
3.66
3.66
3.66
3.65
3.65
3.37
3.37
3.36
3.36
3.36
3.35
3.35
3.35
2.50 DMSO-d6
1
A (s)
7.33
B (s)
7.20
C (s)
6.00
D (m)
3.66
E (m)
3.36
1.01
0.98
2.01
4.00
4.13
f1 (ppm)

==== Shimadzu LabSolutions Browser Report ====

Chromatogram(KE104-pure.lcd)

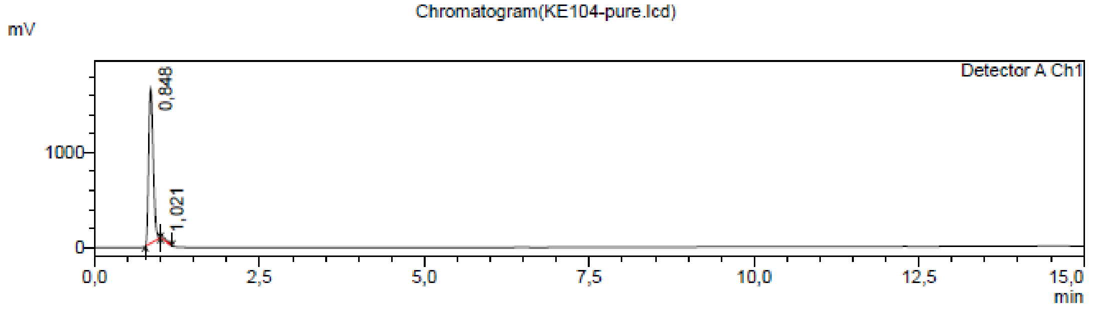

MS Chromatogram(KE104-pure.lcd)

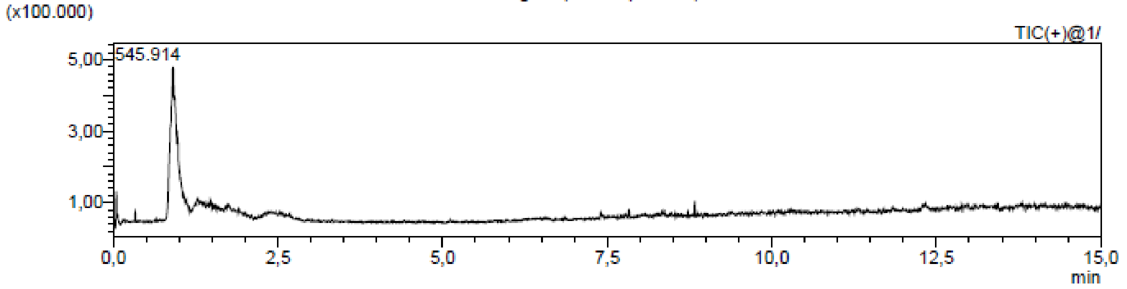

MS Spectrum(KE104-pure.lcd)

Ret. Time: 1-1(E+) [0,887]

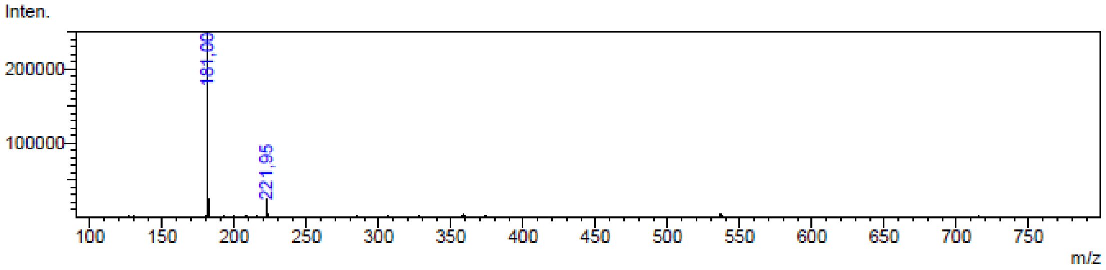

Peak Table(KE104-pure.lcd)

Detector A 254nm

Peak#	Ret. Time	Area	Height	Mark	Conc.	Area%
1	0,848	7751180	1621661	M	98,570	98,570
2	1,021	112440	21091	M	1,430	1,430
Total		7863620	1642751		100,000	100,000

Sample Information(KE104-pure.lcd)

**Data File Name:**	KE104-pure.lcd
**Method File Name:**	MSgeneral1090FA_LowMW.lcm
**Acquired by:**	System Administrator
**Date Acquired:**	26-1-2023 13:48:45
**Sample Name:**	KE104-pure
**Sample ID:**	KE104-pure
**Sample Type:**	Unknown
**Level#:**	0
**Detector:**	Detector A, MS
**Comment:**	

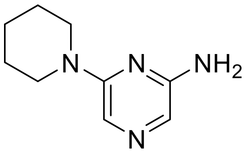
N
N
NH2
N

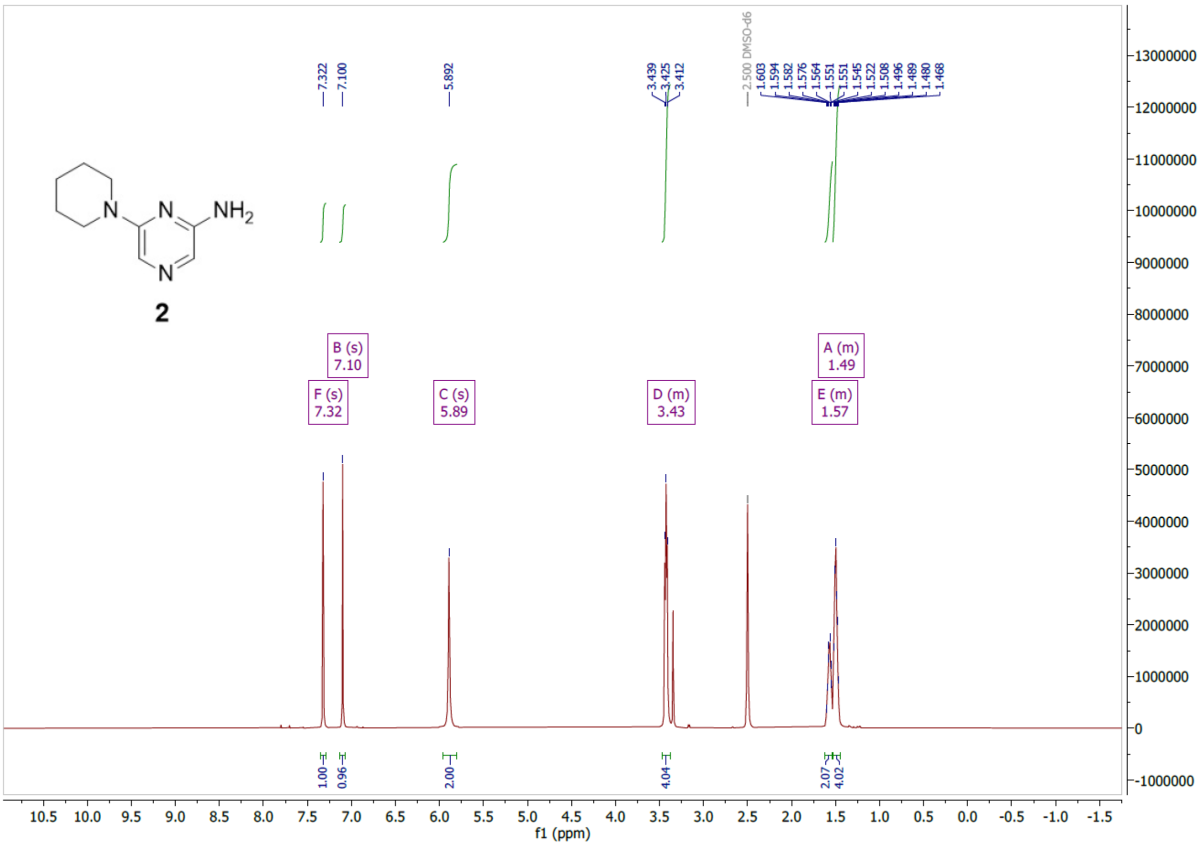
7.322
7.100
5.892
3.439
3.425
3.412
2.500 DMSO-d6
1.603
1.594
1.582
1.576
1.564
1.551
1.551
1.545
1.522
1.508
1.496
1.489
1.480
1.468
2
B (s) 7.10
F (s) 7.32
C (s) 5.89
D (m) 3.43
A (m) 1.49
E (m) 1.57
1.00
0.96
2.00
4.04
2.07
4.02
f1 (ppm)

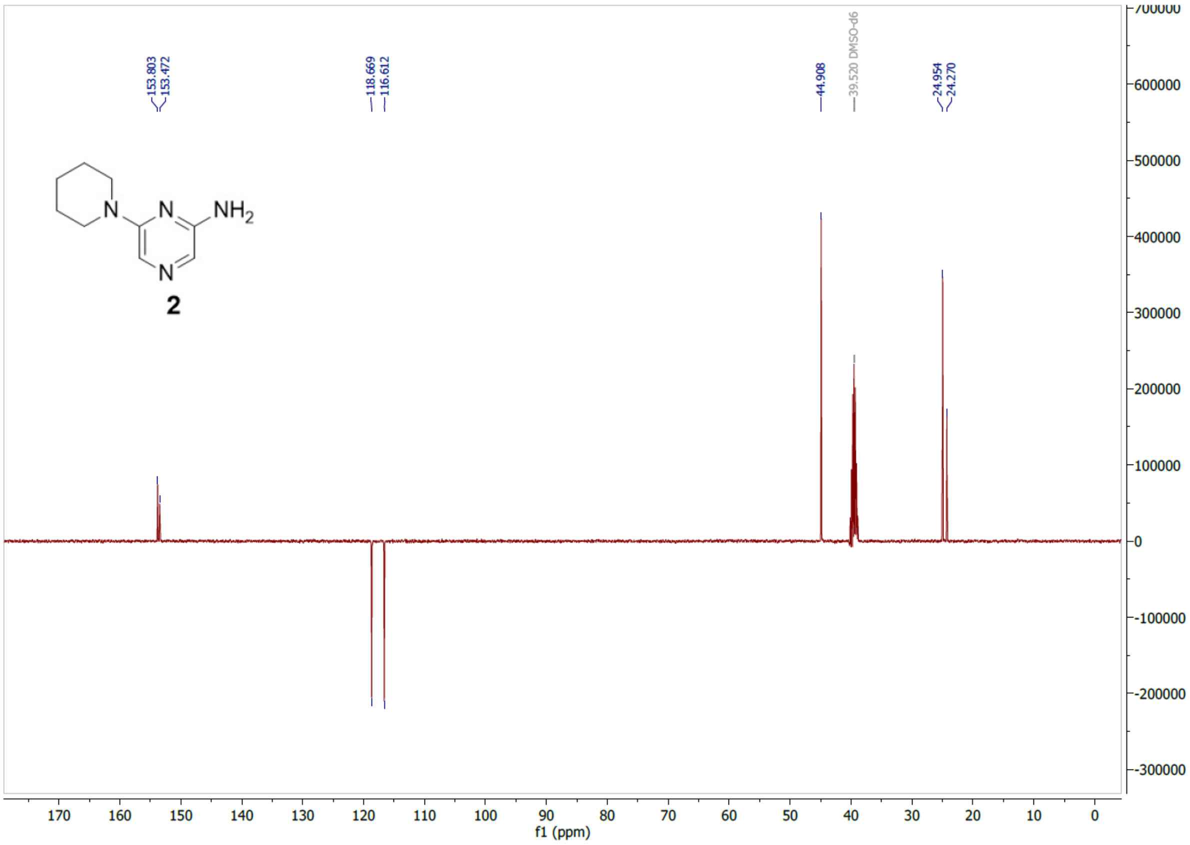
153.803
153.472
118.669
116.612
44.908
39.520 DMSO-d6
24.954
24.270
2
f1 (ppm)

==== Shimadzu LabSolutions Browser Report ====

Chromatogram(KE107-pure.lcd)

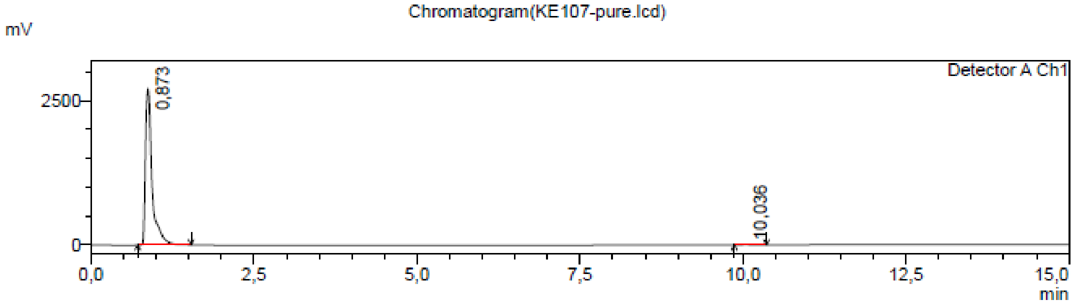

MS Chromatogram(KE107-pure.lcd)

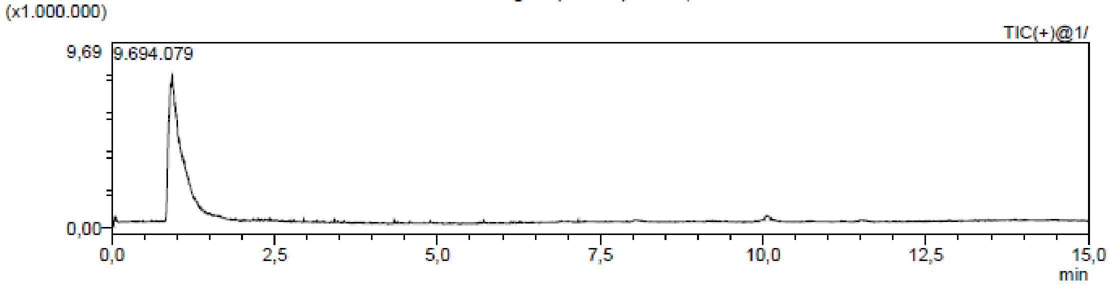

MS Spectrum(KE107-pure.lcd)

Ret. Time: 1-1(E+) [0,887]

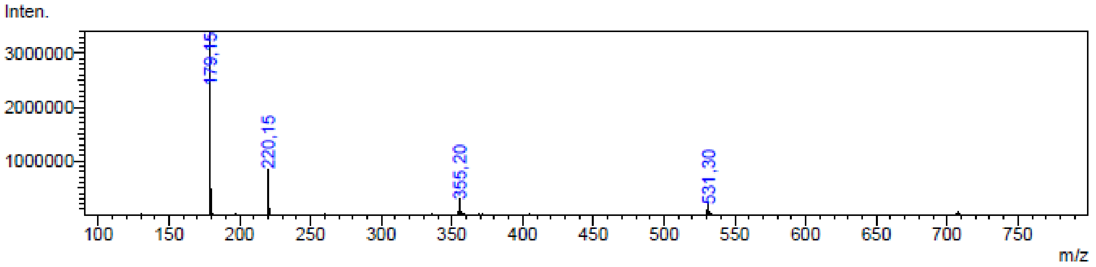

Peak Table(KE107-pure.lcd)

Detector A 254nm

Peak#	Ret. Time	Area	Height	Mark	Conc.	Area%
1	0,873	19020937	2697211	M	99,905	99,905
2	10,036	18092	2450	M	0,095	0,095
Total		19039029	2699661		100,000	100,000

Sample Information(KE107-pure.lcd)

**Data File Name:**	KE107-pure.lcd
**Method File Name:**	MSgeneral1090FA_LowMW.lcm
**Acquired by:**	System Administrator
**Date Acquired:**	3-2-2023 12:43:50
**Sample Name:**	KE107-pure
**Sample ID:**	KE107-pure
**Sample Type:**	Unknown
**Level#:**	0
**Detector:**	Detector A, MS
**Comment:**	

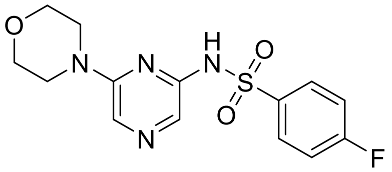

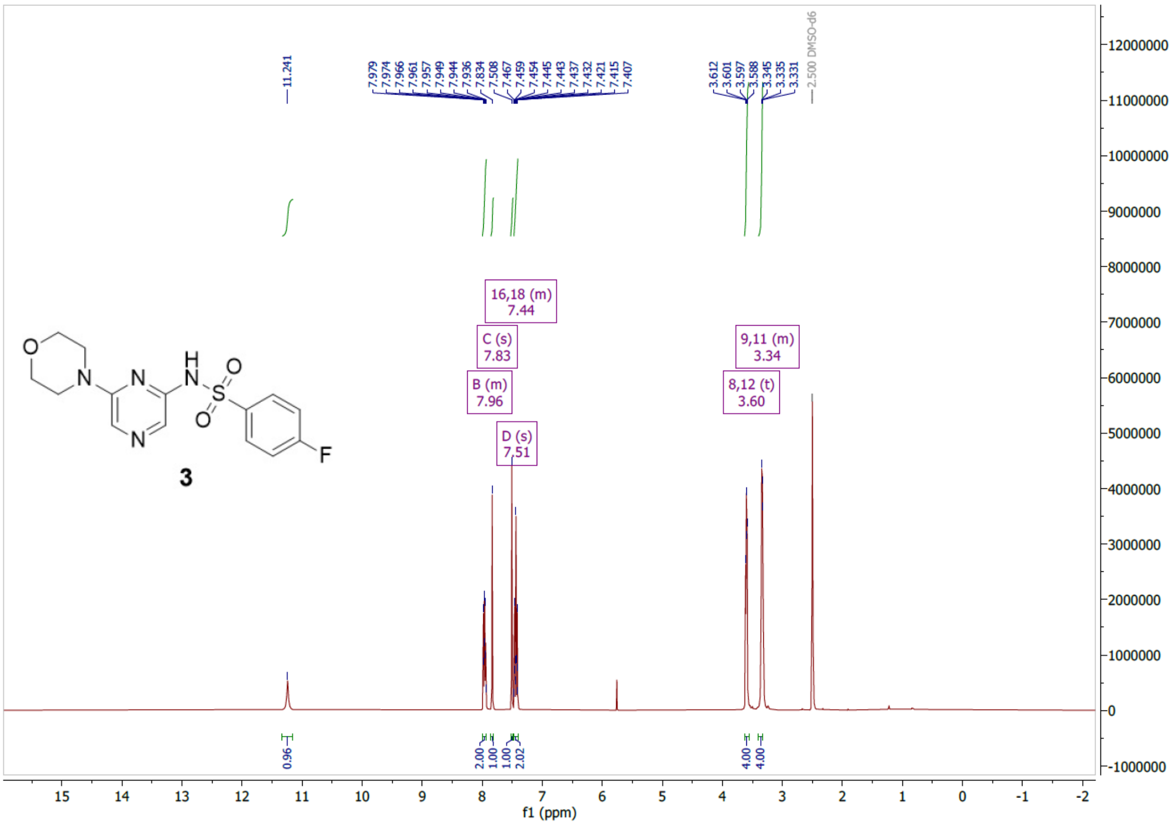

11.241
7.979
7.974
7.966
7.961
7.957
7.949
7.944
7.936
7.834
7.508
7.467
7.459
7.454
7.445
7.443
7.437
7.432
7.421
7.415
7.407
3.612
3.601
3.597
3.588
3.345
3.335
3.331
2.500 DMSO-d6
16,18 (m)
7.44
C (s)
7.83
B (m)
7.96
D (s)
7,51
9,11 (m)
3.34
8,12 (t)
3.60
3
0.96
2.00
1.00
1.00
2.02
4.00
4.00
f1 (ppm)

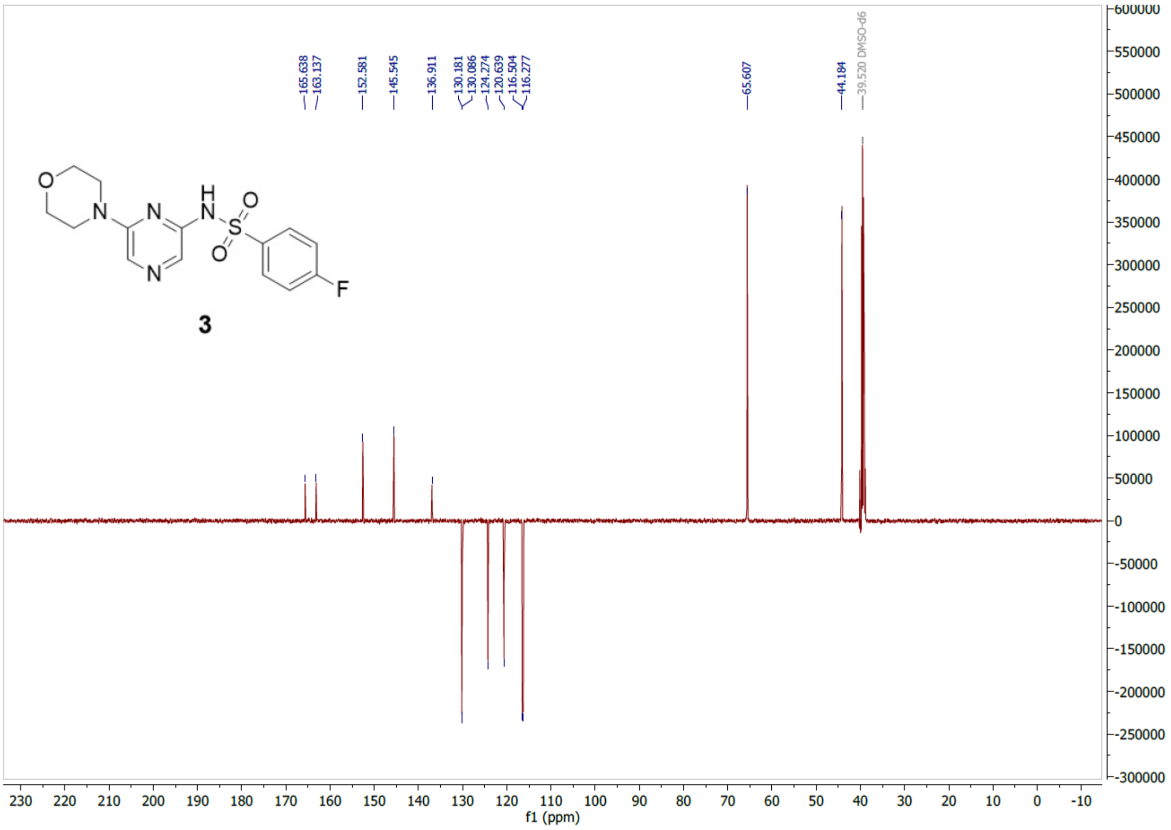

165.638
163.137
152.581
145.545
136.911
130.181
130.086
124.274
120.639
116.504
116.277
65.607
44.184
39.520 DMSO-d6
3
f1 (ppm)

==== Shimadzu LabSolutions Browser Report ====

Chromatogram(KE110-col1-pure.lcd)

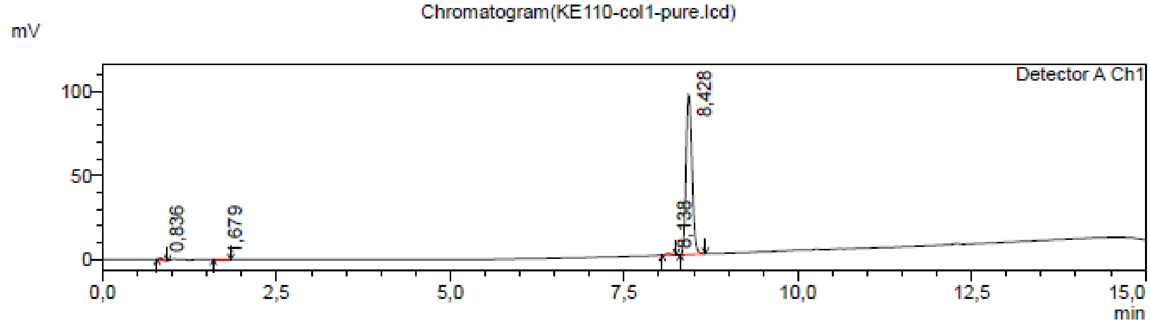

MS Chromatogram(KE110-col1-pure.lcd)

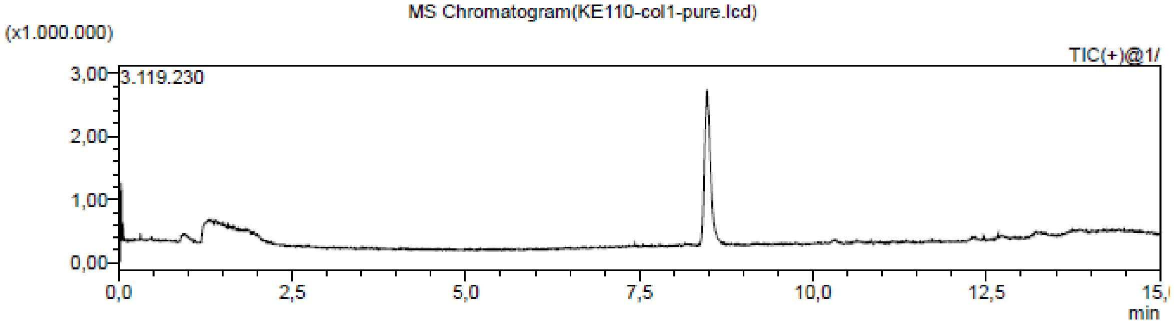

MS Spectrum(KE110-col1-pure.lcd)

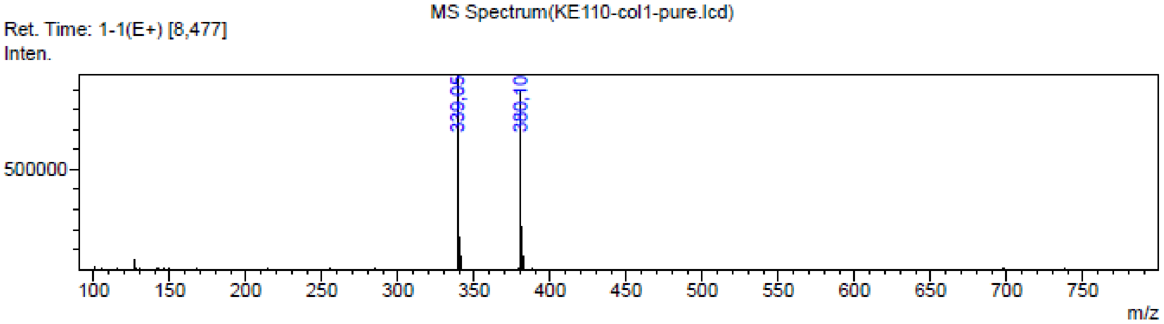

Peak Table(KE110-col1-pure.lcd)

Detector A 254nm

Peak#	Ret. Time	Area	Height	Mark	Conc.	Area%
1	0,836	2257	975	M	0,407	0,407
2	1,679	237	26	M	0,043	0,043
3	8,138	5220	904	M	0,940	0,940
4	8,428	547334	95290	M	98,610	98,610
Total		555049	97195		100,000	100,000

Sample Information(KE110-col1-pure.lcd)

**Data File Name:**	KE110-col1-pure.lcd
**Method File Name:**	MSgeneral1090FA_LowMW.lcm
**Acquired by:**	System Administrator
**Date Acquired:**	13-2-2023 12:34:18
**Sample Name:**	KE110-col1-pure
**Sample ID:**	KE110-col1-pure
**Sample Type:**	Unknown
**Level#:**	0
**Detector:**	Detector A, MS
**Comment:**	

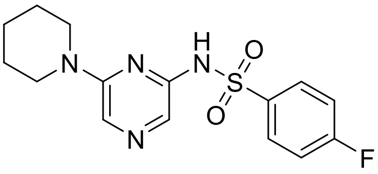

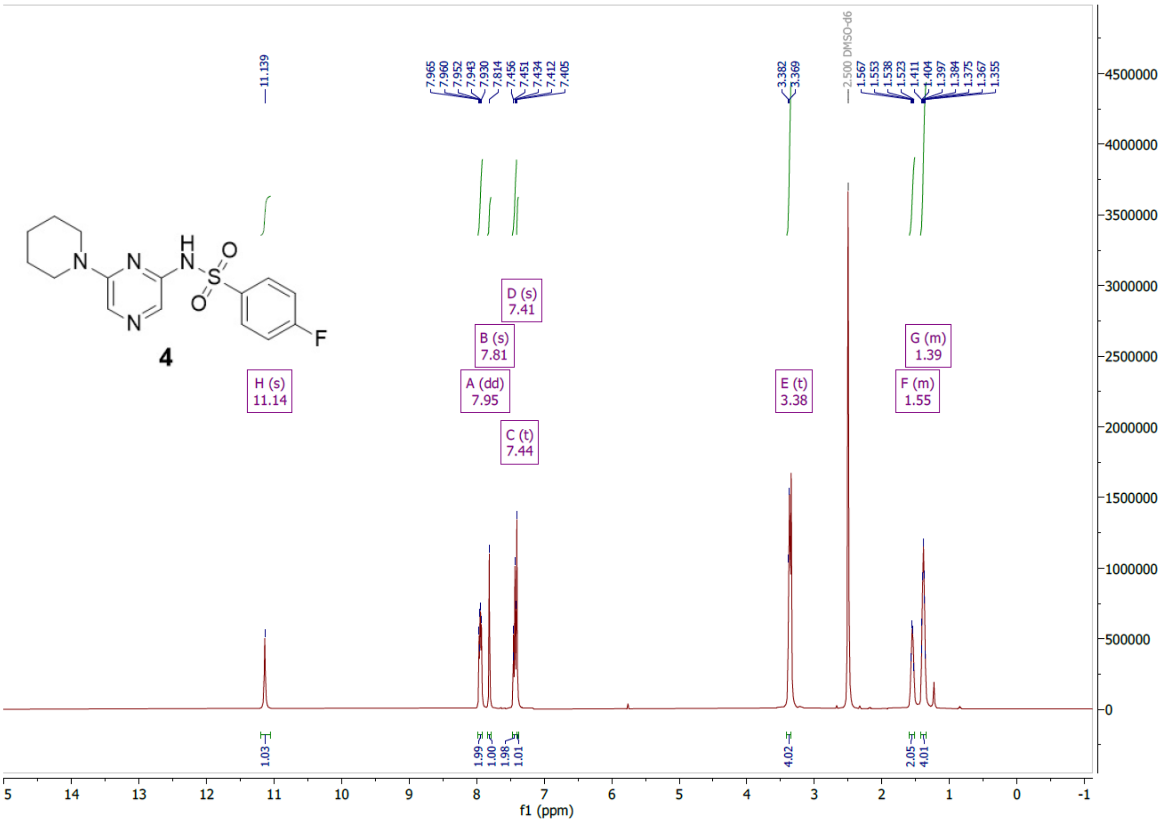

11.139
7.965
7.960
7.952
7.943
7.930
7.814
7.456
7.451
7.434
7.412
7.405
3.382
3.369
2.500 DMSO-d6
1.567
1.553
1.538
1.523
1.411
1.404
1.397
1.384
1.375
1.367
1.355
4
D (s)
7.41
B (s)
7.81
A (dd)
7.95
C (t)
7.44
H (s)
11.14
E (t)
3.38
G (m)
1.39
F (m)
1.55
1.03
1.99
1.00
1.98
1.01
4.02
2.05
4.01
f1 (ppm)

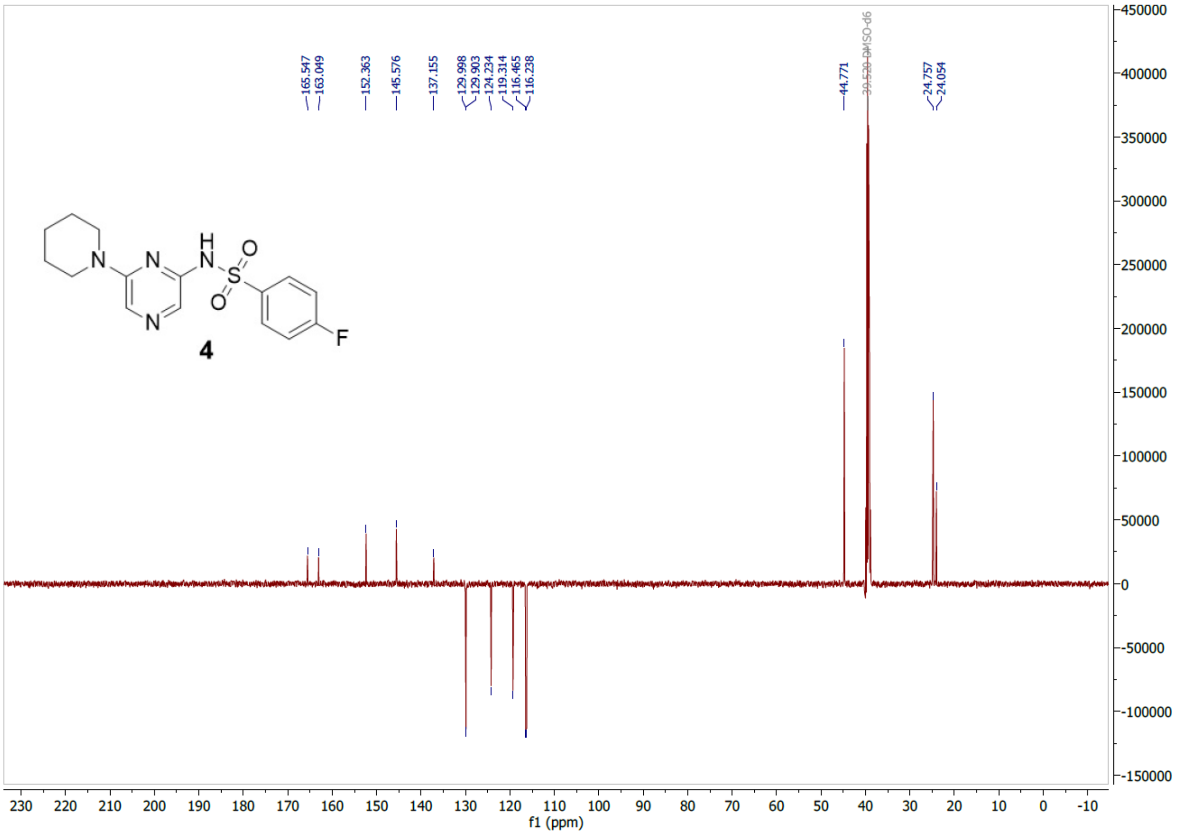

165.547
163.049
152.363
145.576
137.155
129.998
129.903
124.234
119.314
116.465
116.238
44.771
39.520 DMSO-d6
24.757
24.054
4
f1 (ppm)

==== Shimadzu LabSolutions Browser Report ====

Chromatogram(KE115-pure.lcd)

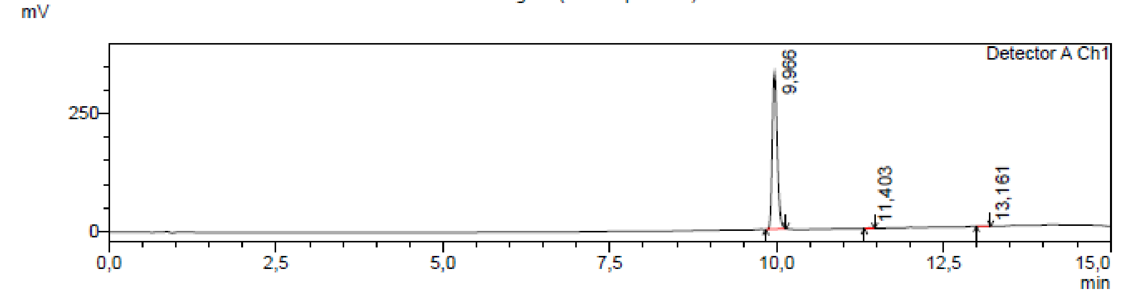

MS Chromatogram(KE115-pure.lcd)

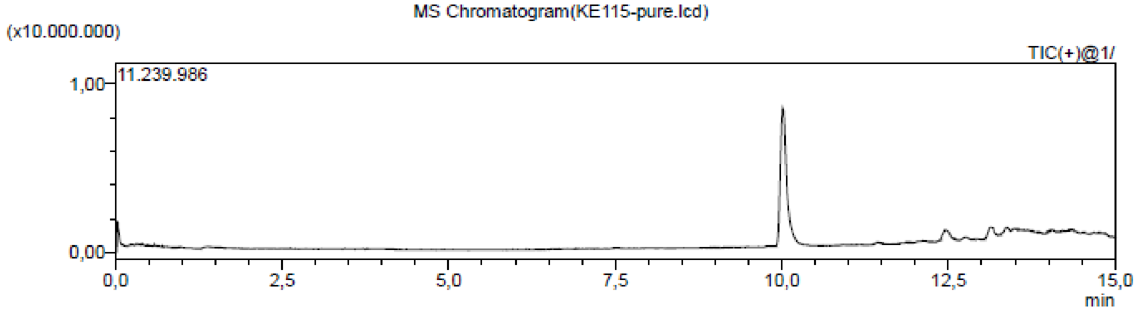

MS Spectrum(KE115-pure.lcd)

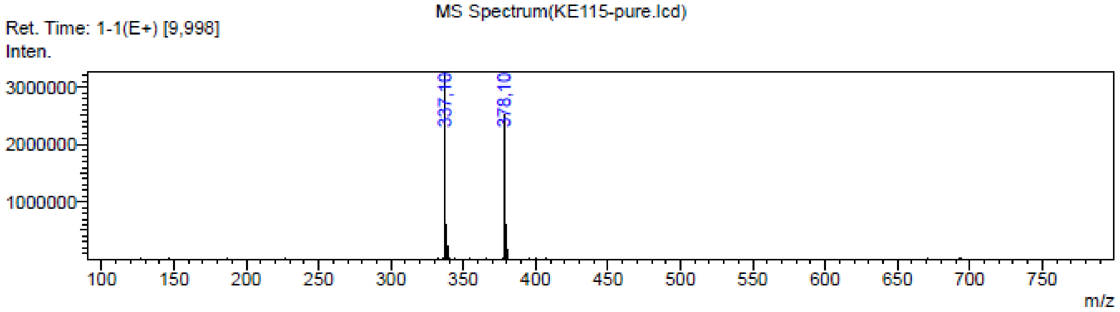

Peak Table(KE115-pure.lcd)

Detector A 254nm

Peak#	Ret. Time	Area	Height	Mark	Conc.	Area%
1	9,966	1724770	330134	M	99,597	99,597
2	11,403	6654	1505	M	0,384	0,384
3	13,161	333	26	M	0,019	0,019
Total		1731757	331665		100,000	100,000

Sample Information(KE115-pure.lcd)

**Data File Name:**	KE115-pure.lcd
**Method File Name:**	MSgeneral1090FA_LowMW.lcm
**Acquired by:**	System Administrator
**Date Acquired:**	23-2-2023 16:32:28
**Sample Name:**	KE115-pure
**Sample ID:**	KE115-pure
**Sample Type:**	Unknown
**Level#:**	0
**Detector:**	Detector A, MS
**Comment:**	

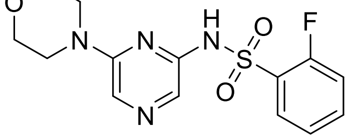
O
N
N
N
H
N
S
O
O
F

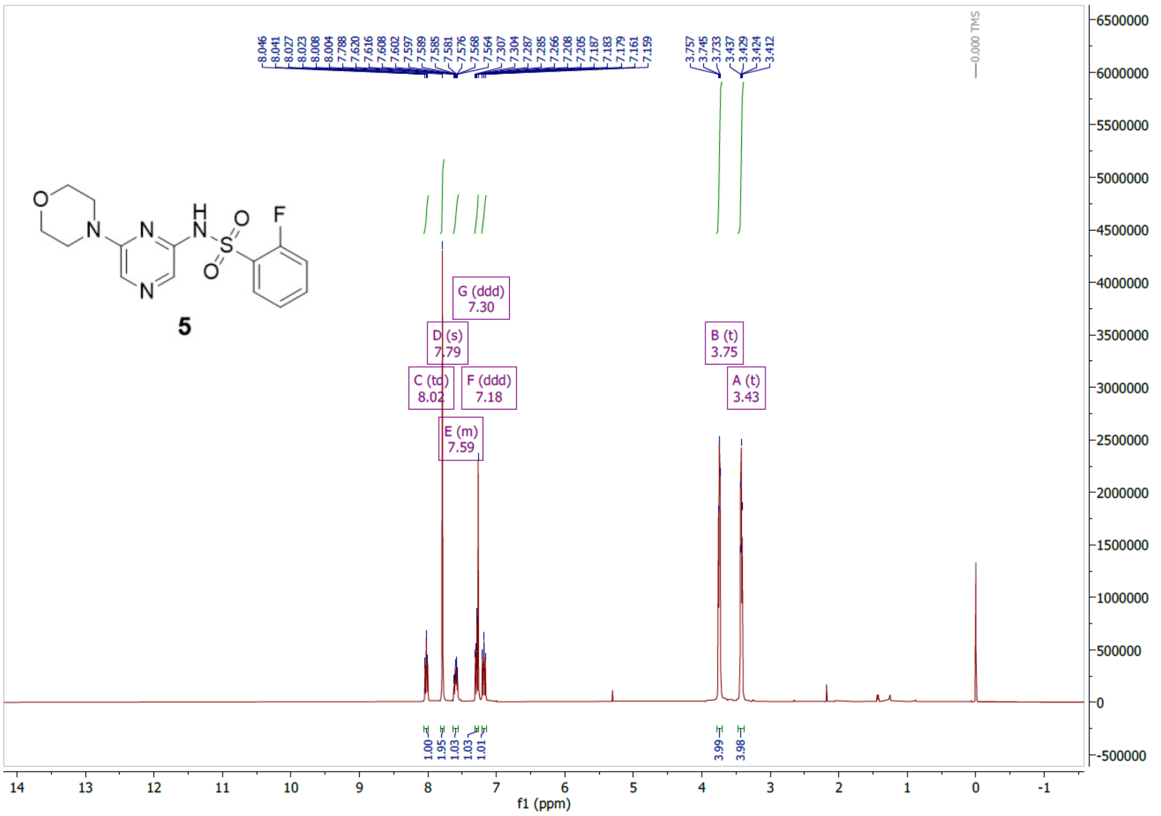
8.046
8.041
8.027
8.023
8.008
8.004
7.788
7.620
7.616
7.608
7.602
7.597
7.589
7.585
7.581
7.576
7.568
7.564
7.307
7.304
7.287
7.285
7.266
7.208
7.205
7.187
7.183
7.179
7.161
7.159
3.757
3.745
3.733
3.437
3.429
3.424
3.412
0.000 TMS
5
G (ddd)
7.30
D (s)
7.79
C (td)
8.02
F (ddd)
7.18
E (m)
7.59
B (t)
3.75
A (t)
3.43
1.00
1.95
1.03
1.03
1.01
3.99
3.98
f1 (ppm)

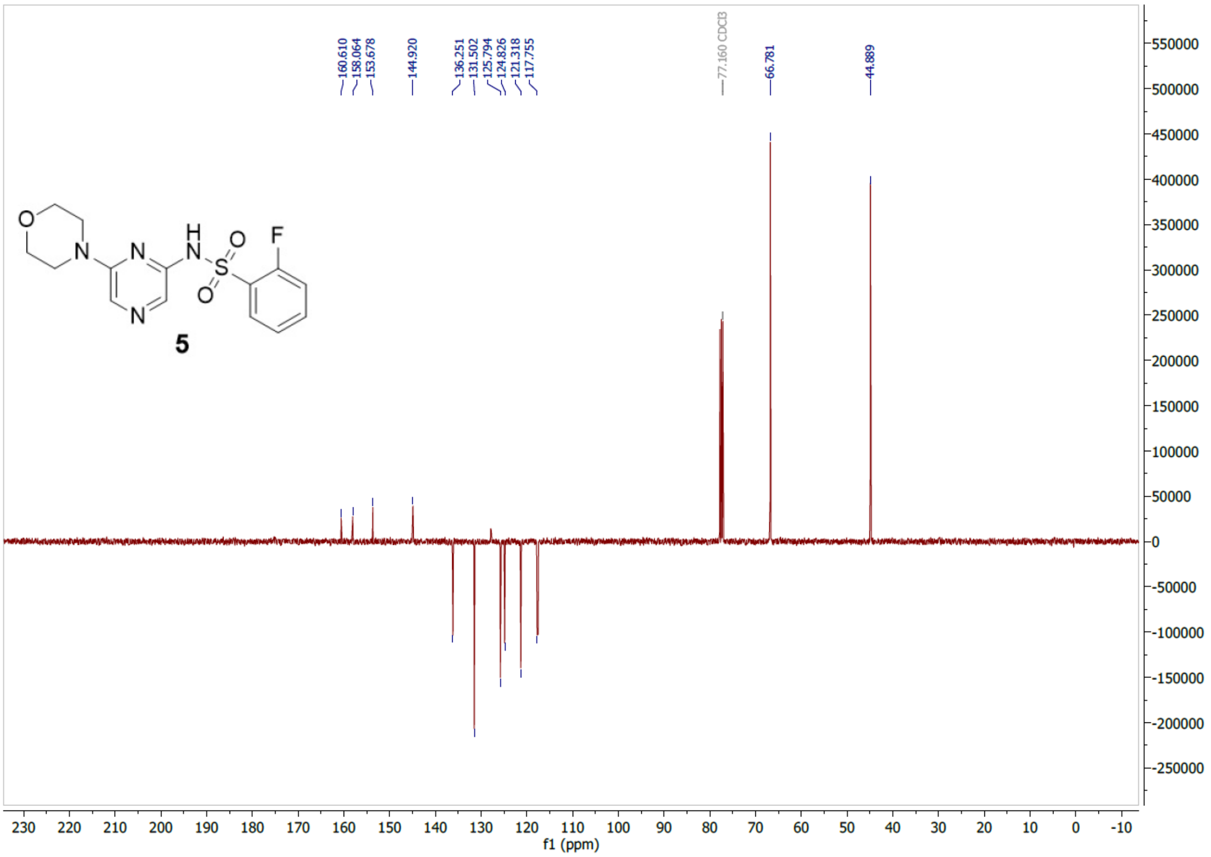
160.610
158.064
153.678
144.920
136.251
131.502
125.794
124.826
121.318
117.755
77.160 CDCl3
66.781
44.889
5
f1 (ppm)

==== Shimadzu LabSolutions Browser Report ====

Chromatogram(KE109-col2-fr10-14.lcd)

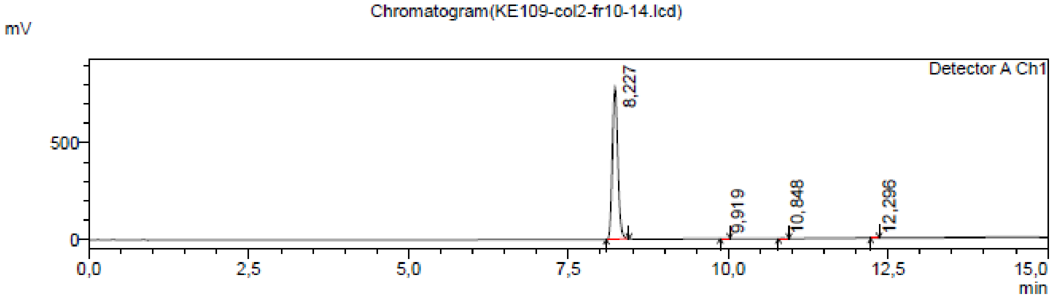

MS Chromatogram(KE109-col2-fr10-14.lcd)

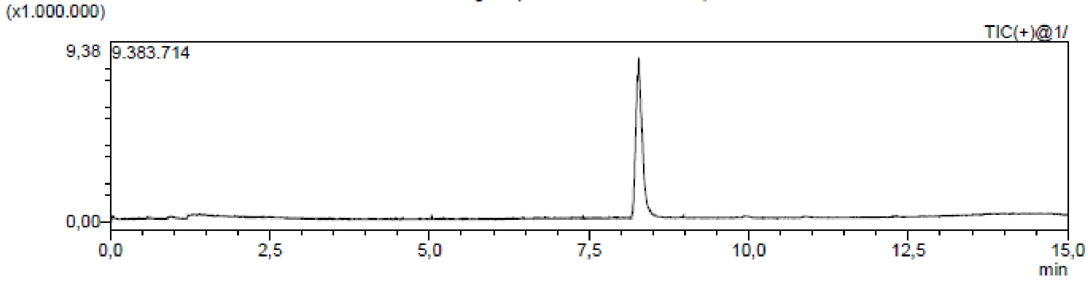

MS Spectrum(KE109-col2-fr10-14.lcd)

Ret. Time: 1-1(E+) [8,255]

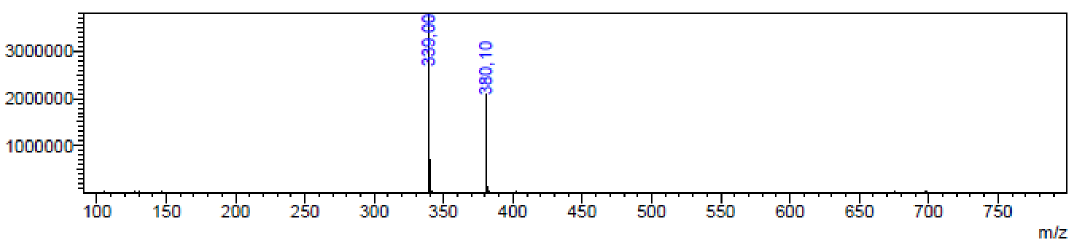

Peak Table(KE109-col2-fr10-14.lcd)

Detector A 254nm

Peak#	Ret. Time	Area	Height	Mark	Conc.	Area%
1	8,227	4659979	781006	M	99,784	99,784
2	9,919	3960	875	M	0,085	0,085
3	10,848	3697	761	M	0,079	0,079
4	12,296	2442	515	M	0,052	0,052
Total		4670078	783158		100,000	100,000

Sample Information(KE109-col2-fr10-14.lcd)

**Data File Name:**	KE109-col2-fr10-14.lcd
**Method File Name:**	MSgeneral1090FA_LowMW.lcm
**Acquired by:**	System Administrator
**Date Acquired:**	9-2-2023 14:06:33
**Sample Name:**	KE109-col2-fr10-14
**Sample ID:**	KE109-col2-fr10-14
**Sample Type:**	Unknown
**Level#:**	0
**Detector:**	Detector A, MS
**Comment:**	

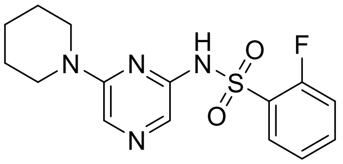

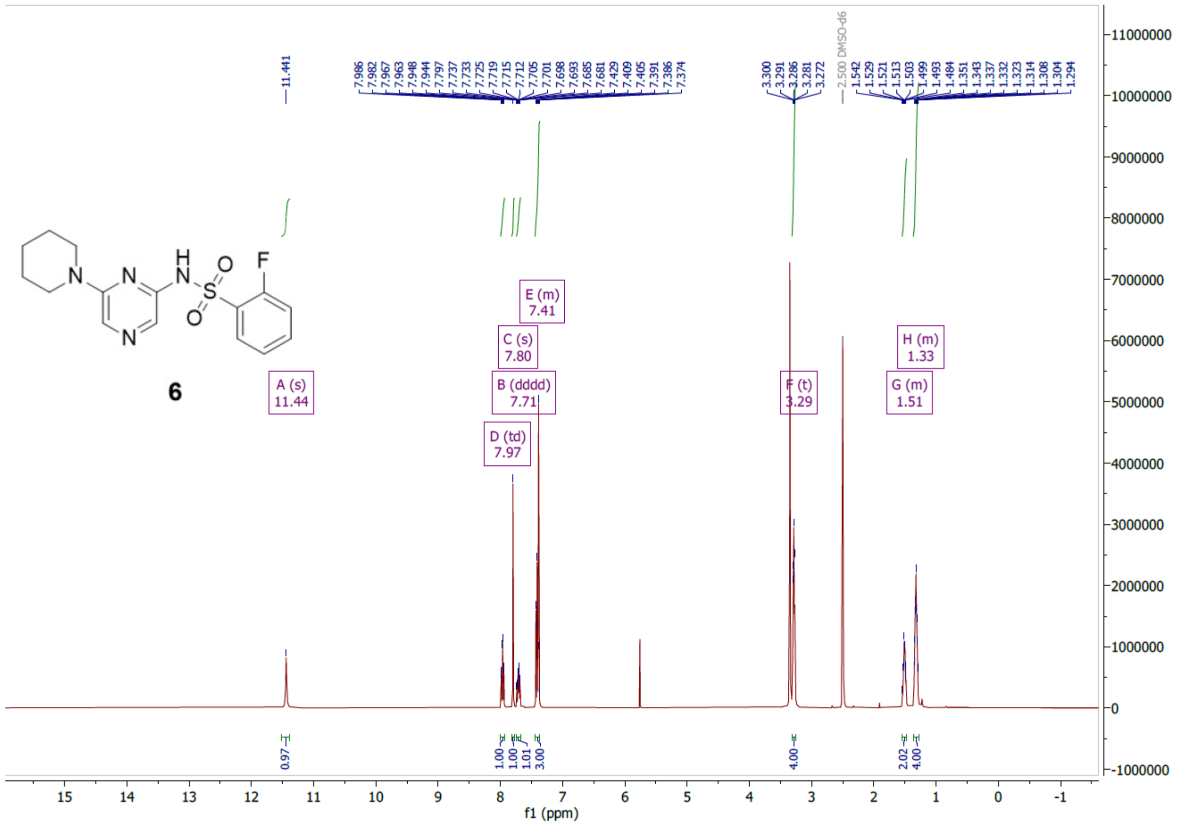

11.441
7.986 7.982 7.967 7.963 7.948 7.944 7.797 7.737 7.733 7.725 7.719 7.715 7.712 7.705 7.701 7.698 7.693 7.685 7.681 7.429 7.409 7.405 7.391 7.386 7.374
3.300 3.291 3.286 3.281 3.272
2.500 DMSO-d6
1.542 1.529 1.521 1.513 1.503 1.499 1.493 1.484 1.351 1.343 1.337 1.332 1.323 1.314 1.308 1.304 1.294
6
A (s) 11.44
B (dddd) 7.71
C (s) 7.80
D (td) 7.97
E (m) 7.41
F (t) 3.29
G (m) 1.51
H (m) 1.33
0.97
1.00
1.00
1.01
3.00
4.00
2.02
4.00
f1 (ppm)

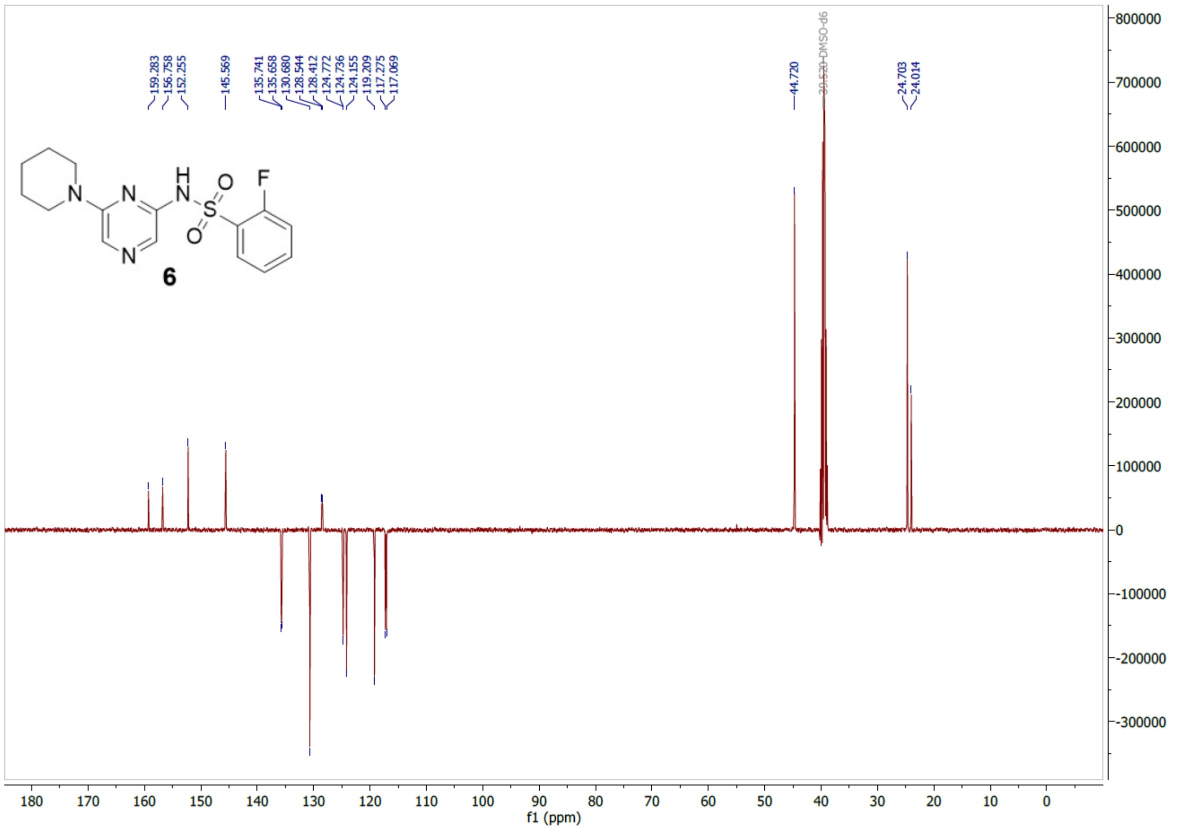

159.283 156.758 152.255
145.569
135.741 135.658 130.680 128.544 128.412 124.772 124.736 124.155 119.209 117.275 117.069
44.720
39.520 DMSO-d6
24.703 24.014
6
f1 (ppm)

==== Shimadzu LabSolutions Browser Report ====

Chromatogram(KE116-pure.lcd)

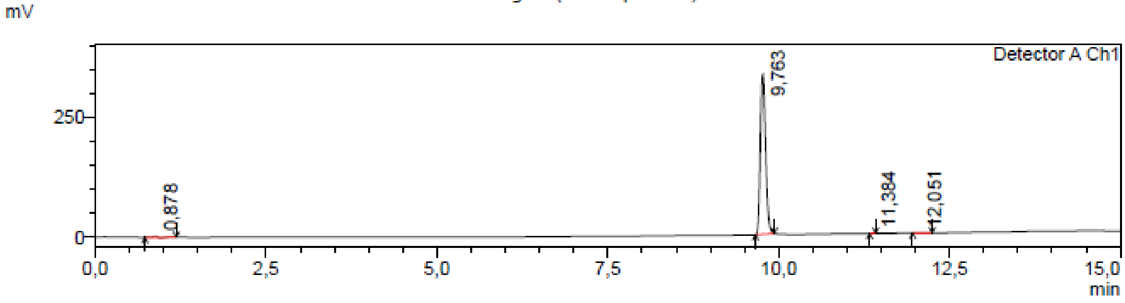

MS Chromatogram(KE116-pure.lcd)

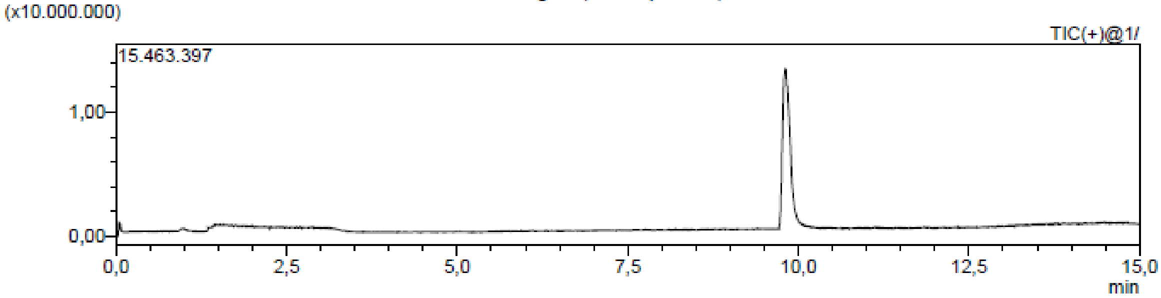

MS Spectrum(KE116-pure.lcd)

Ret. Time: 1-1(E+) [9,800]

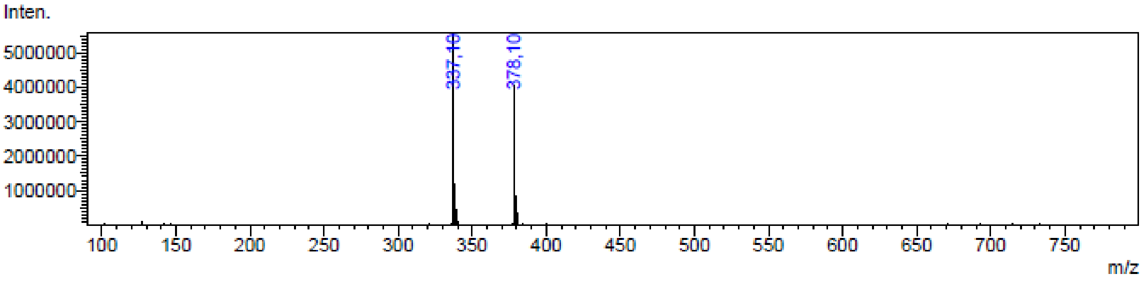

Peak Table(KE116-pure.lcd)

Detector A 254nm

Peak#	Ret. Time	Area	Height	Mark	Conc.	Area%
1	0,878	6472	2520	M	0,361	0,361
2	9,763	1785576	333651	M	99,462	99,462
3	11,384	408	84	M	0,023	0,023
4	12,051	2786	481	M	0,155	0,155
Total		1795242	336736		100,000	100,000

Sample Information(KE116-pure.lcd)

Data File Name:	KE116-pure.lcd
Method File Name:	MSgeneral1090FA_LowMW.lcm
Acquired by:	System Administrator
Date Acquired:	1-3-2023 14:26:17
Sample Name:	KE116-pure
Sample ID:	KE116-pure
Sample Type:	Unknown
Level#:	0
Detector:	Detector A, MS
Comment:	

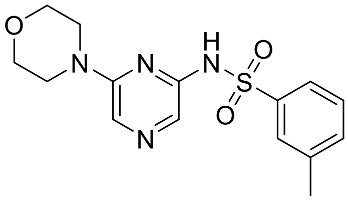

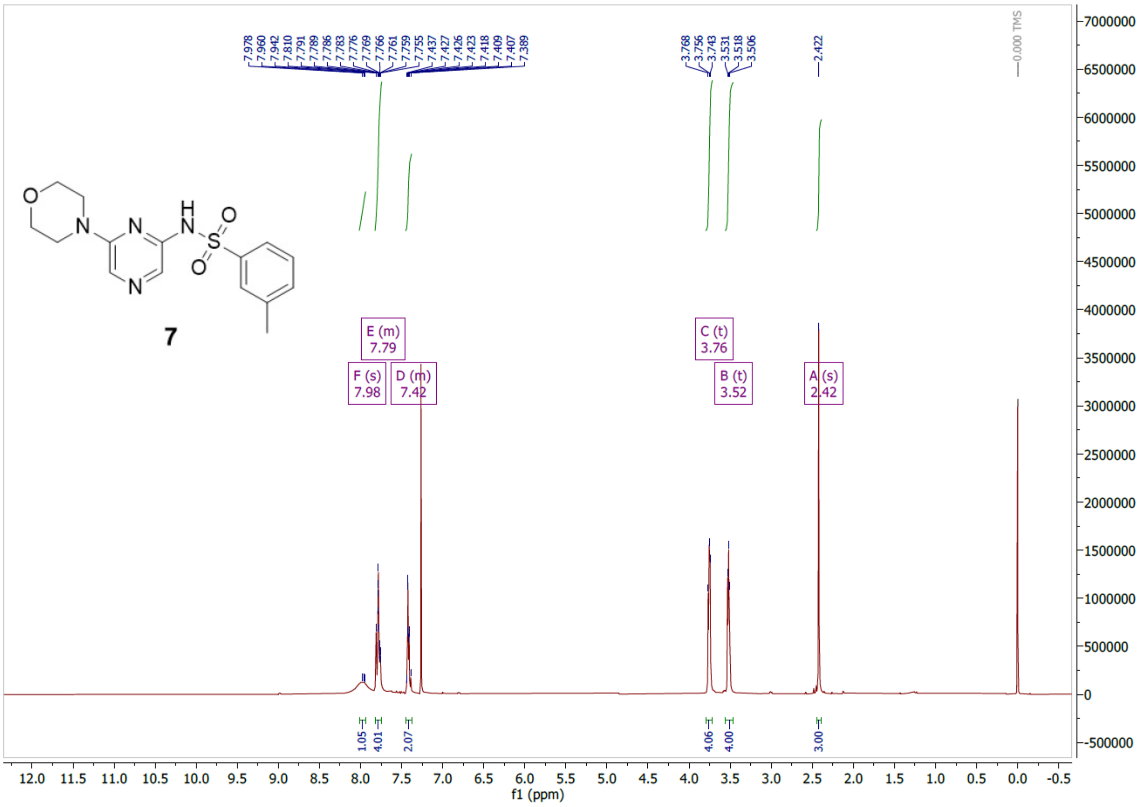

7
F (s) 7.98
E (m) 7.79
D (m) 7.42
C (t) 3.76
B (t) 3.52
A (s) 2.42
0.000 TMS
f1 (ppm)

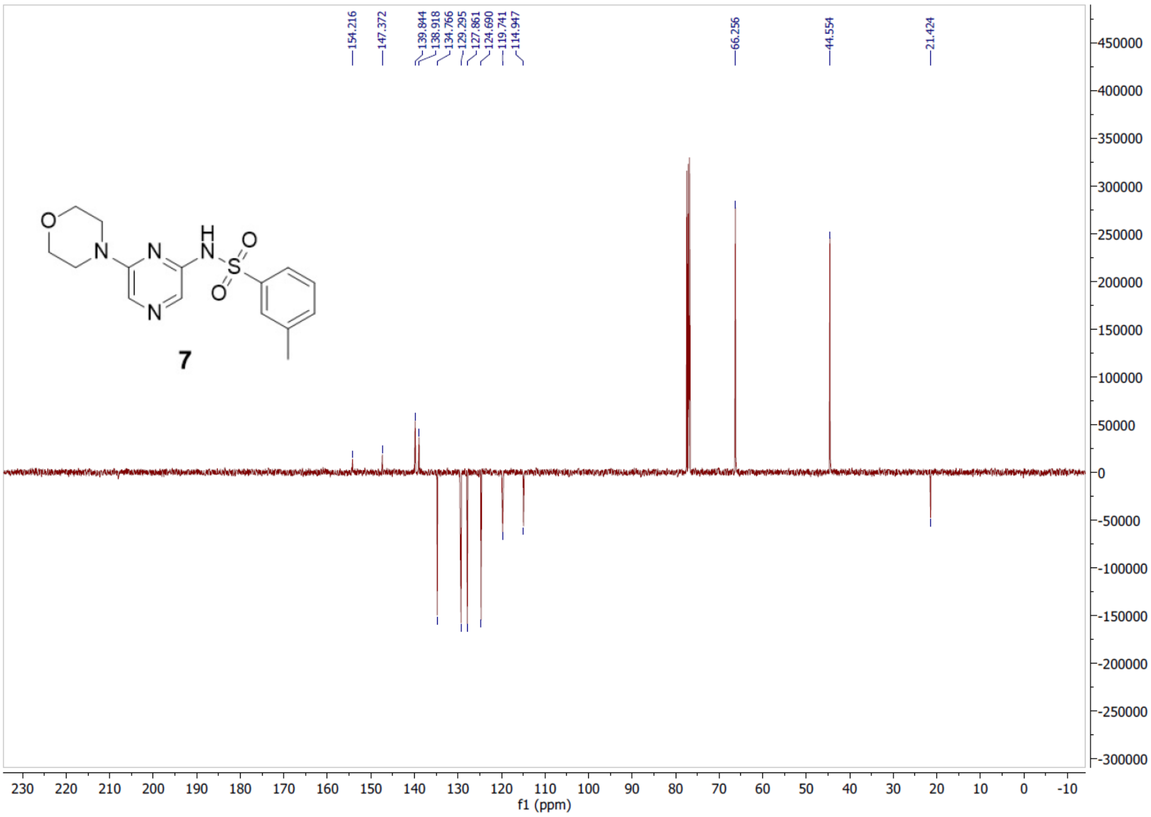

7
154.216
147.372
139.844
138.918
134.766
129.295
127.861
124.690
119.741
114.947
66.256
44.554
21.424
f1 (ppm)

==== Shimadzu LabSolutions Browser Report ====

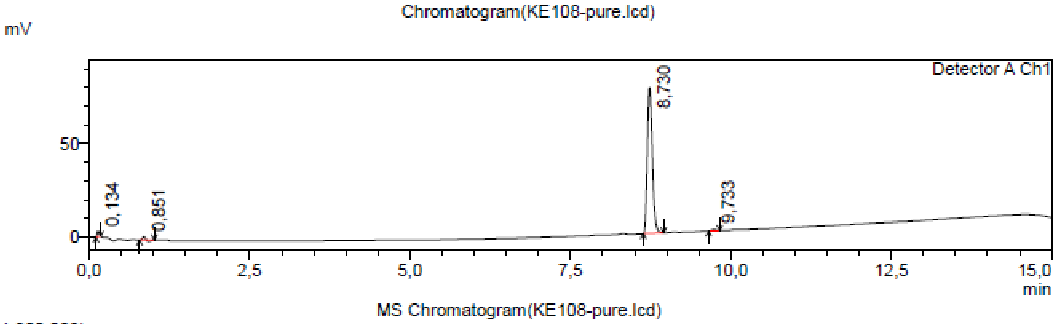

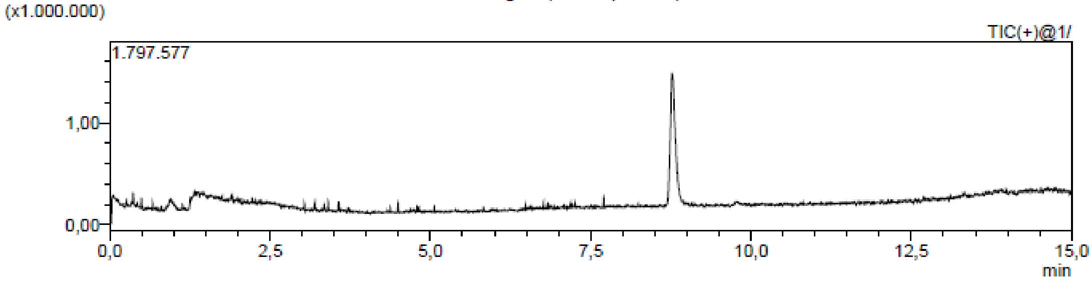

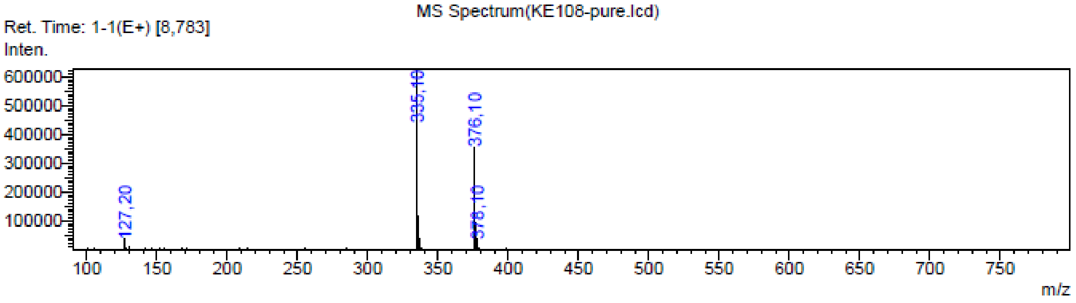

Peak Table(KE108-pure.lcd)

Detector A 254nm

Peak#	Ret. Time	Area	Height	Mark	Conc.	Area%
1	0,134	6767	2881	M	1,553	1,553
2	0,851	2382	1638	M	0,547	0,547
3	8,730	423241	77714	M	97,133	97,133
4	9,733	3343	665	M	0,767	0,767
Total		435734	82898		100,000	100,000

Sample Information(KE108-pure.lcd)

**Data File Name:**	KE108-pure.lcd
**Method File Name:**	MSgeneral1090FA_LowMW.lcm
**Acquired by:**	System Administrator
**Date Acquired:**	9-2-2023 15:13:49
**Sample Name:**	KE108-pure
**Sample ID:**	KE108-pure
**Sample Type:**	Unknown
**Level#:**	0
**Detector:**	Detector A, MS
**Comment:**	

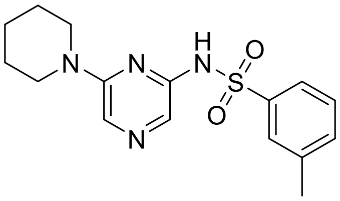

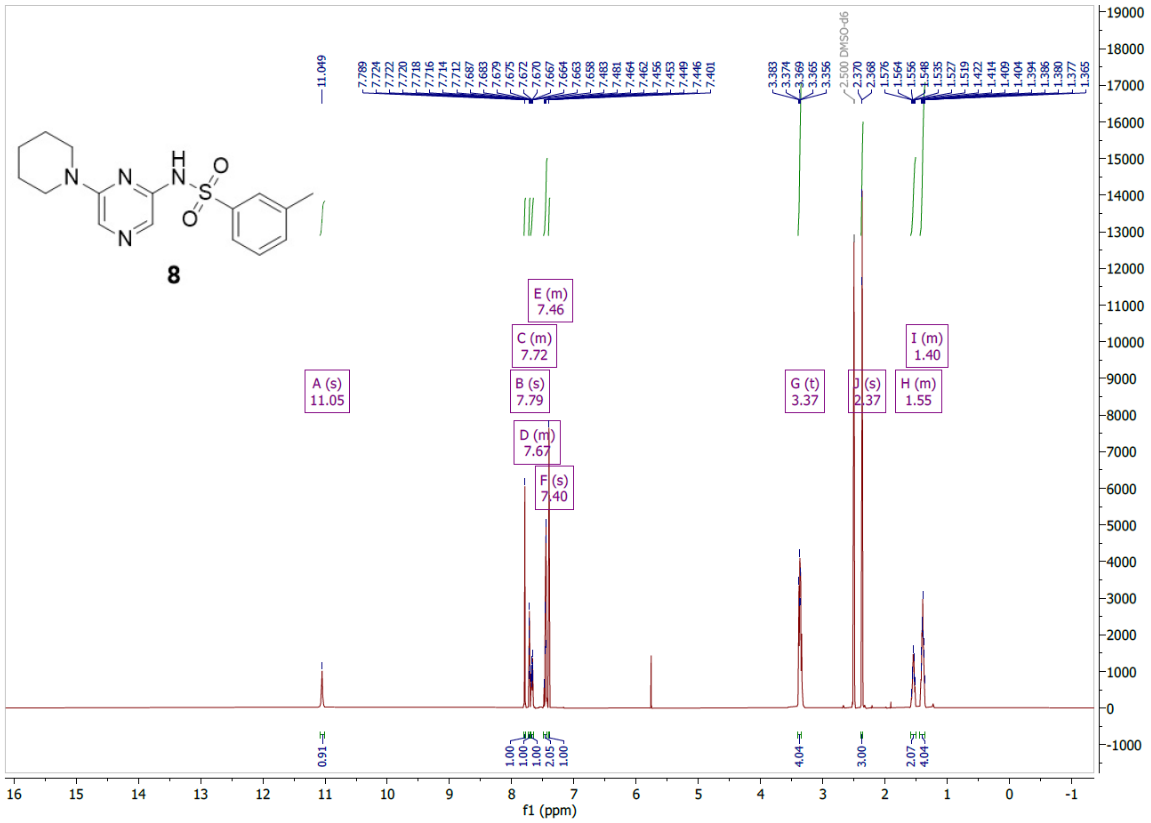

8
11.049
7.789
7.724
7.722
7.720
7.718
7.716
7.714
7.712
7.687
7.683
7.679
7.675
7.672
7.670
7.667
7.664
7.663
7.658
7.483
7.481
7.464
7.462
7.456
7.453
7.449
7.446
7.401
3.383
3.374
3.369
3.365
3.356
2.500 DMSO-d6
2.370
2.368
1.576
1.564
1.556
1.548
1.535
1.527
1.519
1.422
1.414
1.409
1.404
1.394
1.386
1.380
1.377
1.365
A (s) 11.05
B (s) 7.79
C (m) 7.72
D (m) 7.67
E (m) 7.46
F (s) 7.40
G (t) 3.37
J (s) 2.37
H (m) 1.55
I (m) 1.40
0.91
1.00
1.00
1.00
2.05
1.00
4.04
3.00
2.07
4.04
f1 (ppm)

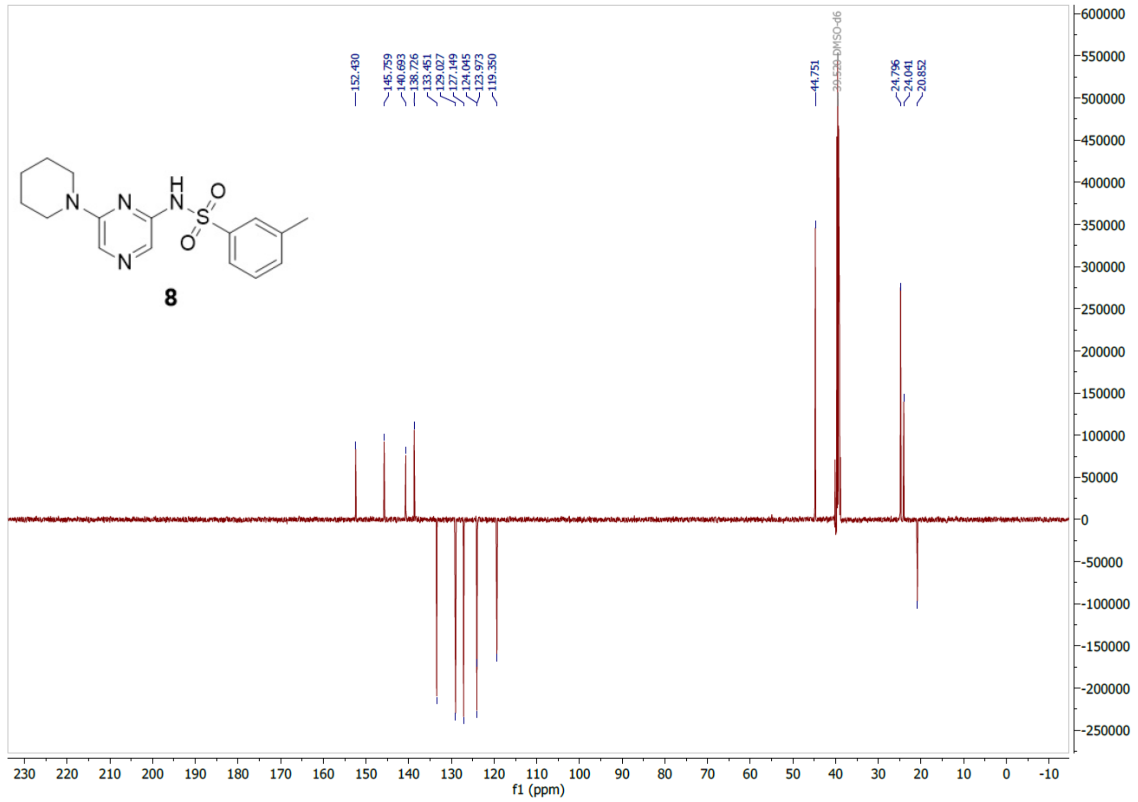

8
152.430
145.759
140.693
138.726
133.451
129.027
127.149
124.045
123.973
119.350
44.751
39.520 DMSO-d6
24.796
24.041
20.852
f1 (ppm)

==== Shimadzu LabSolutions Browser Report ====

Chromatogram(KE113-col3-fr3-13.lcd)

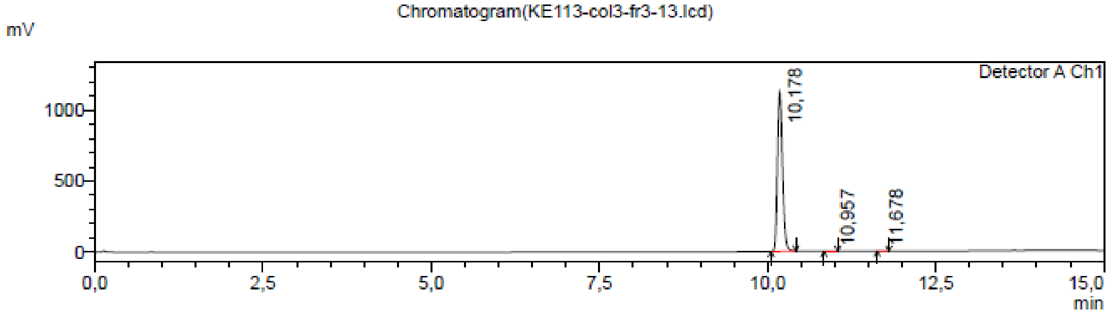

MS Chromatogram(KE113-col3-fr3-13.lcd)

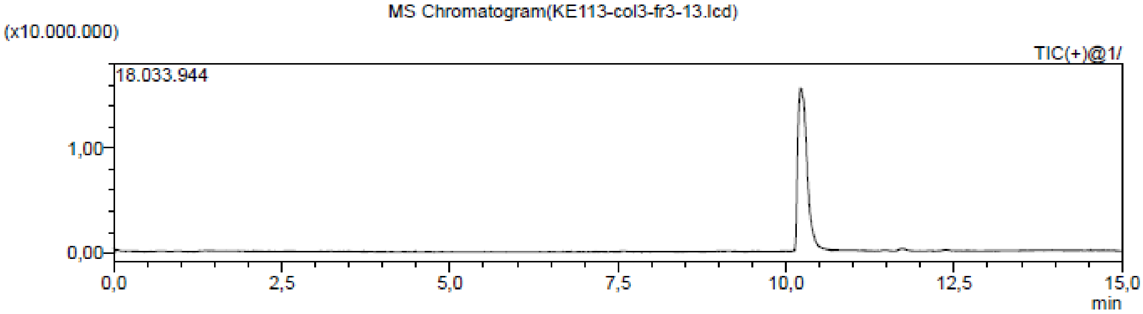

MS Spectrum(KE113-col3-fr3-13.lcd)

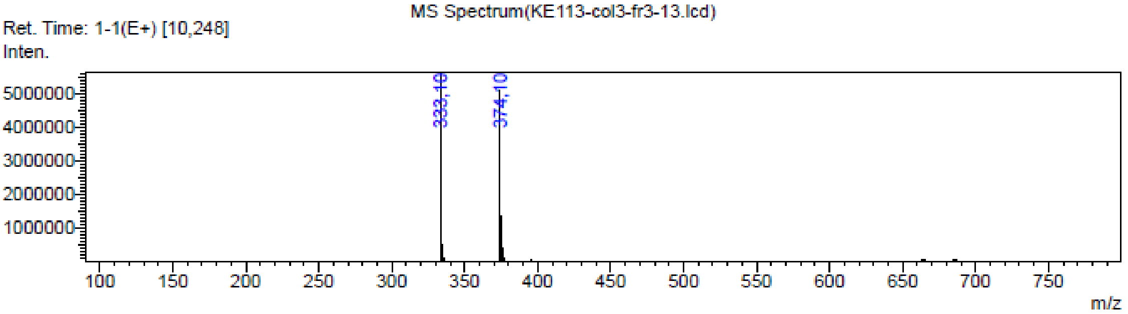

Peak Table(KE113-col3-fr3-13.lcd)

Detector A 254nm

Peak#	Ret. Time	Area	Height	Mark	Conc.	Area%
1	10,178	5967153	1127237	M	99,775	99,775
2	10,957	5583	781	M	0,093	0,093
3	11,678	7881	1434	M	0,132	0,132
Total		5980617	1129451		100,000	100,000

Sample Information(KE113-col3-fr3-13.lcd)

Data File Name: KE113-col3-fr3-13.lcd
Method File Name: MSgeneral1090FA_LowMW.lcm
Acquired by: System Administrator
Date Acquired: 21-2-2023 16:08:20
Sample Name: KE113-col3-fr3-13
Sample ID: KE113-col3-fr3-13
Sample Type: Unknown
Level#: 0
Detector: Detector A, MS
Comment:

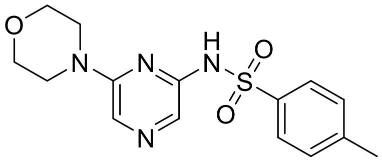

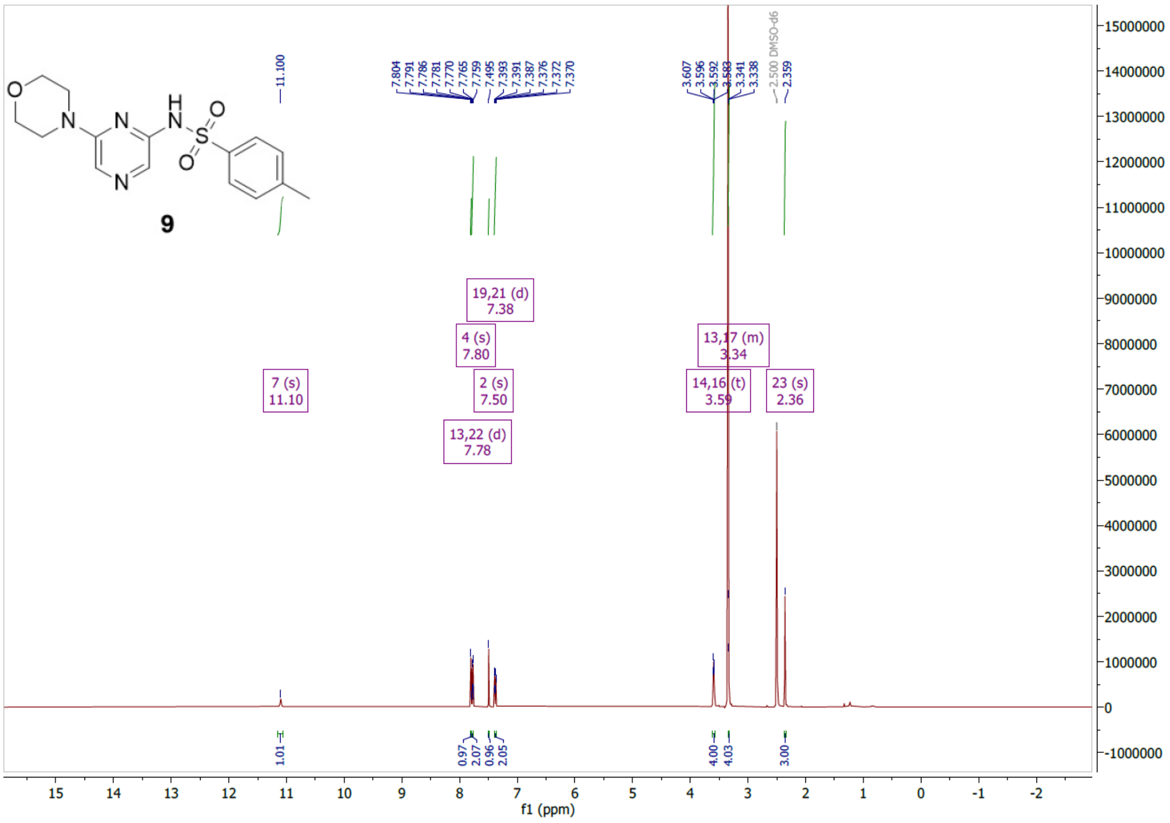

9
11.100
7.804 7.791 7.786 7.781 7.770 7.765 7.759 7.495 7.393 7.391 7.387 7.376 7.372 7.370
3.607 3.596 3.592 3.583 3.341 3.338
2.500 DMSO-d6
2.359
19,21 (d) 7.38
4 (s) 7.80
13,17 (m) 3.34
7 (s) 11.10
2 (s) 7.50
14,16 (t) 3.59
23 (s) 2.36
13,22 (d) 7.78
1.01
0.97 2.07 0.96 2.05
4.00 4.03
3.00
f1 (ppm)

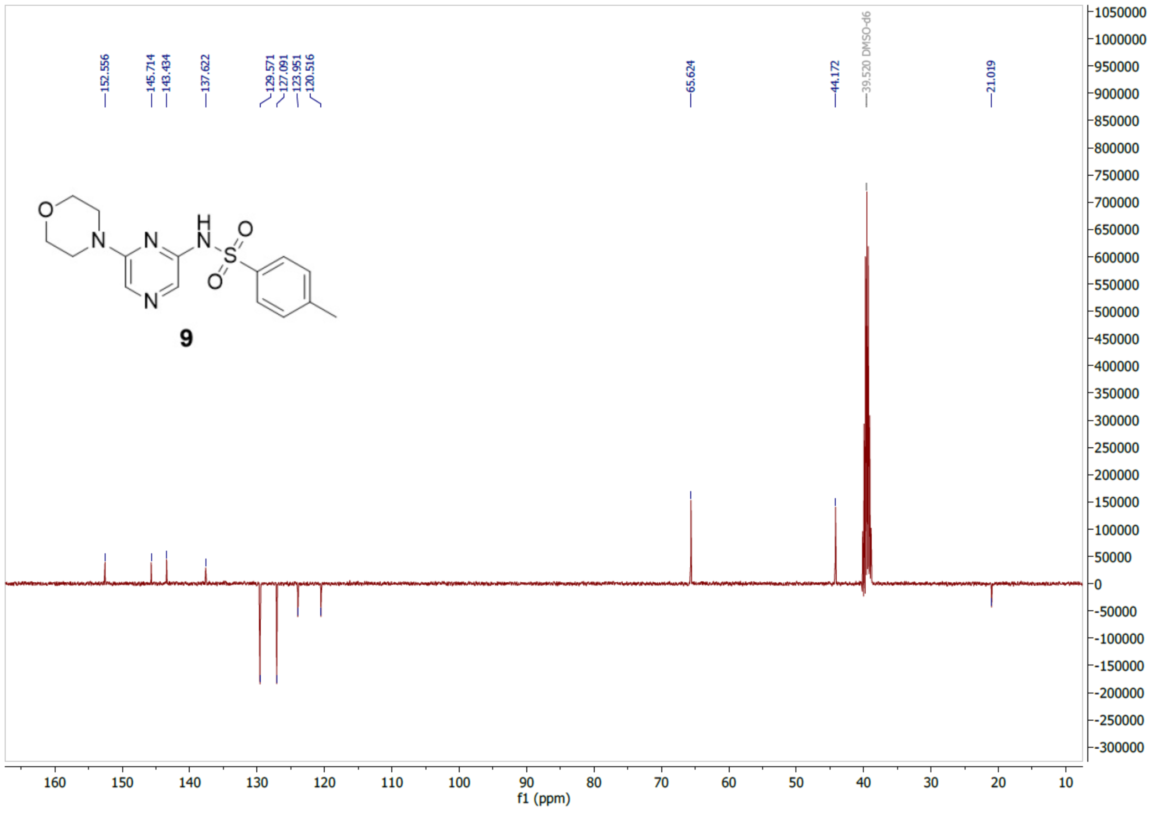

152.556
145.714 143.434
137.622
129.571 127.091 123.951 120.516
65.624
44.172
39.520 DMSO-d6
21.019
9
f1 (ppm)

==== Shimadzu LabSolutions Browser Report ====

Chromatogram(KN_05_pure.lcd)

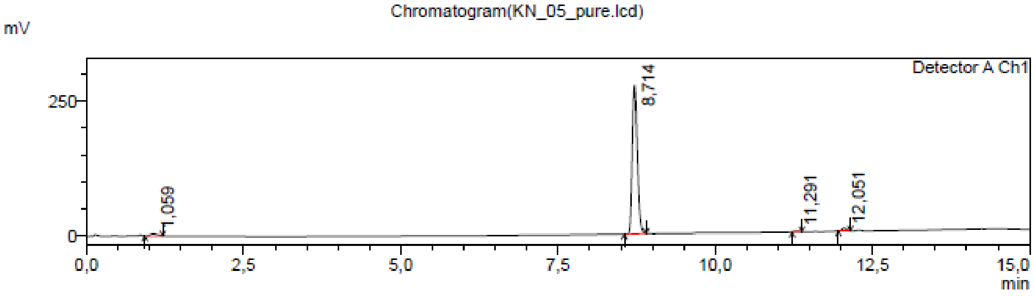

MS Chromatogram(KN_05_pure.lcd)

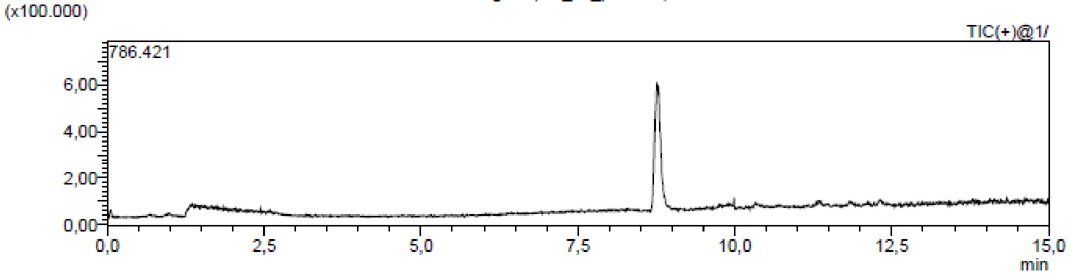

MS Spectrum(KN_05_pure.lcd)

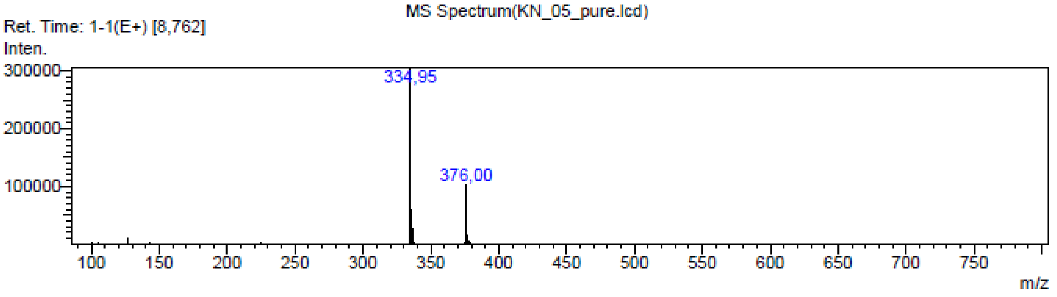

Peak Table(KN_05_pure.lcd)

Detector A 254nm

Peak#	Ret. Time	Area	Height	Mark	Conc.	Area%
1	1,059	34208	4294	M	2,171	2,171
2	8,714	1507499	273802	M	95,689	95,689
3	11,291	8026	1722	M	0,509	0,509
4	12,051	25676	5212	M	1,630	1,630
Total		1575408	285030		100,000	100,000

Sample Information(KN_05_pure.lcd)

**Data File Name:**	KN_05_pure.lcd
**Method File Name:**	MSgeneral1090FA_LowMW.lcm
**Acquired by:**	System Administrator
**Date Acquired:**	25-1-2023 14:40:30
**Sample Name:**	KN_05_pure
**Sample ID:**	KN_05_pure
**Sample Type:**	Unknown
**Level#:**	0
**Detector:**	Detector A, MS
**Comment:**	

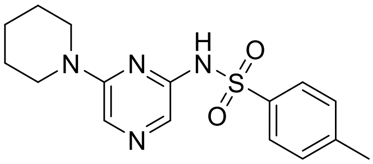

N
N
N
H
N
S
O
O

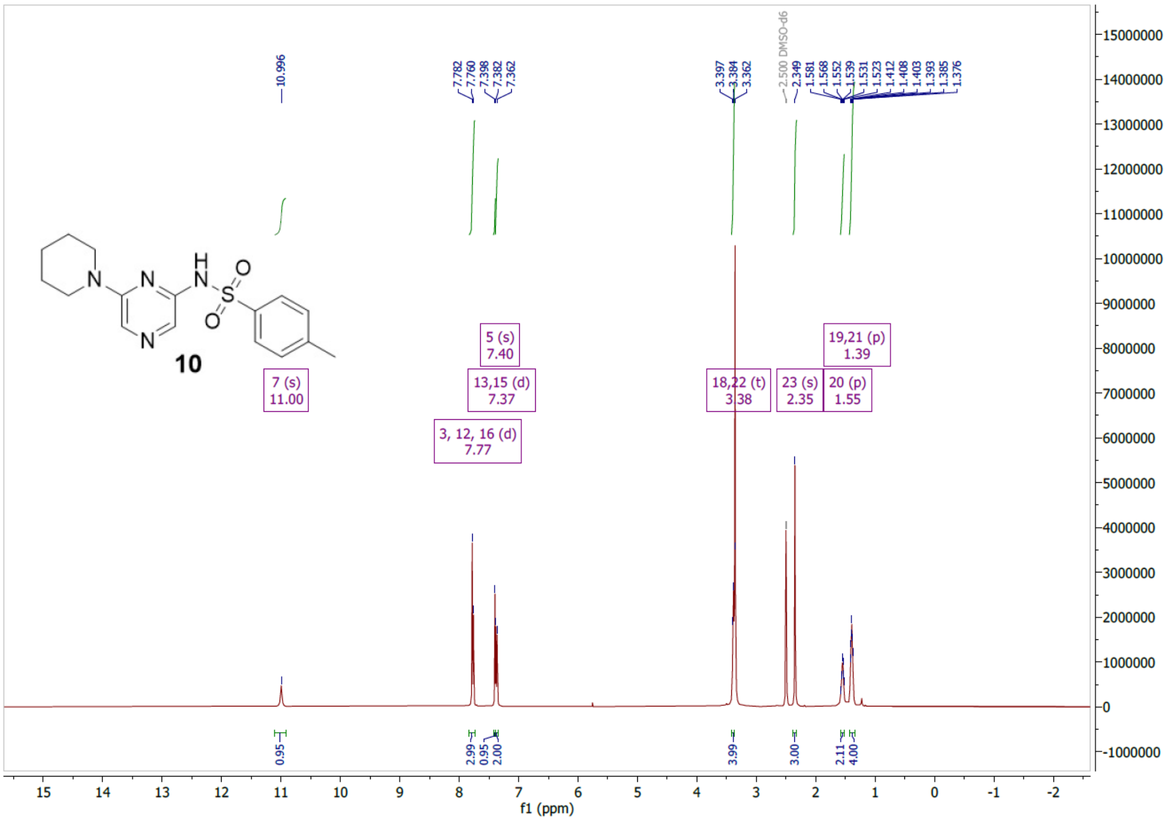

10.996
7.782
7.760
7.398
7.382
7.362
3.397
3.384
3.362
2.500 DMSO-d6
2.349
1.581
1.568
1.552
1.539
1.531
1.523
1.412
1.408
1.403
1.393
1.385
1.376
10
7 (s)
11.00
5 (s)
7.40
13,15 (d)
7.37
3, 12, 16 (d)
7.77
18,22 (t)
3.38
23 (s)
2.35
19,21 (p)
1.39
20 (p)
1.55
0.95
2.99
0.95
2.00
3.99
3.00
2.11
4.00
f1 (ppm)

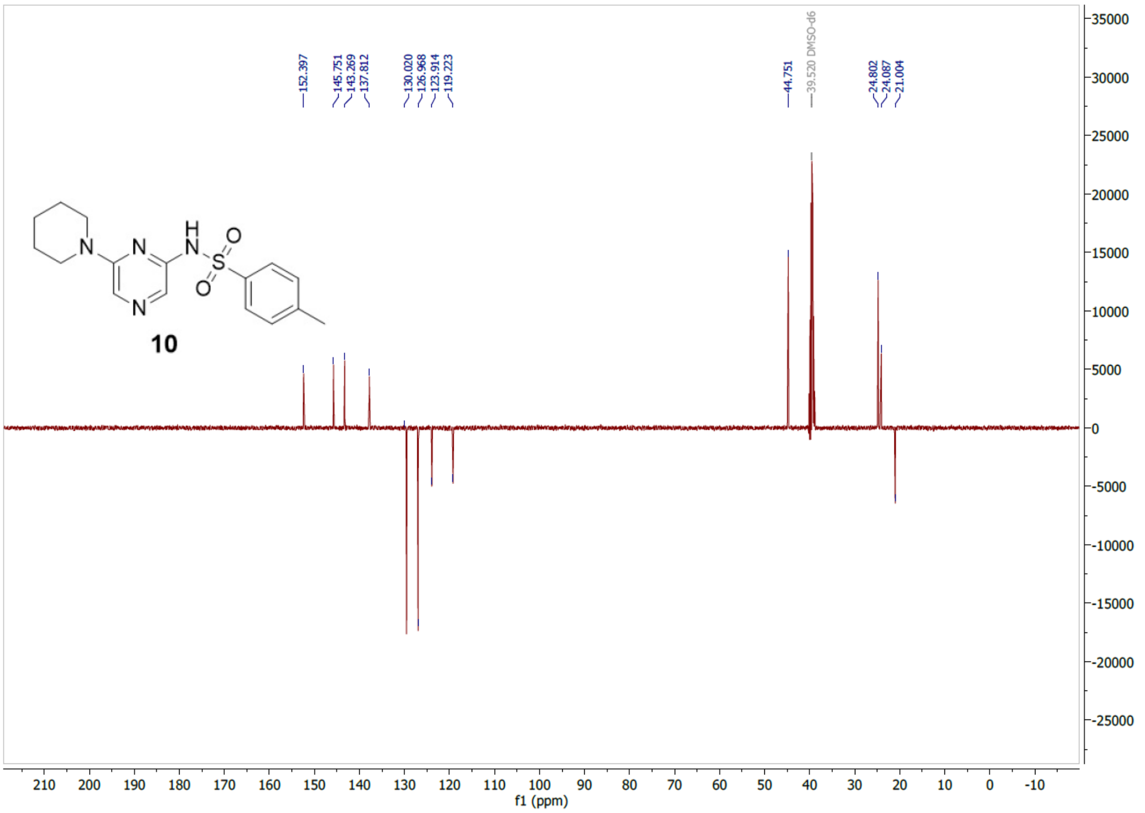

152.397
145.751
143.269
137.812
130.020
126.968
123.914
119.223
44.751
39.520 DMSO-d6
24.802
24.087
21.004
10
f1 (ppm)

==== Shimadzu LabSolutions Browser Report ====

Chromatogram(KN018_pure.lcd)

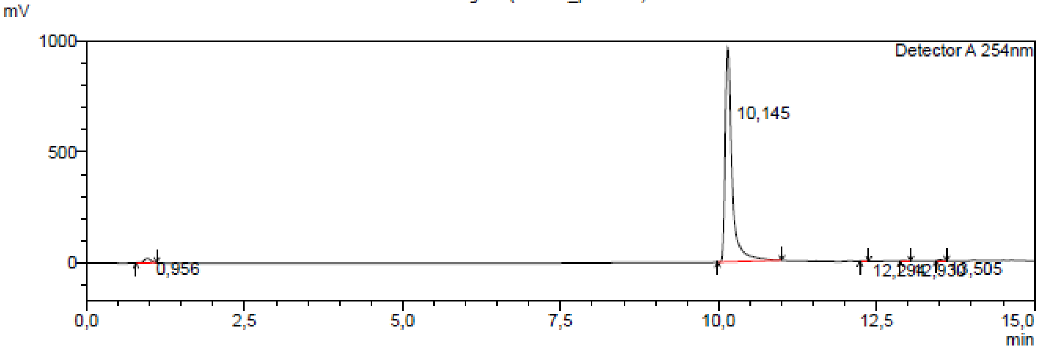

MS Chromatogram(KN018_pure.lcd)

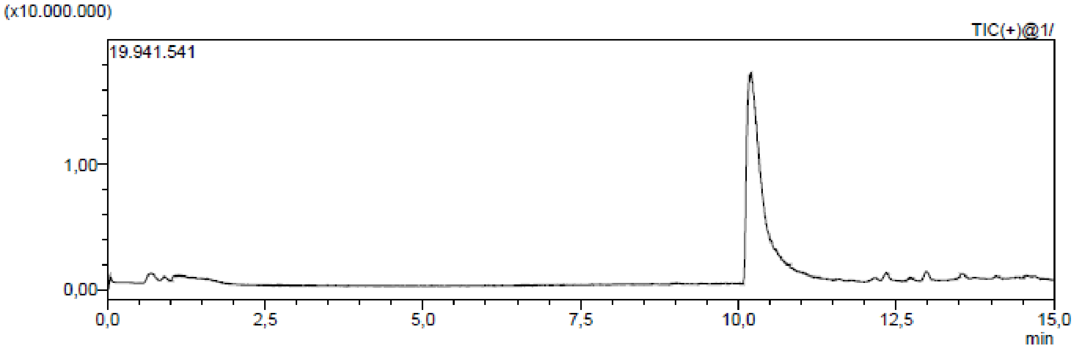

MS Spectrum(KN018_pure.lcd)

Ret. Time: 1-1(E+) [10,215]

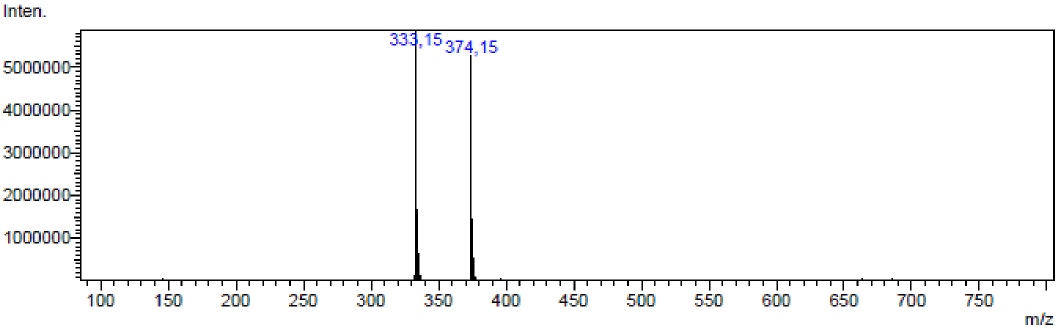

Peak Table(KN018_pure.lcd)

Detector A 254nm

Peak#	Ret. Time	Area	Height	Mark	Conc.	Area%
1	0,956	148043	19573	M	1,968	1,968
2	10,145	7335474	967441	M	97,494	97,494
3	12,294	7385	1641	M	0,098	0,098
4	12,930	12088	2628	M	0,161	0,161
5	13,505	21023	4358	M	0,279	0,279
Total		7524013	995640		100,000	100,000

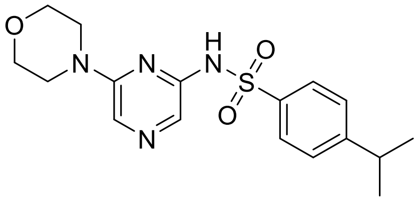

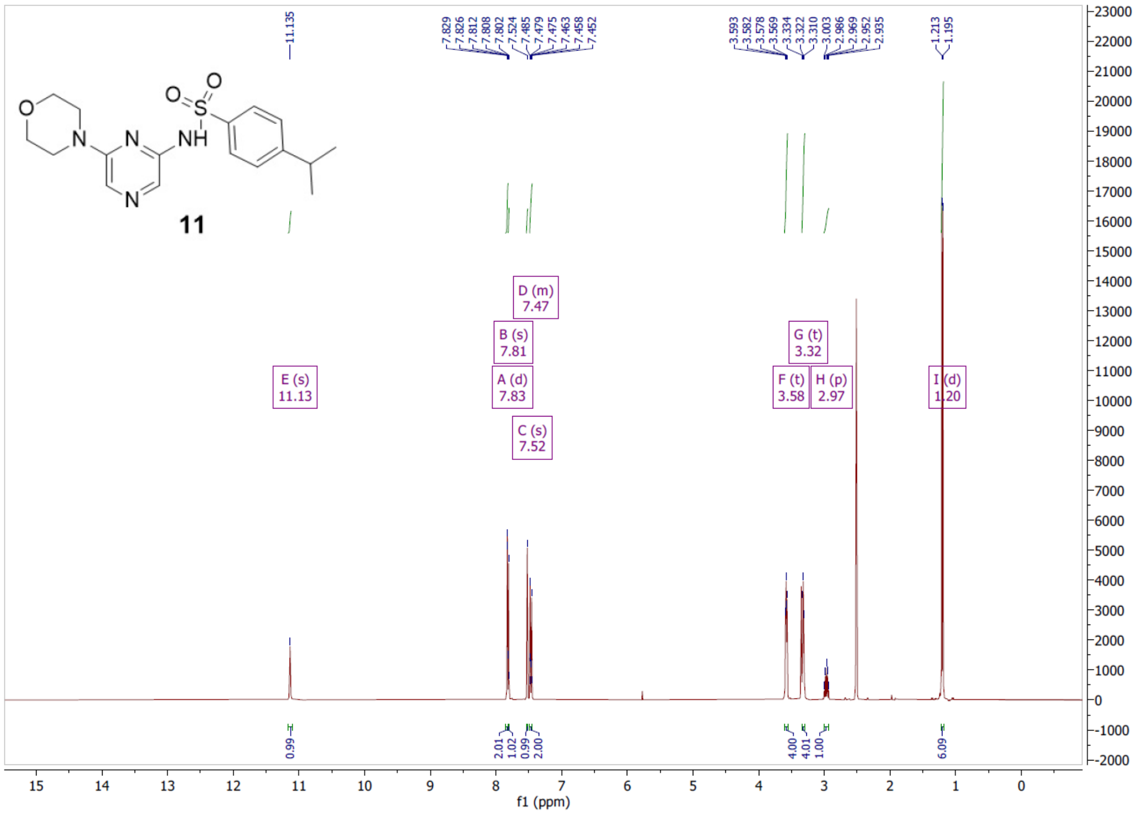
11
E (s)
11.13
A (d)
7.83
B (s)
7.81
C (s)
7.52
D (m)
7.47
F (t)
3.58
G (t)
3.32
H (p)
2.97
I (d)
1.20
f1 (ppm)

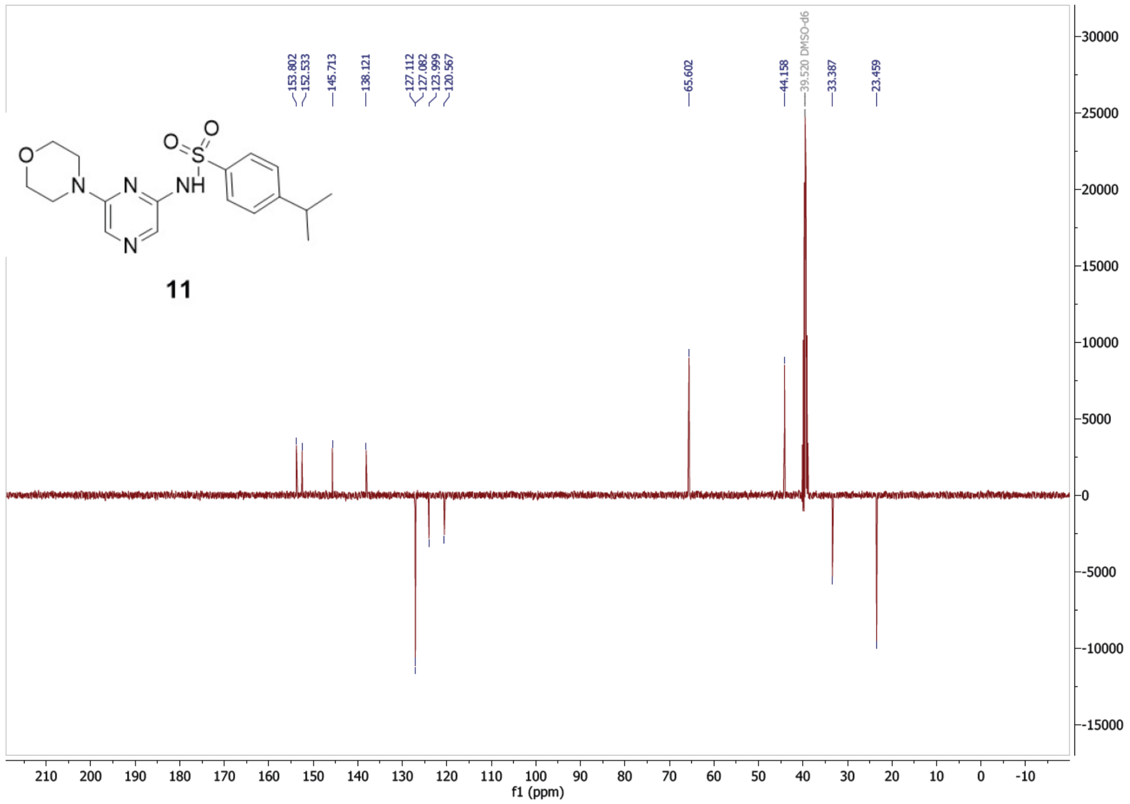
11
153.802
152.533
145.713
138.121
127.112
127.082
123.999
120.567
65.602
44.158
39.520 DMSO-d6
33.387
23.459
f1 (ppm)

==== Shimadzu LabSolutions Browser Report ====

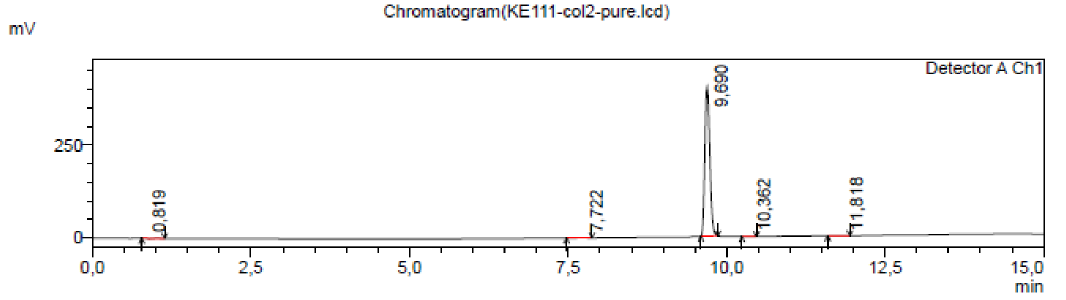

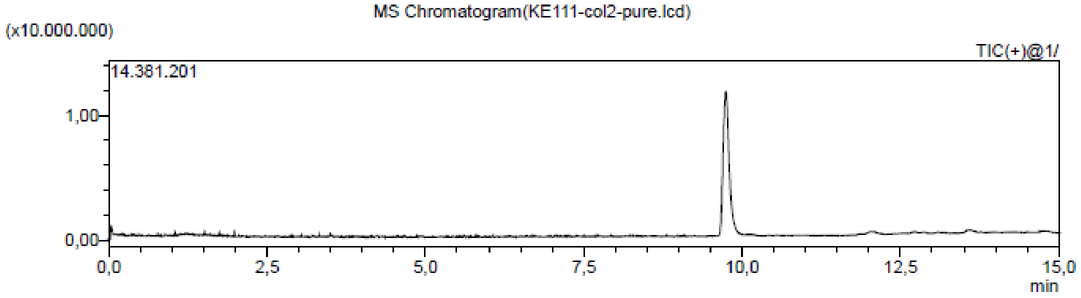

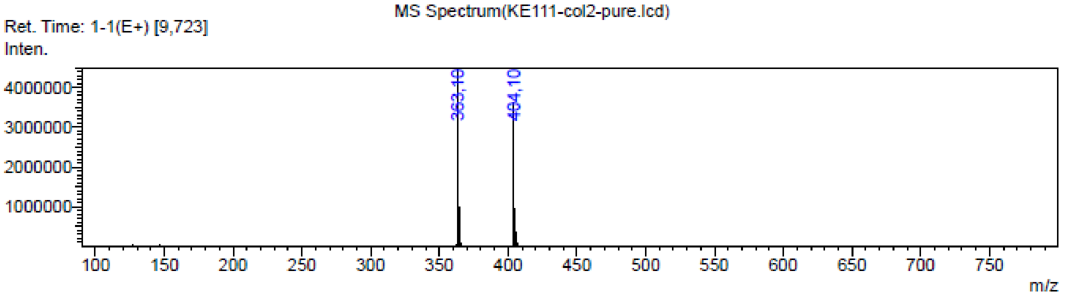

Peak Table(KE111-col2-pure.lcd)

Detector A 254nm

Peak#	Ret. Time	Area	Height	Mark	Conc.	Area%
1	0,819	350	587	M	0,016	0,016
2	7,722	8579	1026	M	0,396	0,396
3	9,690	2150683	400838	M	99,371	99,371
4	10,362	935	214	M	0,043	0,043
5	11,818	3746	508	M	0,173	0,173
Total		2164292	403173		100,000	100,000

Sample Information(KE111-col2-pure.lcd)

Data File Name:	KE111-col2-pure.lcd
Method File Name:	MSgeneral1090FA_LowMW.lcm
Acquired by:	System Administrator
Date Acquired:	15-2-2023 12:08:20
Sample Name:	KE111-col2-pure
Sample ID:	KE111-col2-pure
Sample Type:	Unknown
Level#:	0
Detector:	Detector A, MS
Comment:	

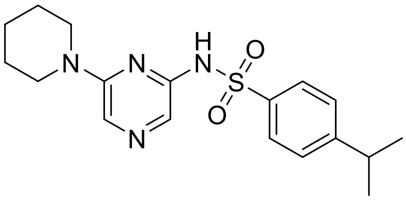

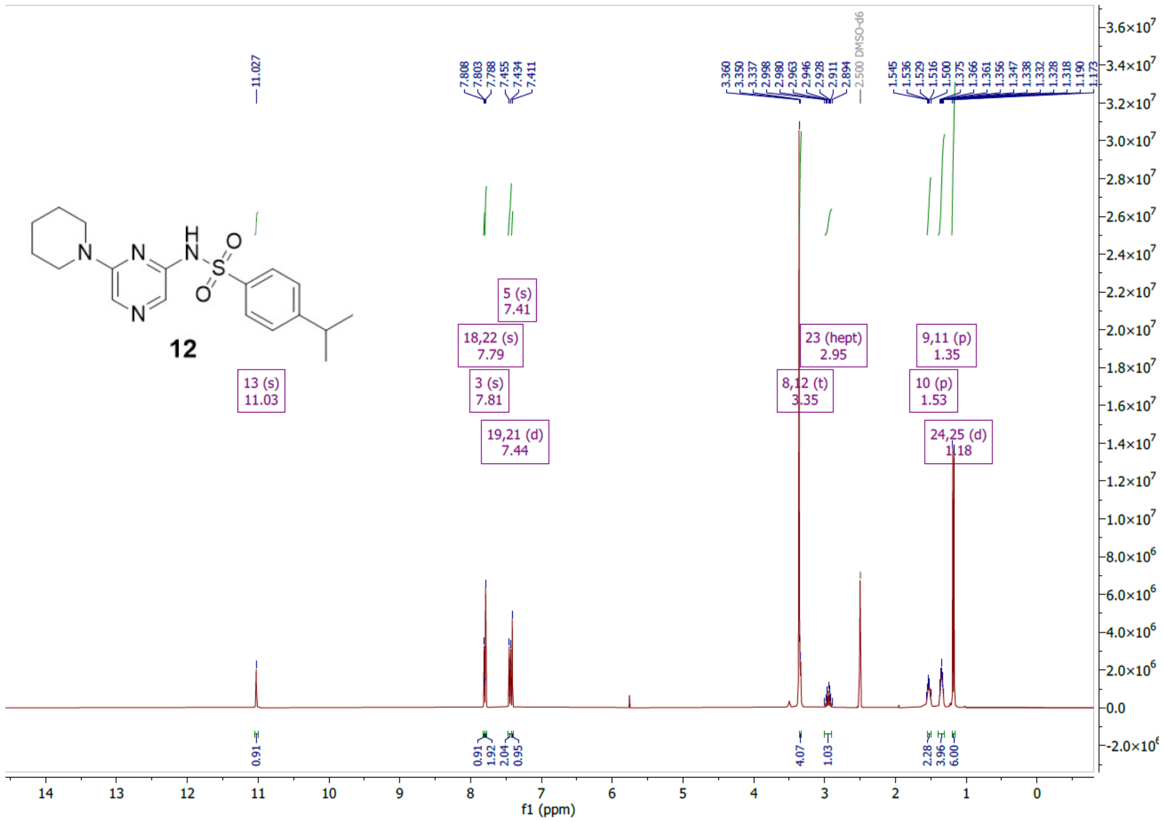
11.027
7.808
7.803
7.788
7.455
7.434
7.411
3.360
3.350
3.337
2.998
2.980
2.963
2.946
2.928
2.911
2.894
2.500 DMSO-d6
1.545
1.536
1.529
1.516
1.500
1.375
1.366
1.361
1.356
1.347
1.338
1.332
1.328
1.318
1.190
1.173
12
5 (s)
7.41
18,22 (s)
7.79
3 (s)
7.81
19,21 (d)
7.44
13 (s)
11.03
23 (hept)
2.95
8,12 (t)
3.35
9,11 (p)
1.35
10 (p)
1.53
24,25 (d)
1.18
0.91
0.91
1.92
2.04
0.95
4.07
1.03
2.28
3.96
6.00
f1 (ppm)

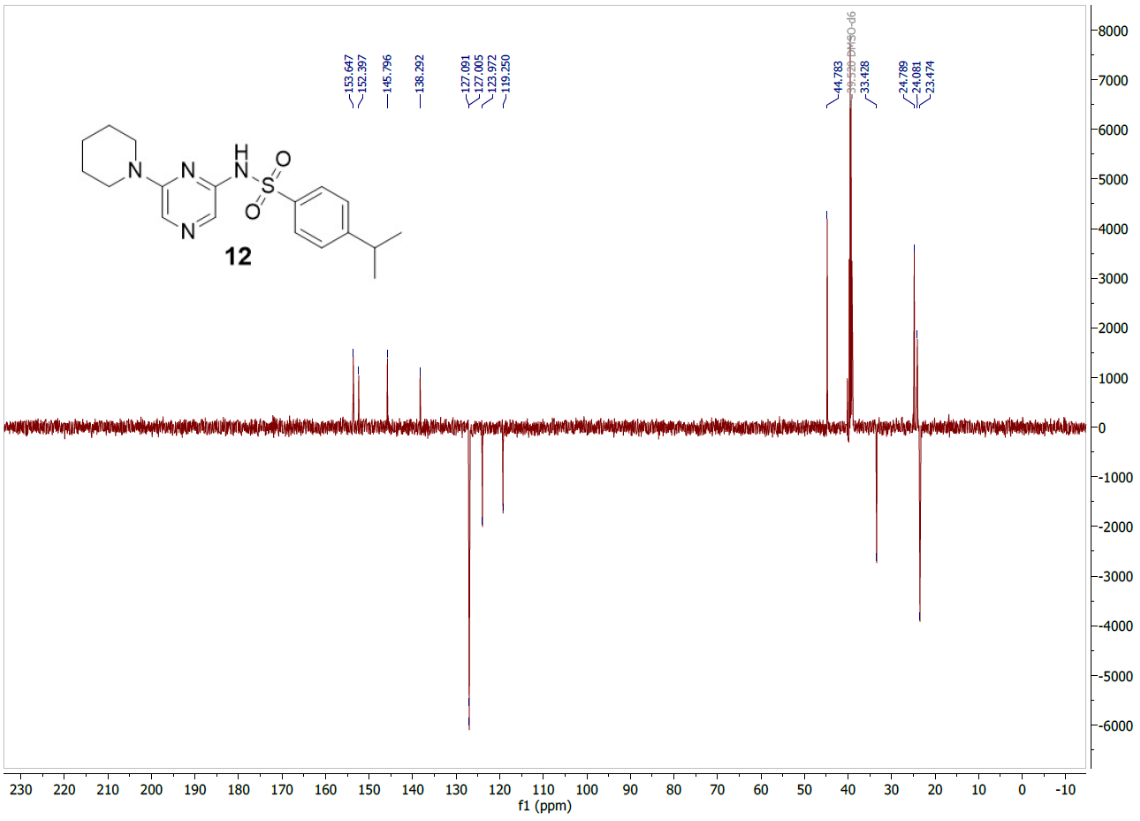
153.647
152.397
145.796
138.292
127.091
127.005
123.972
119.250
44.783
39.520 DMSO-d6
33.428
24.789
24.081
23.474
12
f1 (ppm)

==== Shimadzu LabSolutions Browser Report ====

Chromatogram(KN024_pure.lcd)

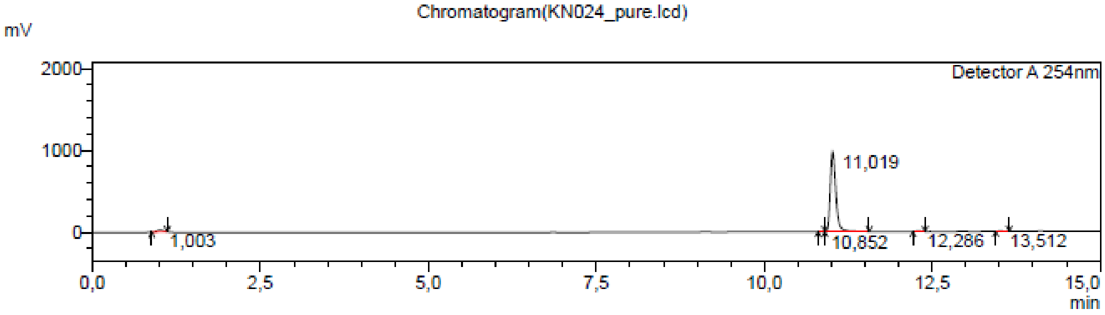

MS Chromatogram(KN024_pure.lcd)

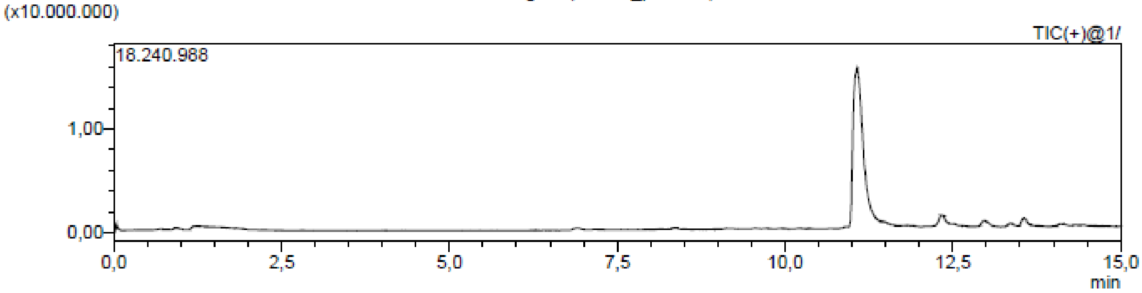

MS Spectrum(KN024_pure.lcd)

Ret. Time: 1-1(E+) [11,055]

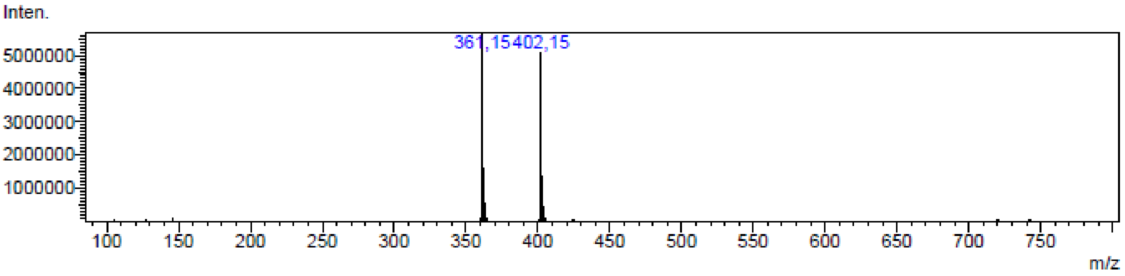

Peak Table(KN024_pure.lcd)

Detector A 254nm

Peak#	Ret. Time	Area	Height	Mark	Conc.	Area%
1	1,003	158656	23473	M	2,701	2,701
2	10,852	9044	2178	M	0,154	0,154
3	11,019	5649442	969816	V M	96,171	96,171
4	12,286	35619	6557	M	0,606	0,606
5	13,512	21616	4231	M	0,368	0,368
Total		5874377	1006256		100,000	100,000

Sample Information(KN024_pure.lcd)

**Data File Name:**	KN024_pure.lcd
**Method File Name:**	MSgeneral1090FA_LowMW.lcm
**Acquired by:**	System Administrator
**Date Acquired:**	21-3-2023 14:50:27
**Sample Name:**	KN024_pure
**Sample ID:**	KN024_pure
**Sample Type:**	Unknown
**Level#:**	0
**Detector:**	Detector A, MS
**Comment:**	

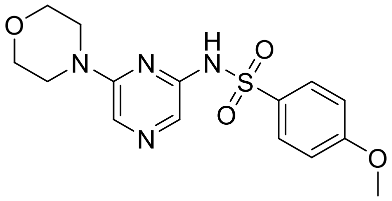

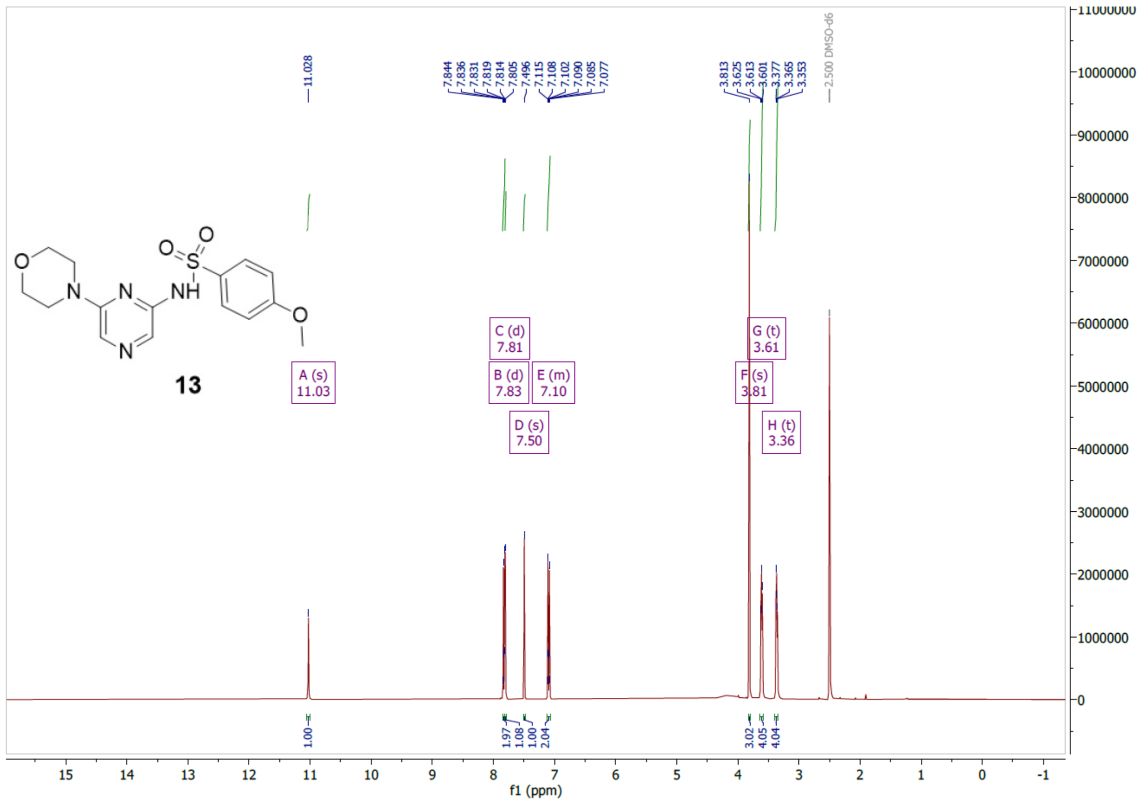
11.028
7.844
7.836
7.831
7.819
7.814
7.805
7.496
7.115
7.108
7.102
7.090
7.085
7.077
3.813
3.625
3.613
3.601
3.377
3.365
3.353
2.500 DMSO-d6
13
A (s)
11.03
C (d)
7.81
B (d)
7.83
E (m)
7.10
D (s)
7.50
G (t)
3.61
F (s)
3.81
H (t)
3.36
1.00
1.97
1.08
1.00
2.04
3.02
4.05
4.04
f1 (ppm)

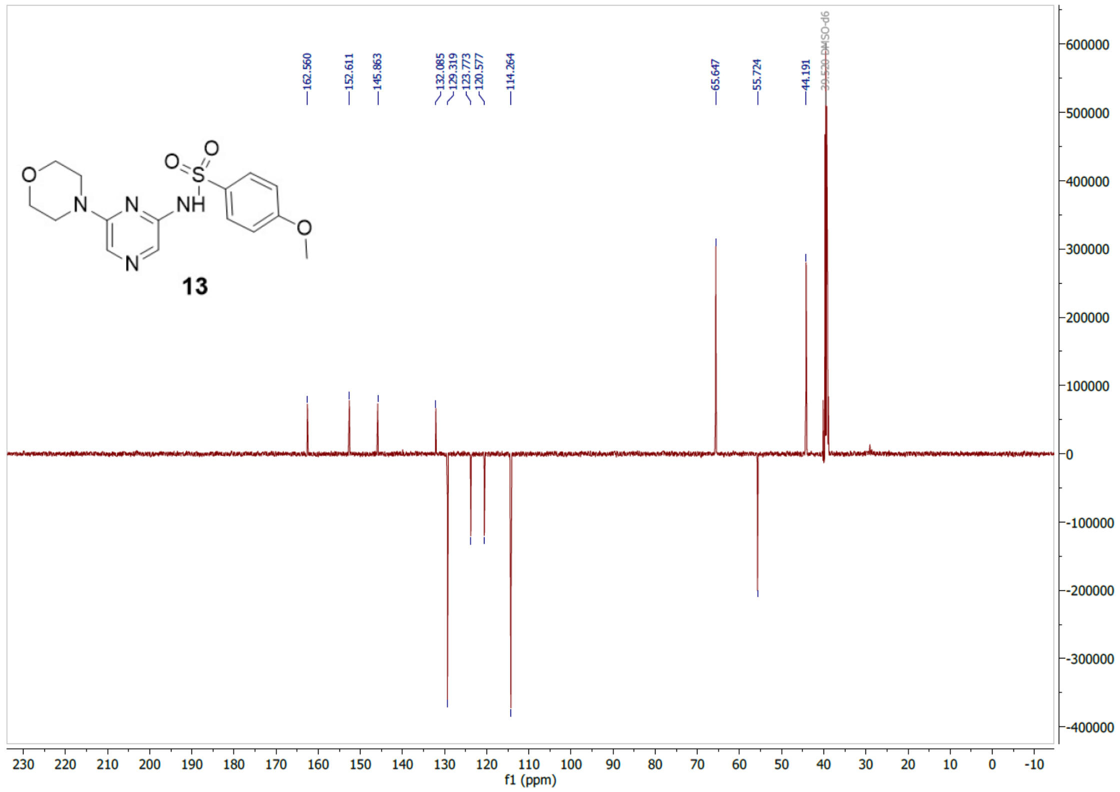
162.560
152.611
145.863
132.085
129.319
123.773
120.577
114.264
65.647
55.724
44.191
39.520 DMSO-d6
13
f1 (ppm)

==== Shimadzu LabSolutions Browser Report ====

Chromatogram(KE112-pure.lcd)

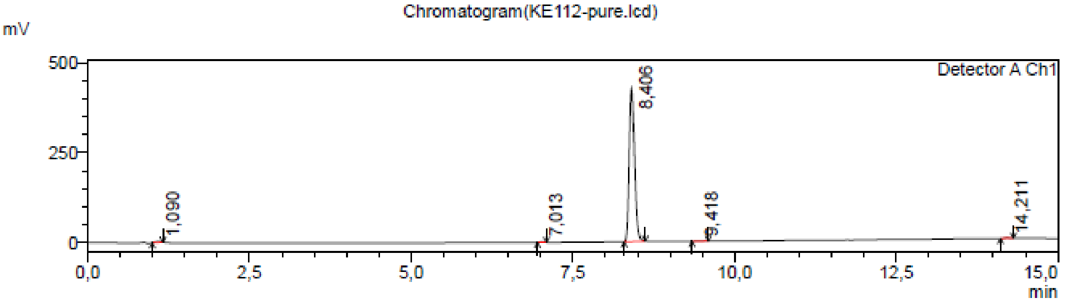

MS Chromatogram(KE112-pure.lcd)

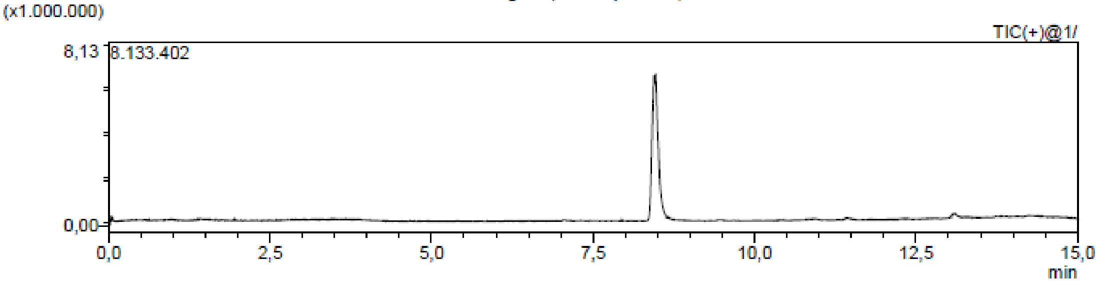

MS Spectrum(KE112-pure.lcd)

Ret. Time: 1-1(E+) [8,438]

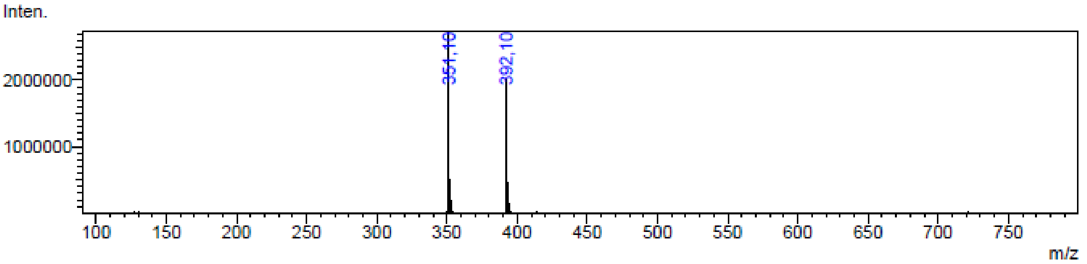

Peak Table(KE112-pure.lcd)

Detector A 254nm

Peak#	Ret. Time	Area	Height	Mark	Conc.	Area%
1	1,090	17252	2680	M	0,711	0,711
2	7,013	5119	1101	M	0,211	0,211
3	8,406	2398066	425154	M	98,787	98,787
4	9,418	2454	481	M	0,101	0,101
5	14,211	4632	884	M	0,191	0,191
Total		2427523	430300		100,000	100,000

Sample Information(KE112-pure.lcd)

**Data File Name:**	**KE112-pure.lcd**
**Method File Name:**	**MSgeneral1090FA_LowMW.lcm**
**Acquired by:**	**System Administrator**
**Date Acquired:**	**17-2-2023 12:20:46**
**Sample Name:**	**KE112-pure**
**Sample ID:**	**KE112-pure**
**Sample Type:**	**Unknown**
**Level#:**	**0**
**Detector:**	**Detector A, MS**
**Comment:**	

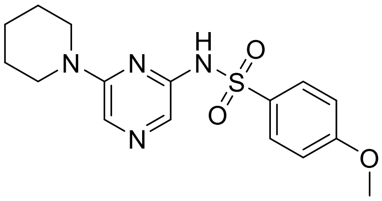

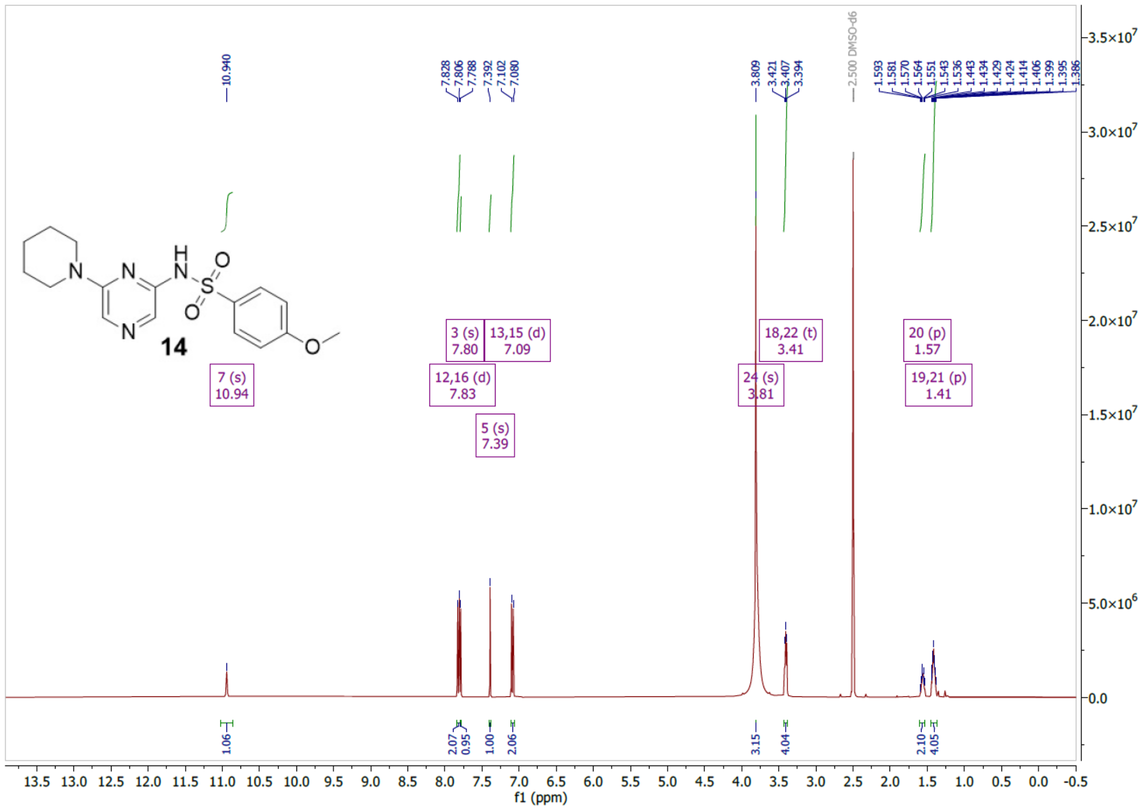
14
7 (s)
10.94
3 (s)
7.80
13,15 (d)
7.09
12,16 (d)
7.83
5 (s)
7.39
18,22 (t)
3.41
24 (s)
3.81
20 (p)
1.57
19,21 (p)
1.41
2.500 DMSO-d6
f1 (ppm)

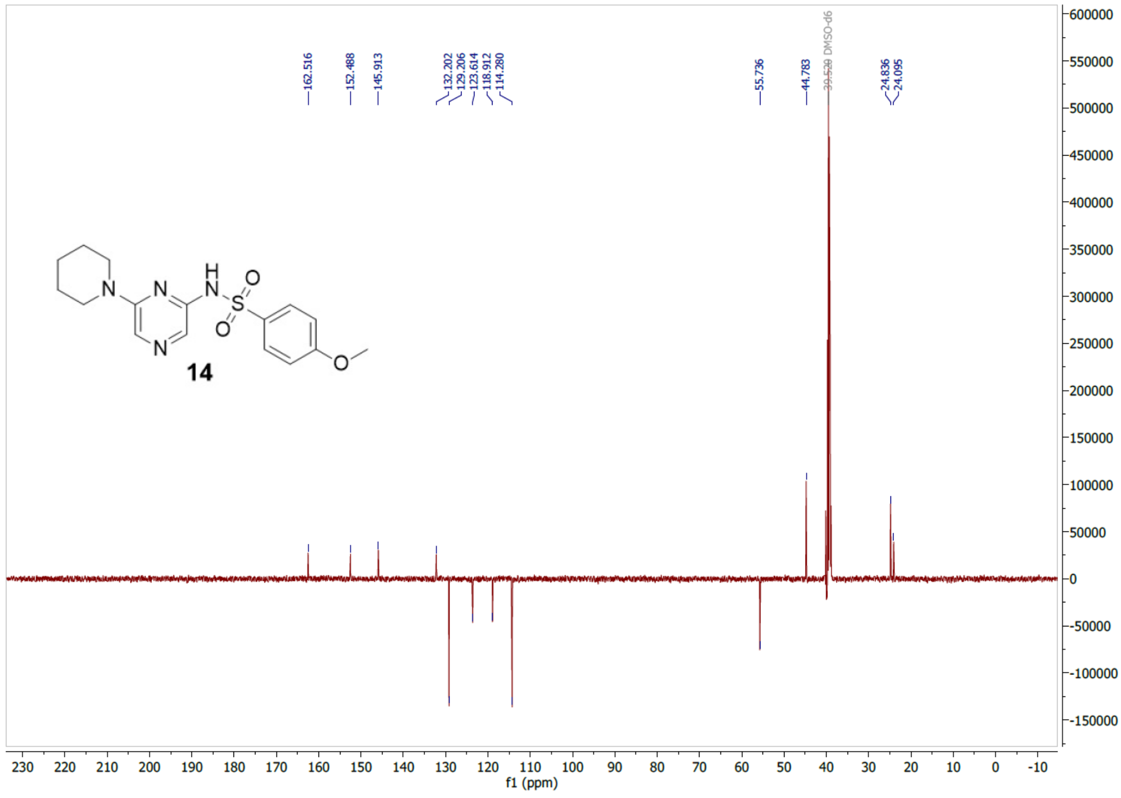
14
162.516
152.488
145.913
132.202
129.206
123.614
118.912
114.280
55.736
44.783
DMSO-d6
24.836
24.095
f1 (ppm)

==== Shimadzu LabSolutions Browser Report ====

Chromatogram(KN027_pure.lcd)

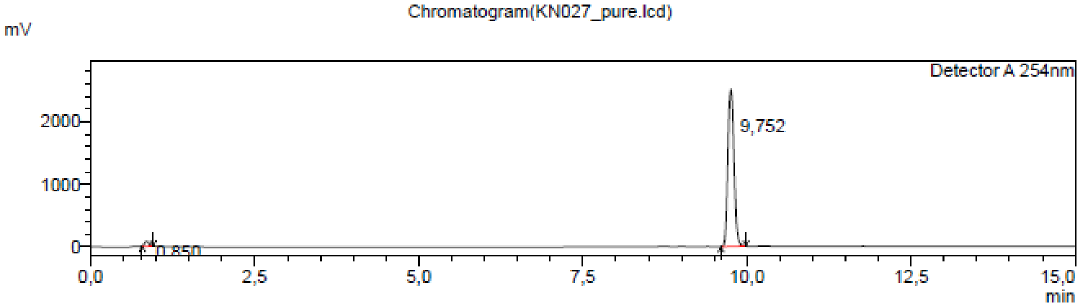

MS Chromatogram(KN027_pure.lcd)

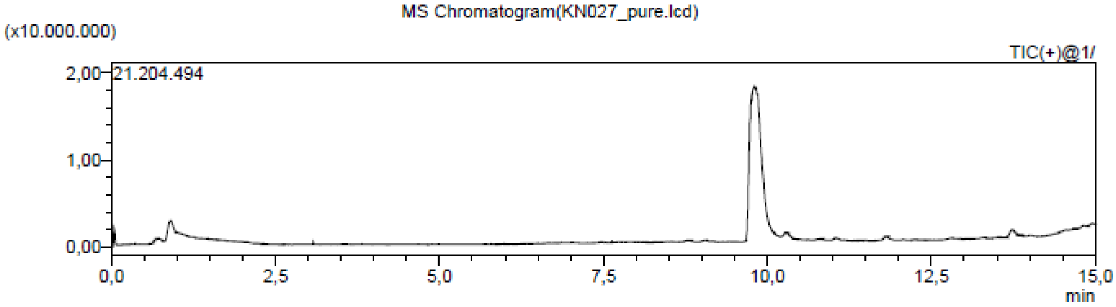

MS Spectrum(KN027_pure.lcd)

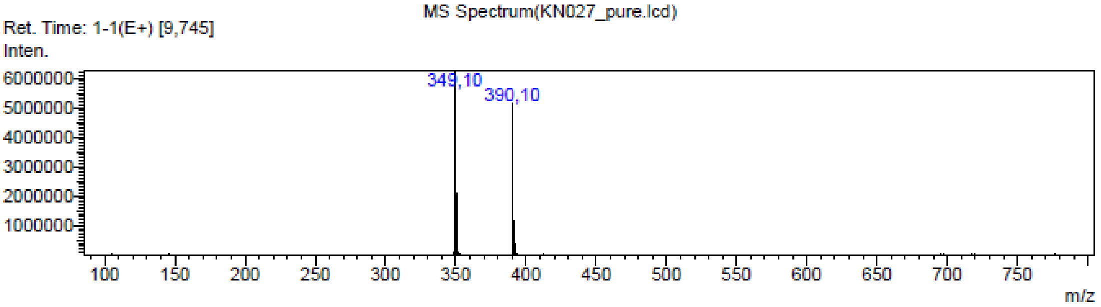

Peak Table(KN027_pure.lcd)

Detector A 254nm

Peak#	Ret. Time	Area	Height	Mark	Conc.	Area%
1	0,850	376566	79504	M	2,244	2,244
2	9,752	16402705	2499660	M	97,756	97,756
Total		16779270	2579164		100,000	100,000

Sample Information(KN027_pure.lcd)

**Data File Name:**	**KN027_pure.lcd**
**Method File Name:**	**MSgeneral1090FA_LowMW.lcm**
**Acquired by:**	**System Administrator**
**Date Acquired:**	**28-3-2023 21:54:25**
**Sample Name:**	**KN027_pure**
**Sample ID:**	**KN027_pure**
**Sample Type:**	**Unknown**
**Level#:**	**0**
**Detector:**	**Detector A, MS**
**Comment:**	

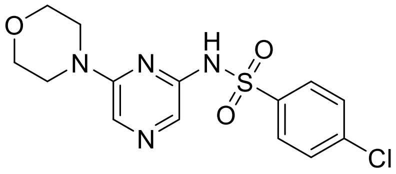

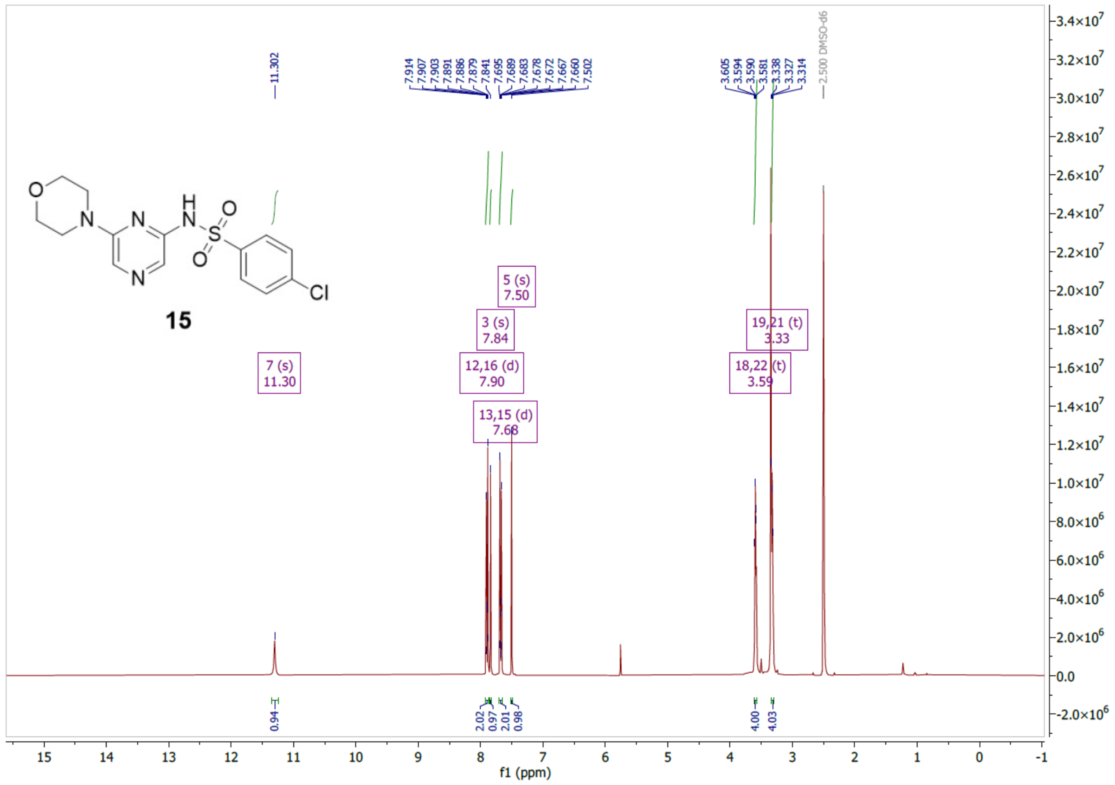
11.302
7.914
7.907
7.903
7.891
7.886
7.879
7.841
7.695
7.689
7.683
7.678
7.672
7.667
7.660
7.502
3.605
3.594
3.590
3.581
3.338
3.327
3.314
2.500 DMSO-d6
15
5 (s)
7.50
3 (s)
7.84
12,16 (d)
7.90
13,15 (d)
7.68
7 (s)
11.30
19,21 (t)
3.33
18,22 (t)
3.59
0.94
2.02
0.97
2.01
0.98
4.00
4.03
f1 (ppm)

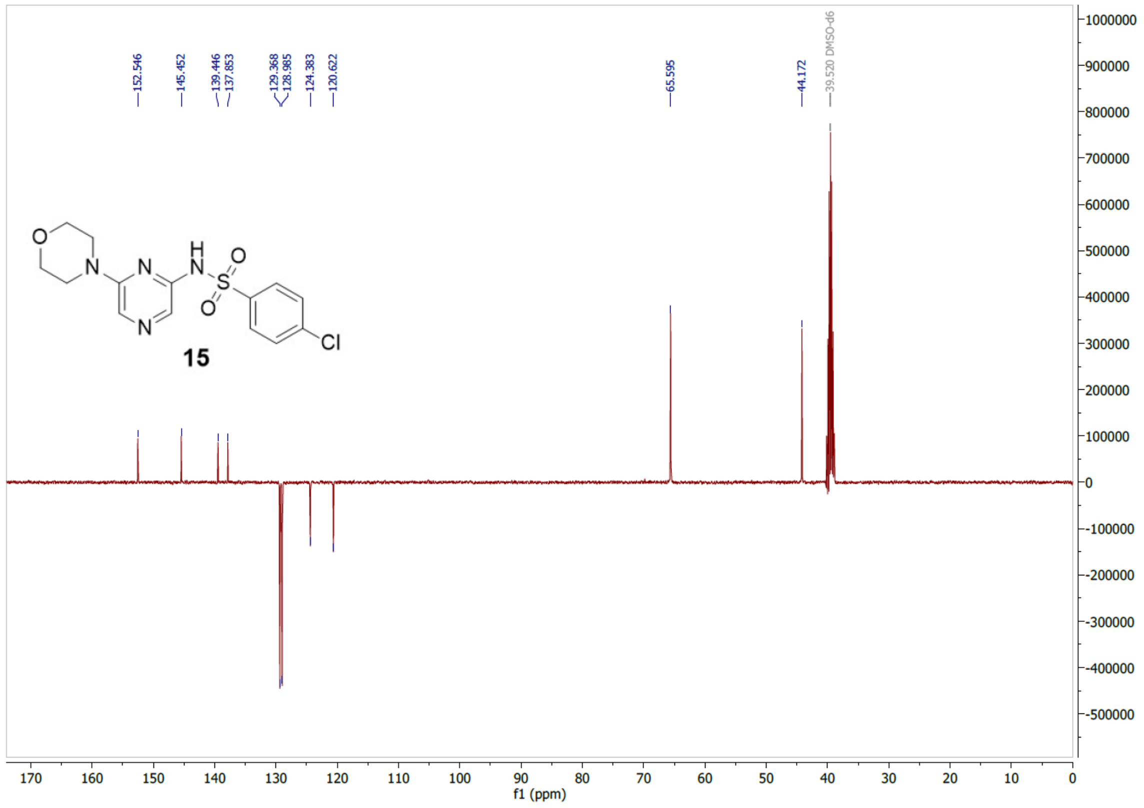
152.546
145.452
139.446
137.853
129.368
128.985
124.383
120.622
65.595
44.172
39.520 DMSO-d6
15
f1 (ppm)

==== Shimadzu LabSolutions Browser Report ====

Chromatogram(KN013_pure.lcd)

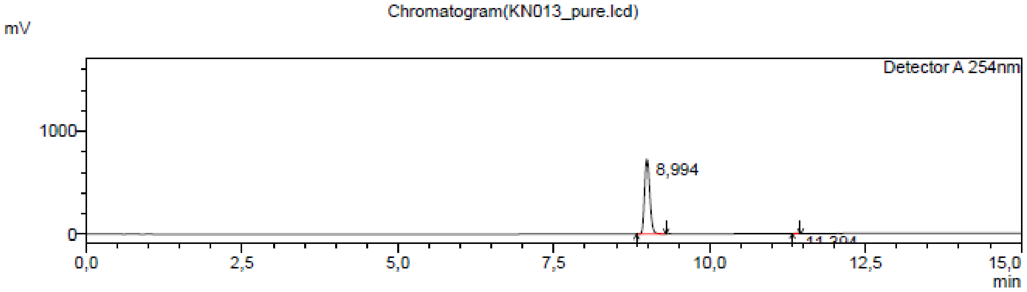

MS Chromatogram(KN013_pure.lcd)

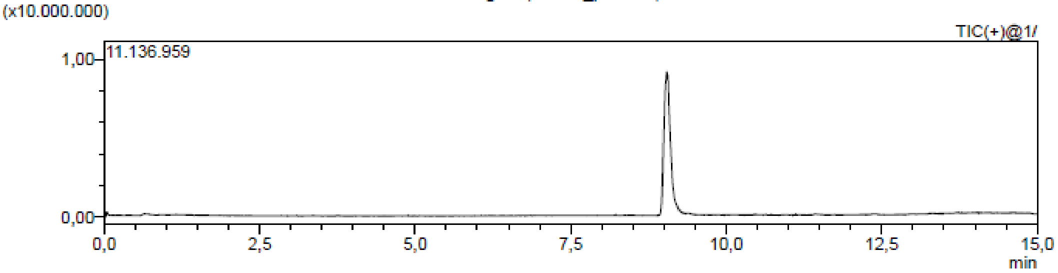

MS Spectrum(KN013_pure.lcd)

Ret. Time: 1-1(E+) [9,048]

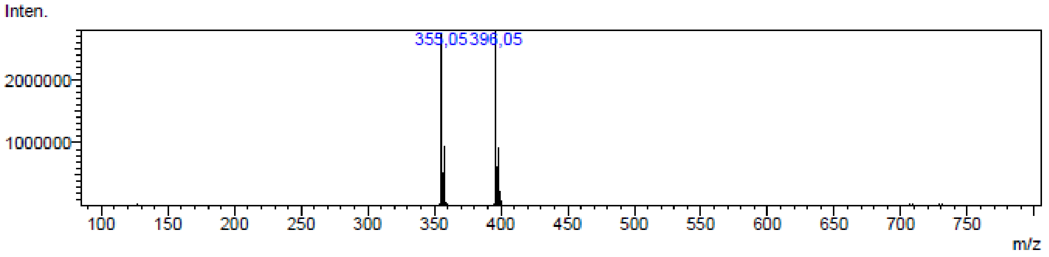

Peak Table(KN013_pure.lcd)

Detector A 254nm

Peak#	Ret. Time	Area	Height	Mark	Conc.	Area%
1	8,994	4070460	721283	M	99,782	99,782
2	11,394	8895	2010	M	0,218	0,218
Total		4079355	723293		100,000	100,000

Sample Information(KN013_pure.lcd)

**Data File Name:**	KN013_pure.lcd
**Method File Name:**	MSgeneral1090FA_LowMW.lcm
**Acquired by:**	System Administrator
**Date Acquired:**	22-2-2023 18:46:28
**Sample Name:**	KN013_pure
**Sample ID:**	KN013_pure
**Sample Type:**	Unknown
**Level#:**	0
**Detector:**	Detector A, MS
**Comment:**	

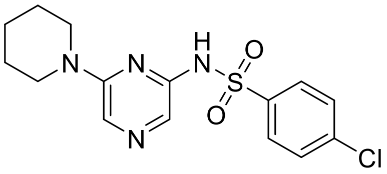

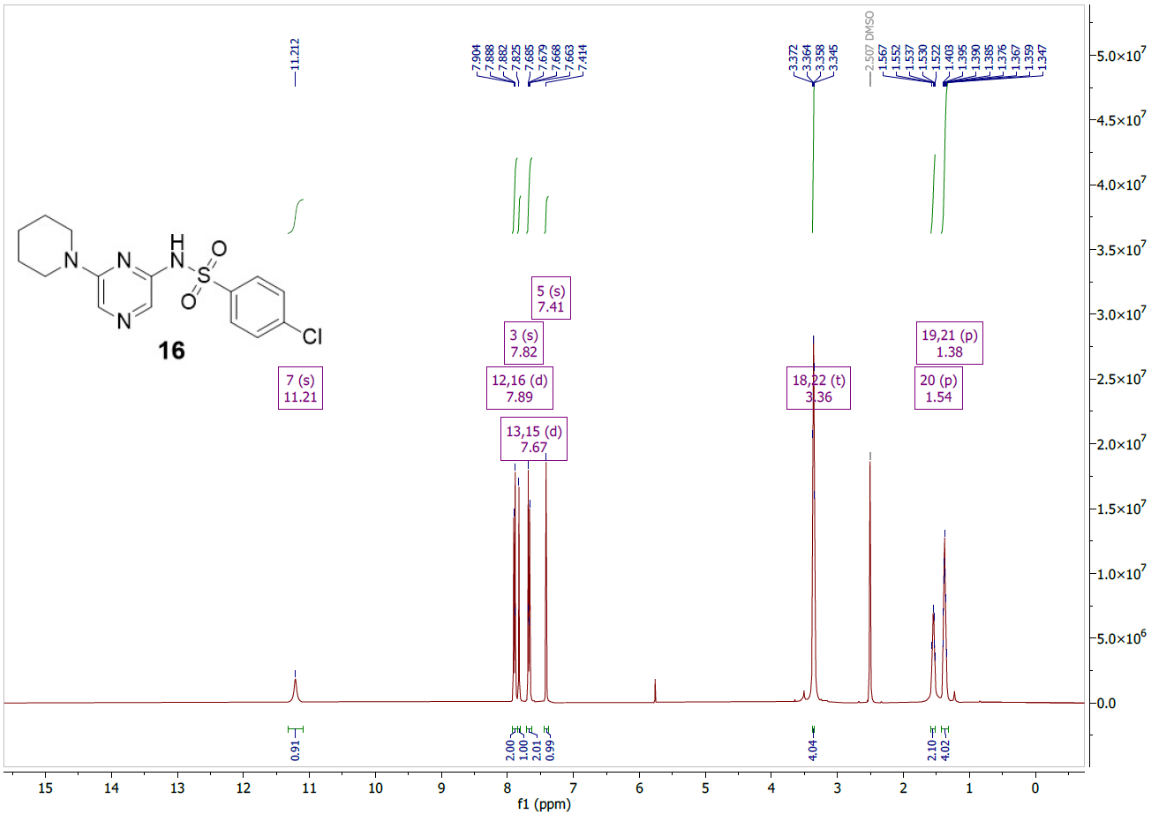
16
7 (s)
11.21
12,16 (d)
7.89
3 (s)
7.82
13,15 (d)
7.67
5 (s)
7.41
18,22 (t)
3.36
20 (p)
1.54
19,21 (p)
1.38
2.507 DMSO
f1 (ppm)

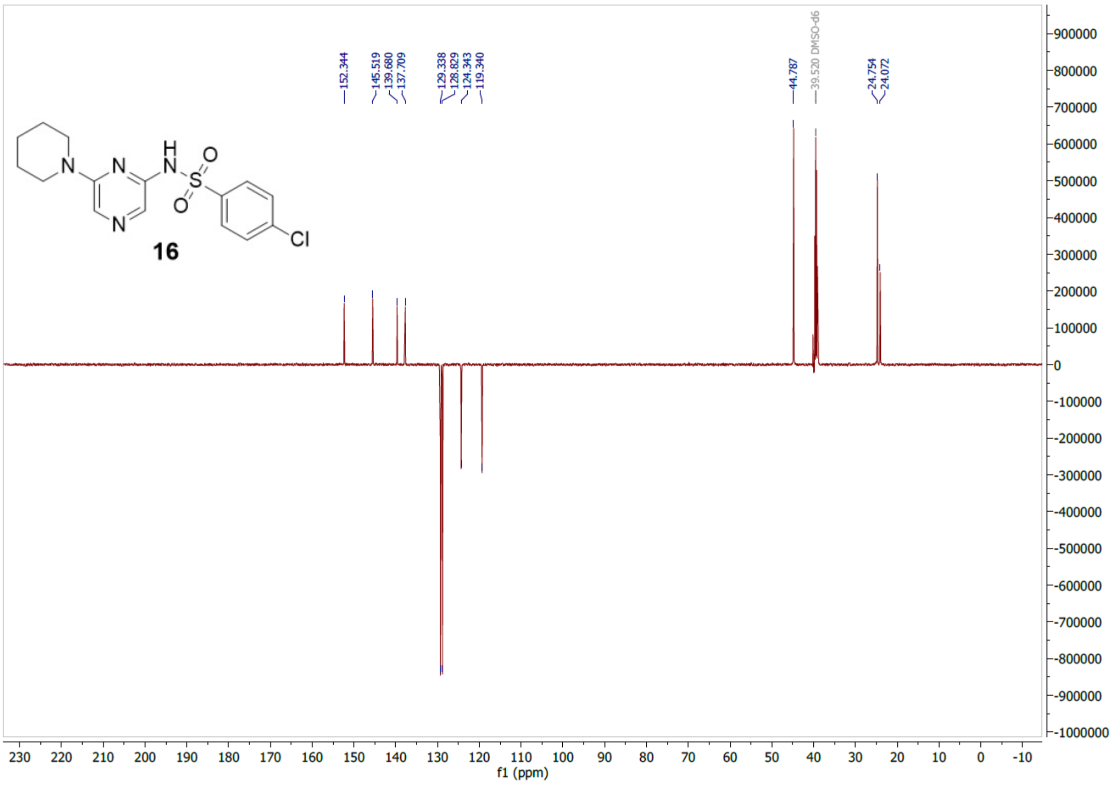
16
152.344
145.519
139.680
137.709
129.338
128.829
124.343
119.340
44.787
39.520 DMSO-d6
24.754
24.072
f1 (ppm)

==== Shimadzu LabSolutions Browser Report ====

Chromatogram(KN020_pure.lcd)

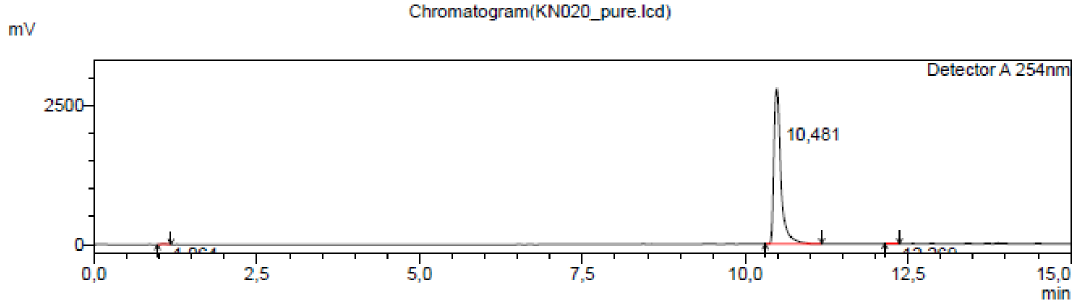

MS Chromatogram(KN020_pure.lcd)

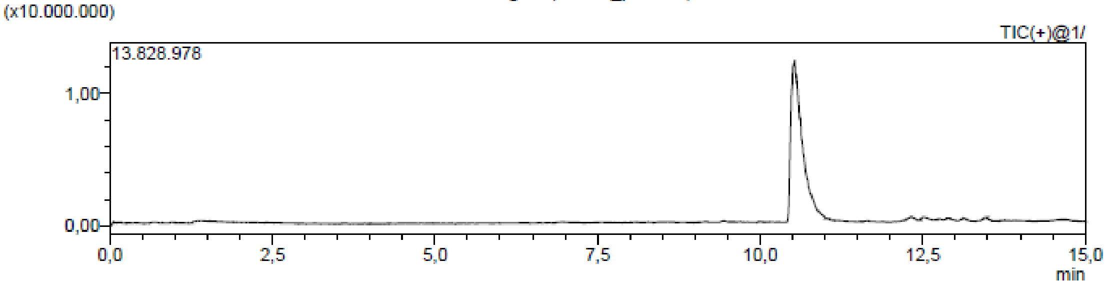

MS Spectrum(KN020_pure.lcd)

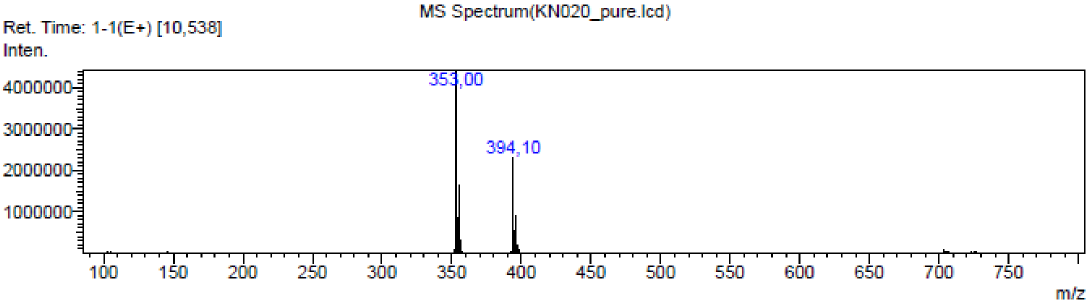

Peak Table(KN020_pure.lcd)

Detector A 254nm

Peak#	Ret. Time	Area	Height	Mark	Conc.	Area%
1	1,064	49874	6800	M	0,235	0,235
2	10,481	21113444	2790329	M	99,663	99,663
3	12,269	21483	2843	M	0,101	0,101
Total		21184801	2799972		100,000	100,000

Sample Information(KN020_pure.lcd)

**Data File Name:**	KN020_pure.lcd
**Method File Name:**	MSgeneral1090FA_LowMW.lcm
**Acquired by:**	System Administrator
**Date Acquired:**	13-3-2023 16:31:19
**Sample Name:**	KN020_pure
**Sample ID:**	KN020_pure
**Sample Type:**	Unknown
**Level#:**	0
**Detector:**	Detector A, MS
**Comment:**	

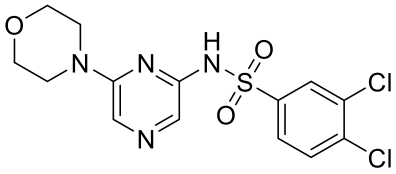
O
N
N
N
H
N
S
O
O
Cl
Cl

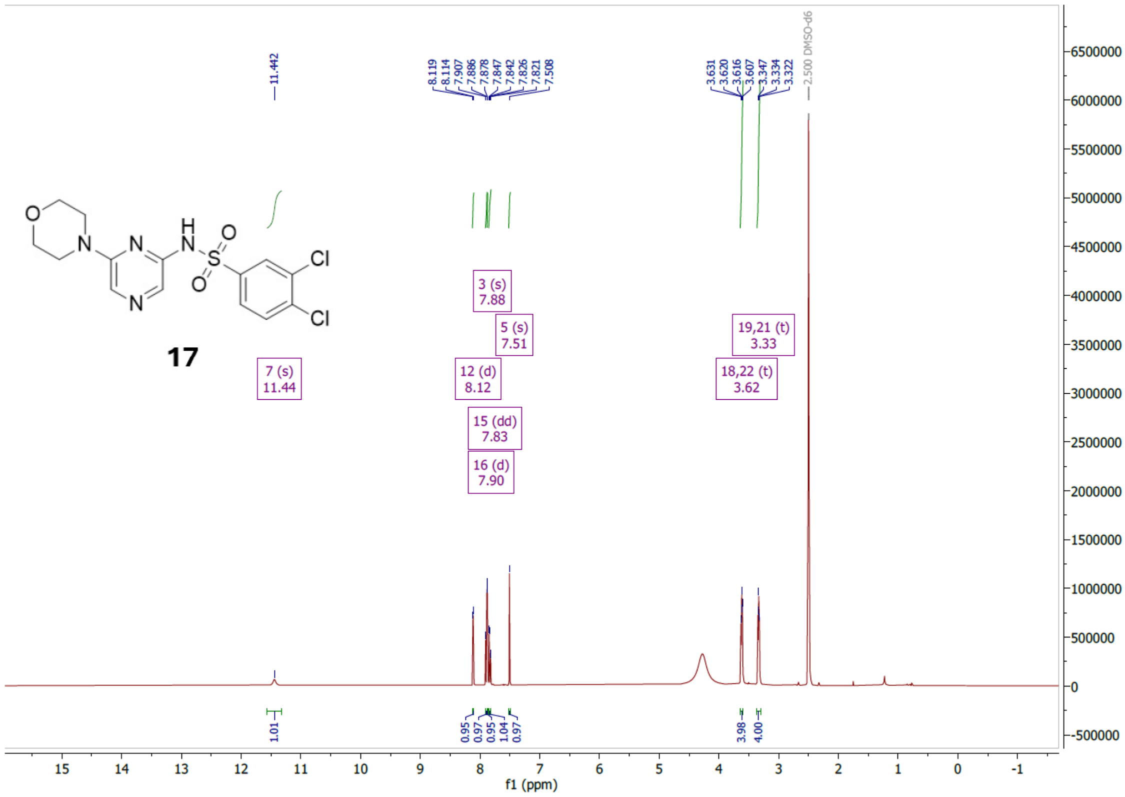
11.442
8.119
8.114
7.907
7.886
7.878
7.847
7.842
7.826
7.821
7.508
3.631
3.620
3.616
3.607
3.347
3.334
3.322
2.500 DMSO-d6
17
3 (s) 7.88
5 (s) 7.51
12 (d) 8.12
15 (dd) 7.83
16 (d) 7.90
7 (s) 11.44
19,21 (t) 3.33
18,22 (t) 3.62
1.01
0.95
0.97
0.95
1.04
0.97
3.98
4.00
f1 (ppm)

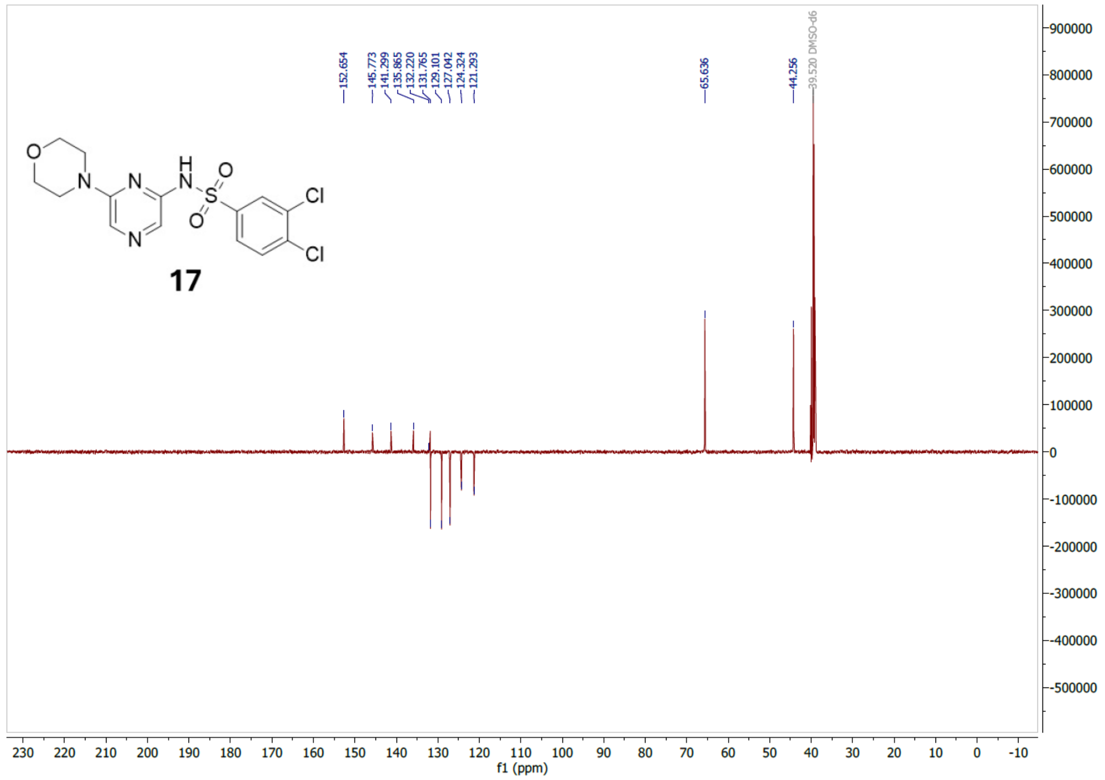
152.654
145.773
141.299
135.865
132.220
131.765
129.101
127.042
124.324
121.293
65.636
44.256
39.520 DMSO-d6
17
f1 (ppm)

==== Shimadzu LabSolutions Browser Report ====

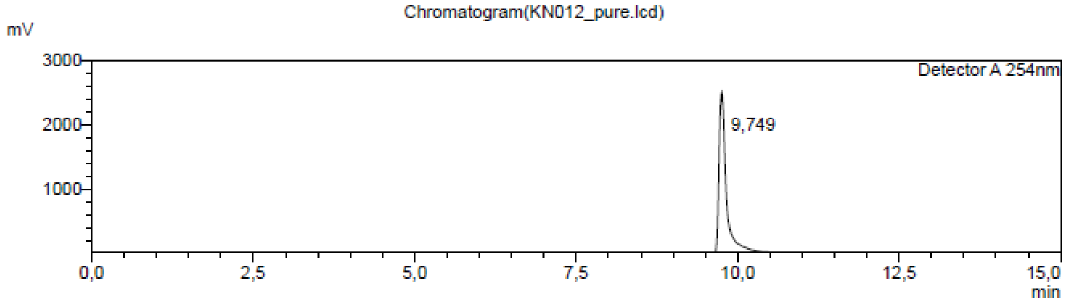

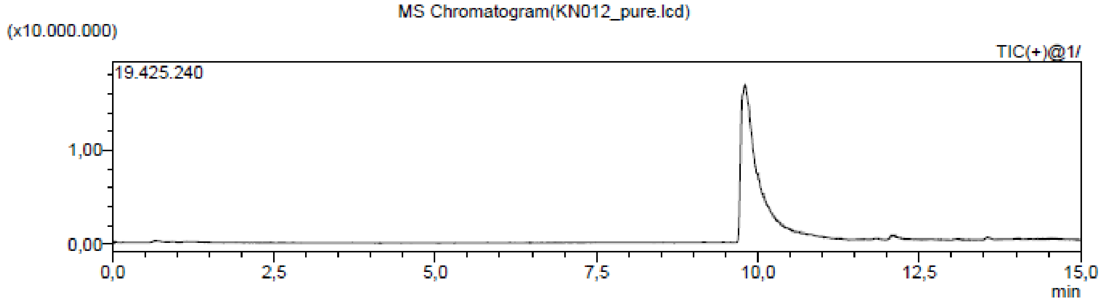

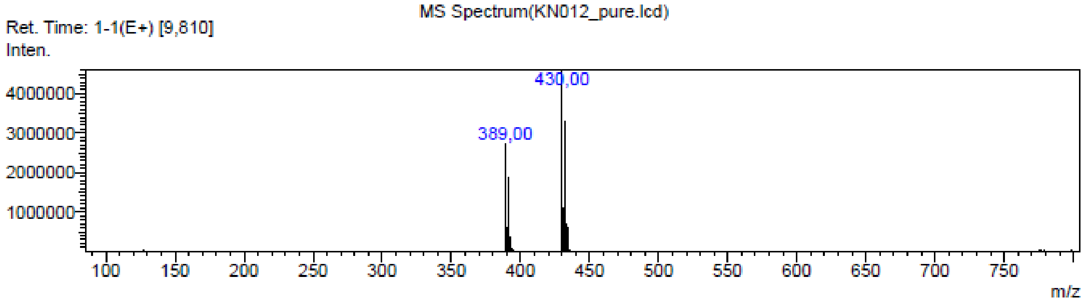

Peak Table(KN012_pure.lcd)

Detector A 254nm

Peak#	Ret. Time	Area	Height	Mark	Conc.	Area%
1	9,749	20524349	2485804	M	99,912	99,912
2	12,046	18133	4045	M	0,088	0,088
Total		20542481	2489849		100,000	100,000

Sample Information(KN012_pure.lcd)

Data File Name: KN012_pure.lcd
Method File Name: MSgeneral1090FA_LowMW.lcm
Acquired by: System Administrator
Date Acquired: 20-2-2023 16:58:06
Sample Name: KN012_pure
Sample ID: KN012_pure
Sample Type: Unknown
Level#: 0
Detector: Detector A, MS
Comment:

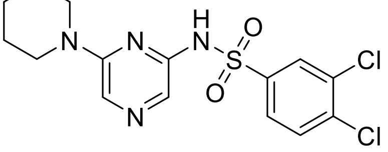

N
H
O
S
Cl

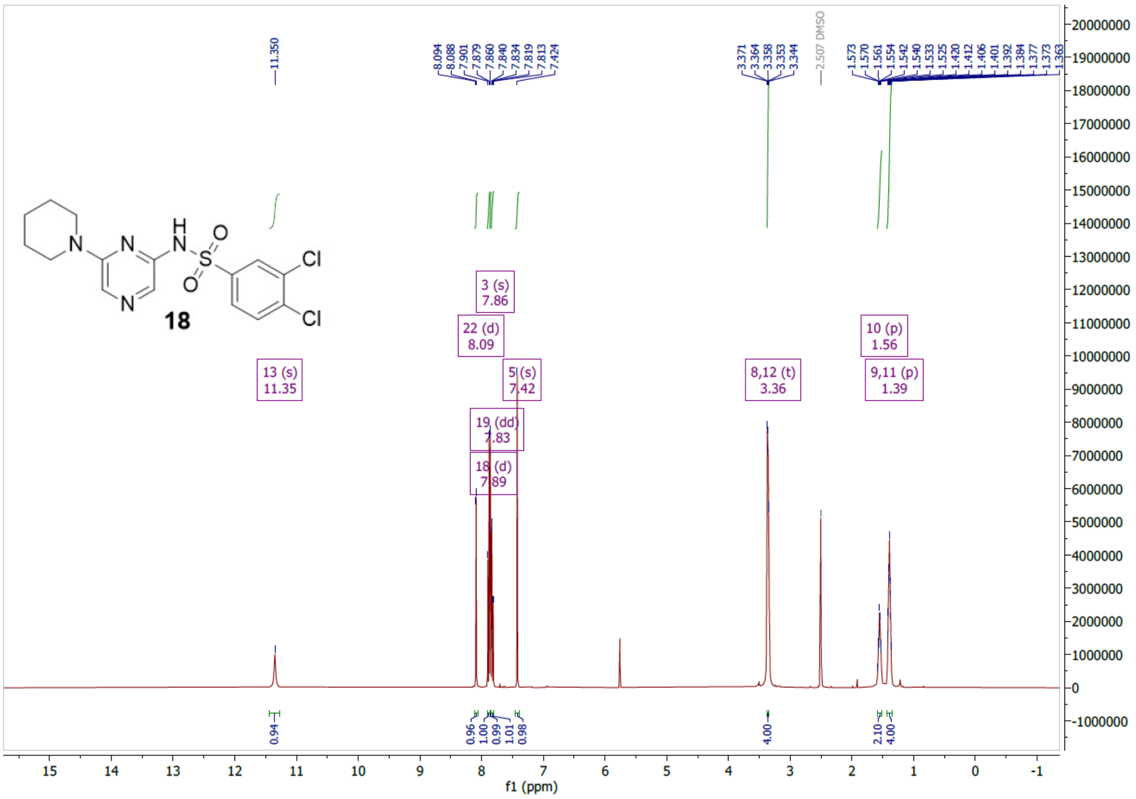

11.350
8.094
8.088
7.901
7.879
7.860
7.840
7.834
7.819
7.813
7.424
3.371
3.364
3.358
3.353
3.344
2.507 DMSO
1.573
1.570
1.561
1.554
1.542
1.540
1.533
1.525
1.420
1.412
1.406
1.401
1.392
1.384
1.377
1.373
1.363
18
3 (s)
7.86
22 (d)
8.09
13 (s)
11.35
5 (s)
7.42
19 (dd)
7.83
18 (d)
7.89
8,12 (t)
3.36
10 (p)
1.56
9,11 (p)
1.39
0.94
0.96
1.00
0.99
1.01
0.98
4.00
2.10
4.00
f1 (ppm)

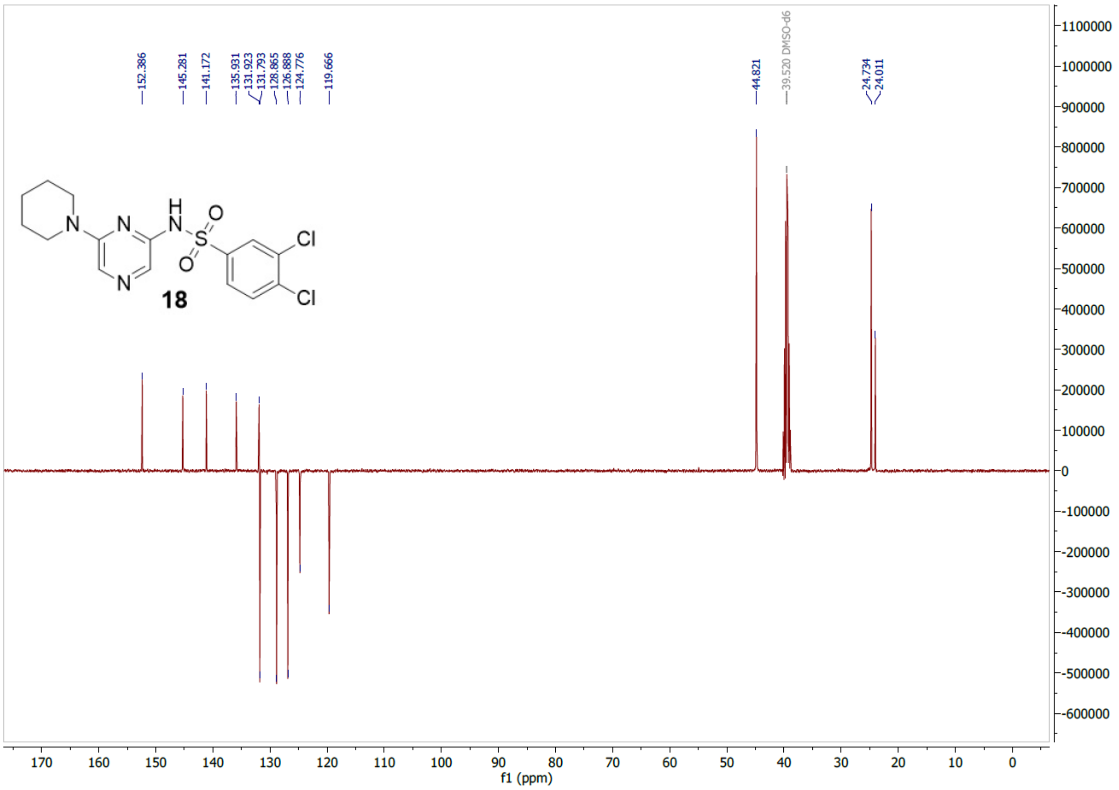

152.386
145.281
141.172
135.931
131.923
131.793
128.865
126.888
124.776
119.666
44.821
39.520 DMSO-d6
24.734
24.011
18
f1 (ppm)

==== Shimadzu LabSolutions Browser Report ====

Chromatogram(KN019_f41-57.lcd)

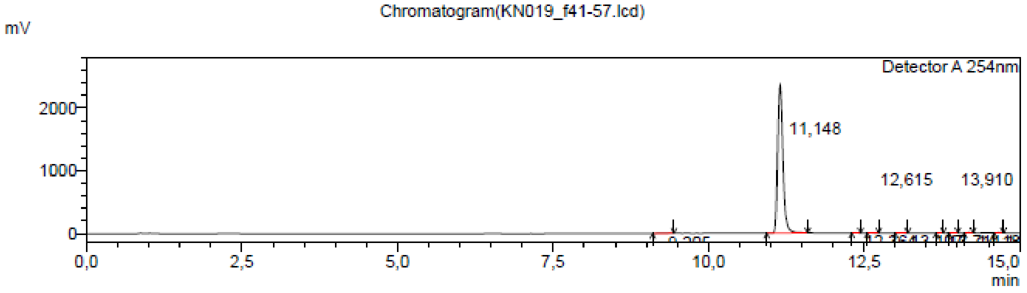

MS Chromatogram(KN019_f41-57.lcd)

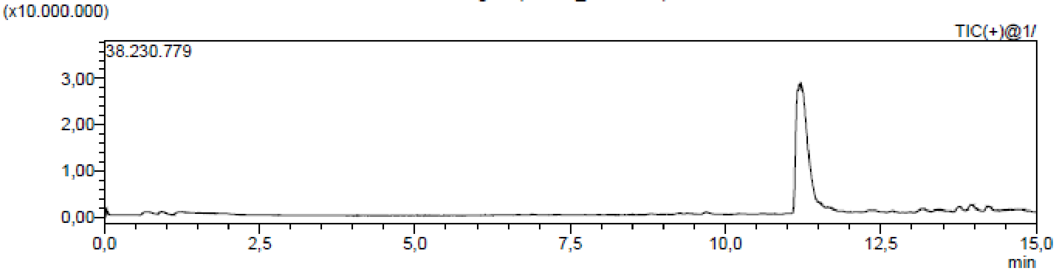

MS Spectrum(KN019_f41-57.lcd)

Ret. Time: 1-1(E+) [11,173]

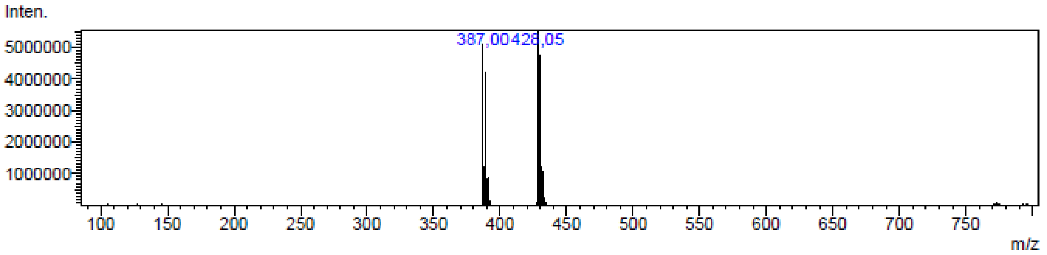

Peak Table(KN019_f41-57.lcd)

Detector A 254nm

Peak#	Ret. Time	Area	Height	Mark	Conc.	Area%
1	9,205	31290	3997	M	0,224	0,224
2	11,148	13845941	2357603	M	99,075	99,075
3	12,364	14756	3158	M	0,106	0,106
4	12,615	18103	3465	M	0,130	0,130
5	13,107	12179	1536	M	0,087	0,087
6	13,714	8417	2004	M	0,060	0,060
7	13,910	7988	1920	M	0,057	0,057

Sample Information(KN019_f41-57.lcd)

**Data File Name:**	KN019_f41-57.lcd
**Method File Name:**	MSgeneral1090FA_LowMW.lcm
**Acquired by:**	System Administrator
**Date Acquired:**	9-3-2023 16:12:56
**Sample Name:**	KN019_f41-57
**Sample ID:**	KN019_f41-57
**Sample Type:**	Unknown
**Level#:**	0
**Detector:**	Detector A, MS
**Comment:**	

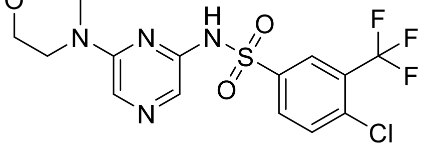

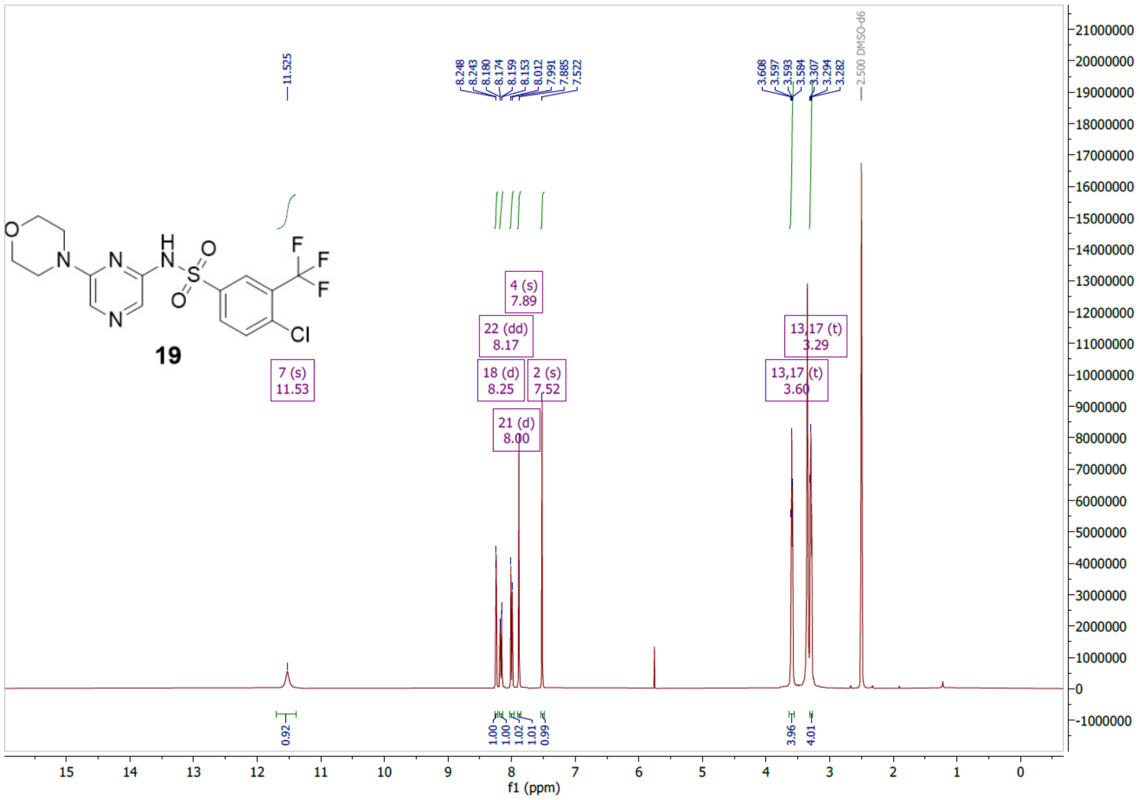

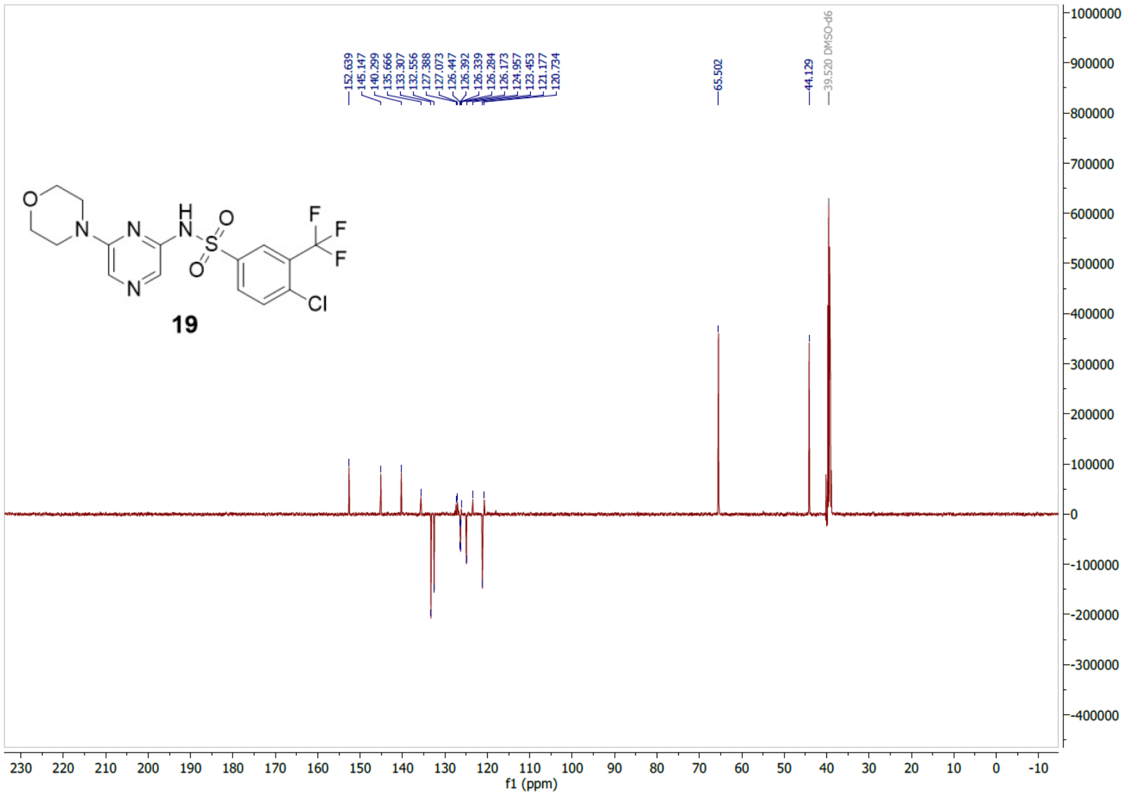

==== Shimadzu LabSolutions Browser Report ====

Chromatogram(KN011_pure.lcd)

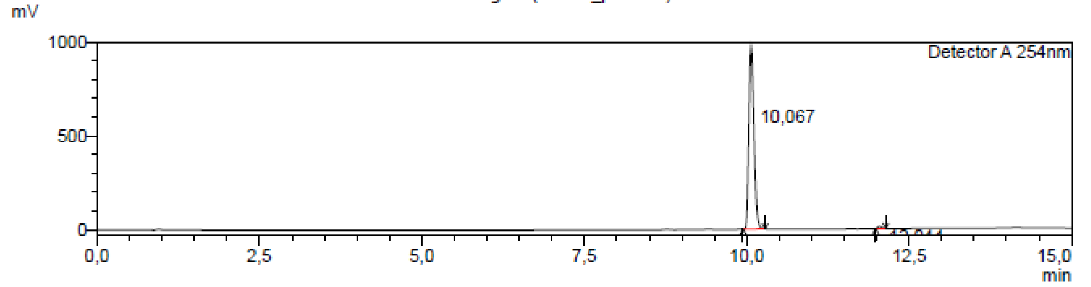

MS Chromatogram(KN011_pure.lcd)

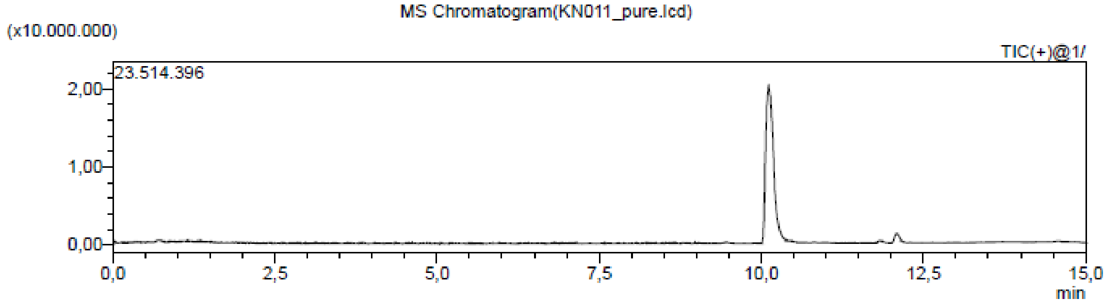

MS Spectrum(KN011_pure.lcd)

Ret. Time: 1-1(E+) [10,108]

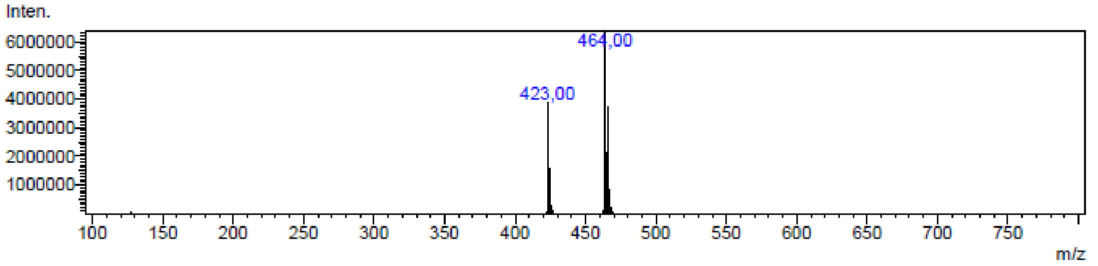

Peak Table(KN011_pure.lcd)

Detector A 254nm

Peak#	Ret. Time	Area	Height	Mark	Conc.	Area%
1	10,067	5271763	968918	M	98,954	98,954
2	12,044	55724	11858	M	1,046	1,046
Total		5327487	980776		100,000	100,000

Sample Information(KN011_pure.lcd)

**Data File Name:**	KN011_pure.lcd
**Method File Name:**	MSgeneral1090FA_LowMW.lcm
**Acquired by:**	System Administrator
**Date Acquired:**	15-2-2023 20:57:38
**Sample Name:**	KN011_pure
**Sample ID:**	KN011_pure
**Sample Type:**	Unknown
**Level#:**	0
**Detector:**	Detector A, MS
**Comment:**	

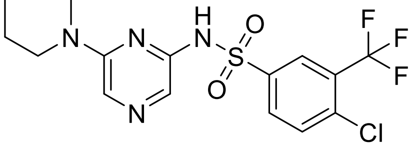

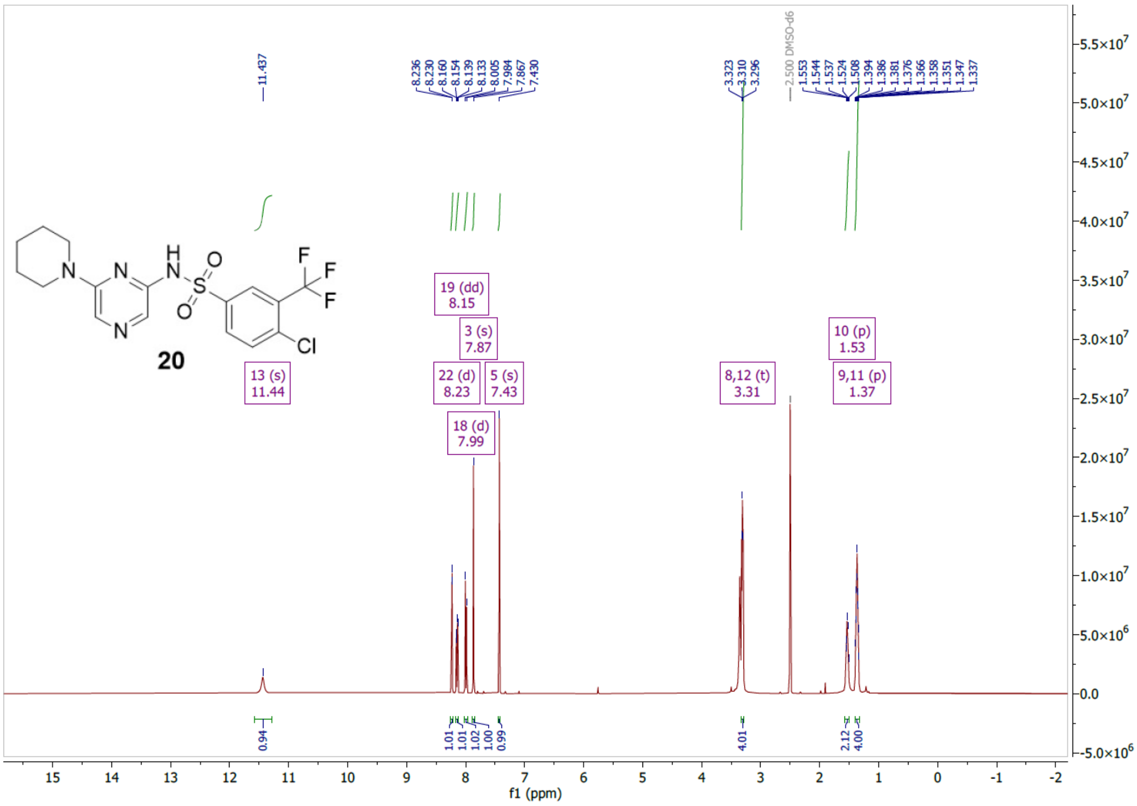

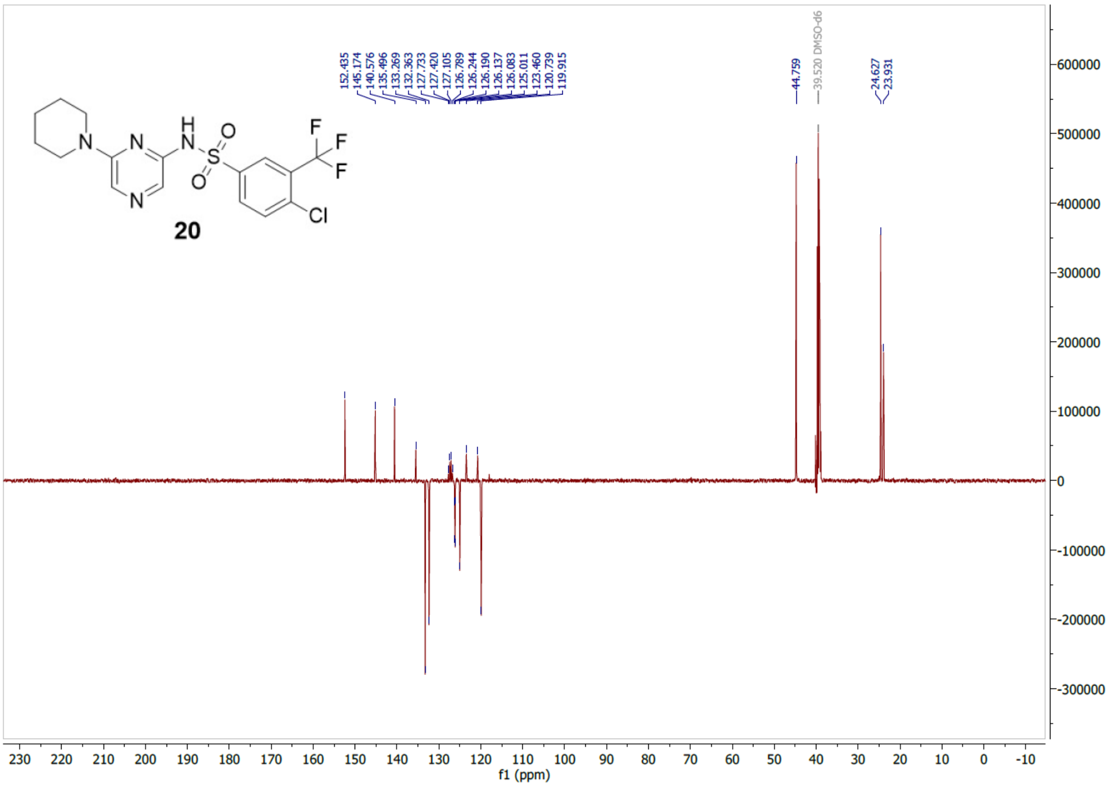

==== Shimadzu LabSolutions Browser Report ====

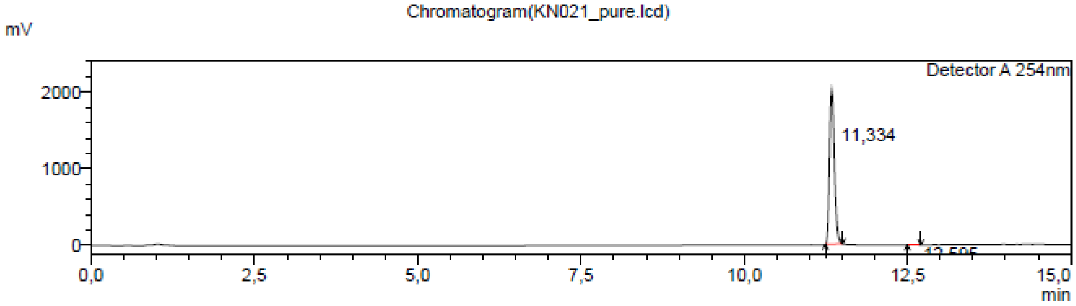

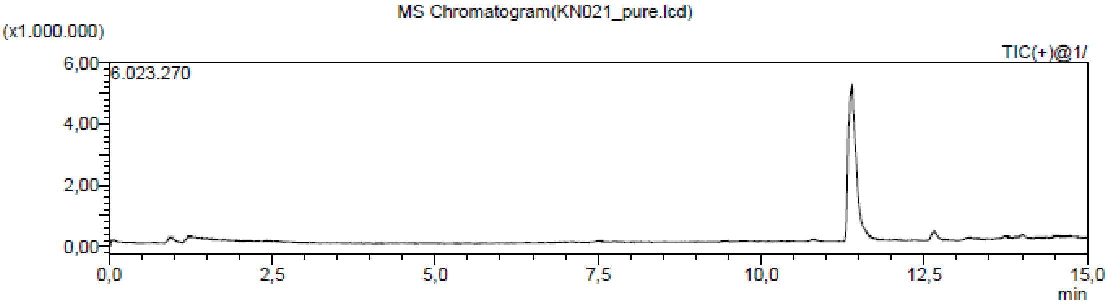

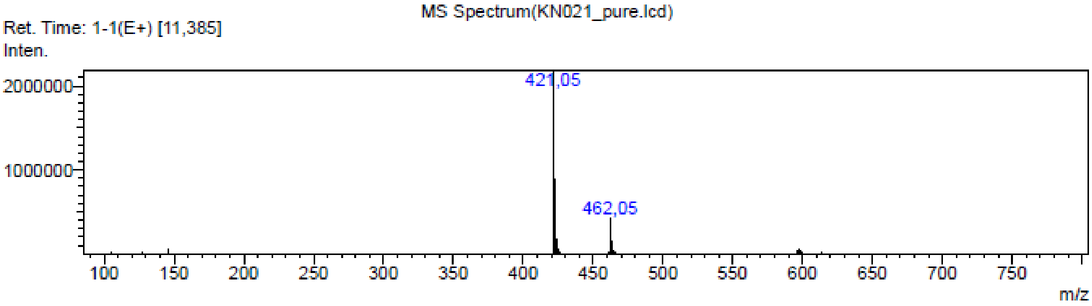

Peak Table(KN021_pure.lcd)

Detector A 254nm

Peak#	Ret. Time	Area	Height	Mark	Conc.	Area%
1	11,334	10577456	2018814	M	99,840	99,840
2	12,595	16962	2762	M	0,160	0,160
Total		10594418	2021577		100,000	100,000

Sample Information(KN021_pure.lcd)

**Data File Name:**	KN021_pure.lcd
**Method File Name:**	MSgeneral1090FA_LowMW.lcm
**Acquired by:**	System Administrator
**Date Acquired:**	15-3-2023 18:34:55
**Sample Name:**	KN021_pure
**Sample ID:**	KN021_pure
**Sample Type:**	Unknown
**Level#:**	0
**Detector:**	Detector A, MS
**Comment:**	
